# The use of Twitter by state leaders and its impact on the public during the COVID-19 pandemic

**DOI:** 10.1016/j.heliyon.2020.e05540

**Published:** 2020-11-19

**Authors:** Michael Haman

**Affiliations:** Department of Political Science; Philosophical Faculty; University of Hradec Kralové; Czech Republic

**Keywords:** Social sciences, Social media, Twitter, COVID-19, Uses and gratifications theory, Public communication, Political communication

## Abstract

The article examines how many leaders used Twitter during the COVID-19 pandemic, in what way, and the impact they had on the public. In the context of Twitter, the impact on the public refers to the growth in followers as it signifies the increased interest of the public about information. 50,872 tweets were collected from 143 state leaders and an original dataset was created containing information on the growth of followers. Ordinary least squares regression models were used for the analysis. It was found that 64.8% of UN member states had a leader that tweeted about COVID-19. Furthermore, a significant increase in the number of followers during the pandemic compared to months prior was noted. Since March, the pandemic has been a dominant topic on Twitter. During the COVID-19 pandemic, the highest percentage increase in gaining Twitter followers was experienced by politicians who frequently tweeted and those who had a lower ratio of the number of followers to internet users. The research implies that citizens are interested in being informed about emergencies through social networks, and government officials should use them.

## 1 Introduction

Coronavirus disease 2019 (COVID-19) was declared a pandemic by the World Health Organization on 11 March 2020. COVID-19 is transmitted quite effectively and is primarily spread by contact transmission and respiratory droplets (Gates, 2020; Kuca, 2020). State leaders had to react to this situation and inform citizens about measures they should take to prevent spreading COVID-19. They may, also, have wanted to inform citizens about government measures. There are many ways to inform the public. For example, leaders may choose to inform the public via social networks, as it is an ideal way to inform people swiftly. Social networks provide the possibility to inform the public in real-time about a situation. In emergencies, such as the spread of COVID-19, it is crucial to quickly inform the public. Twitter is a social network with 330 million monthly active users (Statista, 2019). There is no doubt that some leaders have used Twitter to inform the public. This article analyzes how many leaders use Twitter to inform the public, how they use it, and how this use may have impacted the public. More specifically, it is focused on the growth of Twitter followers.

Several mass communication theories are used and applied to social media (Sheldon, 2015). One of these theories is the uses and gratifications theory (UGT; Katz et al., 1973) that posits that people use certain media depending on their needs. People want to fulfill their needs. Social and psychological needs also differ for each individual and are affected by different factors. In this study, Twitter is examined in times of crisis. One would assume that during a health crisis people would be more information seeking. Casero-Ripollés (2020) confirms that news consumption increased immensely during COVID-19. Igartua et al. (2020) find that high consumption of information about COVID-19 raises perceived knowledge on the topic, and it results in the adoption of preventive measures. People can use Twitter as a source of news.

Parmelee and Bichard (2012) examine why people follow political actors on Twitter. They find that there are several motives for using Twitter: convenience, entertainment, self-expression, guidance, information-seeking, and social utility. These motives correspond with the UGT. During the pandemic, several of these motives are present. Twitter is a convenient platform for information as individuals only need a mobile or computer screen. Guidance is important when individuals need advice from relevant actors on how to act. For example, the recommendation via Twitter from U. S. governors for residents to stay at home greatly reduced the mobility of individuals during the COVID-19 pandemic (Grossman et al., 2020). Information-seekers can easily find out what government measures are in place to combat the pandemic and the overall state of the country under the assumption that the government account offers this information. The information obtained by individuals has also a social utility as individuals then can discuss with their family or friends what they learned from Twitter. Many studies have confirmed that certain users use Twitter primarily as an information-seeking medium that fulfills their need for information (Hughes et al., 2012; Johnson and Yang, 2009). Of course, the UGT is not used only with Twitter (Chen, 2011; Johnson and Yang, 2009), but numerous studies have examined other social media such as Facebook (Hollenbaugh and Ferris, 2014; Hunt et al., 2012; Sheldon, 2008; Smock et al., 2011) or Instagram (Pittman and Reich, 2016; Sheldon and Bryant, 2016).

In recent years, research on political communication through Twitter has become more prominent in social science research (Dang-Xuan et al., 2013; Enli and Skogerbø, 2013; Jungherr, 2015, 2016). Twitter is an important platform because policymakers can use its analytics to gain insights (Joseph et al., 2017). Unsurprisingly, there has been research on the adoption of social media by world leaders (Barberá and Zeitzoff, 2018). Also, it is important to examine communication on Twitter by political leaders as the research on daily newspapers in Spain and Italy has shown that the protagonists of the pandemic are especially politicians (Tejedor et al., 2020). The research on the usage of Twitter during the COVID-19 pandemic is ongoing, but there have been already several studies analyzing the impact of COVID-19 on Twitter usage.

Numerous studies have been published that examined spreading misinformation on social media during the COVID-19 pandemic (Gruzd and Mai, 2020; Lovari, 2020; Pérez-Dasilva et al., 2020; Pulido et al., 2020; Rodríguez et al., 2020; Vraga et al., 2020). For example, Pulido et al. (2020) find that false information about the pandemic is tweeted more but retweeted less than science-based tweets. Bridgman et al. (2020) find that exposure to social media is linked with misperceptions concerning basic facts about COVID-19. Two studies have been done on the political polarization during the pandemic. In Canada, Merkley et al. (2020) find that the political elites and the public are in a time of cross-partisan agreement on the important matters (for example social distancing). On the contrary, in the United States, tweets are characterized by strong political polarization (Jiang et al., 2020). Several studies have analyzed tweets and their content during the pandemic (Abd-Alrazaq et al., 2020; Budhwani and Sun, 2020; Jimenez-Sotomayor et al., 2020; Park et al., 2020; Rao et al., 2020; Su et al., 2020; Thelwall and Levitt, 2020), and others have used the sentiment analysis (Manguri et al., 2020; Pastor, 2020; Toliyat, 2020). In China, Chen et al. (2020) examine the importance of national government on social media by analyzing citizen engagement through social media of Chinese central agencies.

However, there has been only one study that examined state leaders and their activity on Twitter and this was limited to the world leaders of the Group of Seven (G7). The study shows, alongside other findings, that world leaders of the G7 all use Twitter except Angela Merkel (Rufai and Bunce, 2020). It may be beneficial for world leaders to use Twitter during crises and to gain followers that could become their potential supporters. Support of governments has, on average, risen among populations during the COVID-19 pandemic (Bol et al., 2020). Also, news consumption explains support for policies that seek to limit the spread of COVID-19 (Cilizoglu et al., 2020). Therefore, more information from relevant government sources may help to improve citizens' compliance with government actions.

This article is unique because a study has not yet been presented that analyzes the global use of Twitter by state leaders and the public's reaction to it. Therefore, it is a significant contribution to the literature that examines the situation during the COVID-19 pandemic. The article is divided into four main parts. The first part offers information on the methods used and has two subsections. The first subsection concerns with data collection and the second subsection explains regression models. The second part presents the results with two subsections. The first subsection offers insights from social media analytics while the second subsection shows insights from regression models. Therefore, the first subsection presents mainly figures and tables about the usage of Twitter during the pandemic by state leaders. Thus, it is focused on the visual presentation of the activity of world leaders. In the second section, the explanation about the growth rate of Twitter followers on leaders' accounts is offered. Regression models were used to determine the reasons for the growth rate of Twitter followers. The third part discusses contribution to theory and literature, implications to practice, limitations and suggests avenues for future research. In the last part, the core findings are mentioned.

## 2 Methods

In recent years, big data research and their challenges have been the subject of literature (Abbasi et al., 2016; Fang et al., 2015; Kar and Dwivedi, 2020). This study uses methodological approaches that are based on big data. However, to combat challenges linked with big data research, the study uses statistical techniques commonly used in social science. Also, the study is connected to a theory that is often a problem with big data studies.

### 2.1 Collection of Twitter data

Twitter API was used to create the original dataset of state leaders' tweets. 50,872 tweets that were sent from 30 December 2019^1^ to 7 May 2020 from 143 state leaders were collected and analyzed. The R package rtweet (Kearney, 2018) was used to collect tweets. In this article, the term “state leader” is defined as either Head of State or Head of Government with main executive power. Therefore, for example, monarchs with ceremonial power or Presidents in parliamentary systems are not included in the analysis.^2^ Furthermore, only what one may consider personal Twitter accounts were analyzed, not the office or press secretary Twitter accounts. For example, in the case of Donald Trump, his personal account @realDonaldTrump was used and not @POTUS or @WhiteHouse. In recent years, @realDonaldTrump has been the subject of many studies (Lacatus, 2020; Pérez-Curiel and Naharro, 2019; Yeste and Franch, 2018). However, in some countries, it is not easy to distinguish the Twitter account of politicians from the account used by the office that she or he represents. These differences may be significant as politicians keep their personal accounts while they lose access to the office accounts when they are defeated in elections and removed from the office. Moreover, personal accounts may have different dynamics as these accounts are used for campaigning while the office accounts should remain neutral. The collection of data about the Twitter accounts of world politicians was made easier by Twiplomacy (BCW, 2020). Twiplomacy provides the database of politicians and world organizations and collects the accounts on social media. Therefore, this website is a great help in the political analysis of social media. However, all recorded accounts were manually re-checked and verified whether unrecorded politicians do not use Twitter. It was possible to collect all tweets through Twitter API. However, Twitter does not offer information on the growth of followers retrospectively. Therefore, data on the number of followers were continuously collected for the majority of state leaders. Hence, it was possible to use the information on the number of Twitter followers in the past and calculate the growth. However, data for some accounts with a small number of followers were not available as data on these state leaders were not continuously collected. The study had available complete data regarding the growth of followers for more than 83% of politicians including a collection of their tweets. Only partial data for some politicians were available, but these data were sufficient^3^ to include them in regression models that are introduced in the following section.

### 2.2 Regression models – dependent and independent variables

**Figure 1:**
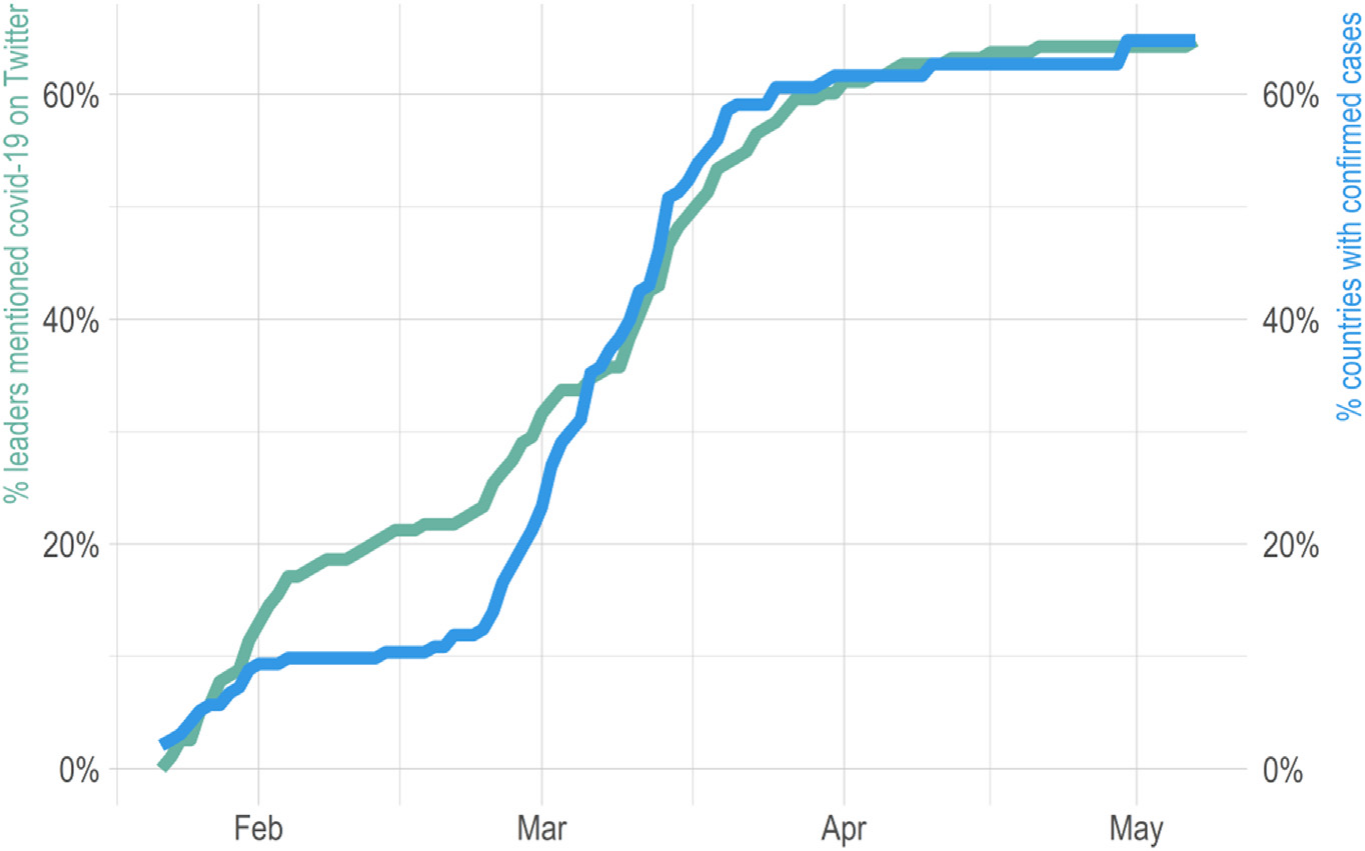
Mentions of COVID-19 on Twitter by leaders in relation to confirmed cases in countries.

The dependent variable is the growth of Twitter followers in percentage points from 27 January to 3 May 2020. This period was chosen because it covers the outbreak of COVID-19 and the spread of confirmed cases around the world. At the beginning of May 2020, almost every country had already put some measures in place to curb the spread of COVID-19. Moreover, at that time, every state leader analyzed in this study had already tweeted about the pandemic, as Figure 1 in the next section shows. Therefore, the need for information was critical. Simply put, if people were interested in being informed about the pandemic via Twitter, it would make sense that they would follow a Twitter account of a relevant state leader at the beginning of May 2020.

As it has been already mentioned, detailed information about the growth rate of Twitter followers for all state leaders was not for the purpose of study available. Furthermore, state leaders that took office in January 2020 and later were filtered out because it would skew results as one would expect there to be an increase of Twitter followers based on their win. Also, leaders who did not mention COVID-19 on Twitter were filtered out. Therefore, in the regression models, 113 leaders from UN member states were included. There may be various reasons that explain the growth of Twitter followers. Four predictor variables were chosen.

The first independent variable is the number of tweets^4^ from 27 January to 3 May 2020. In the models, tweets were not filtered. Therefore, they may include also non-COVID tweets. However, COVID-19 pandemic was the main topic. Therefore, tweets related to the pandemic dominated. Furthermore, original tweets and retweets were distinguished. The first model included all tweets and the second model only original tweets. There are good reasons to distinguish tweets. First, people usually follow more than one Twitter account. Therefore, it can be expected that during the health crisis, citizens may follow Twitter accounts of a state leader, national government, health, and other relevant authorities. If state leaders only retweet, there is a good chance that they would retweet tweets from authorities that their Twitter followers already follow. Therefore, their followers would not receive new original information. Second, in general, the content of tweets may differ. For example, based on content analysis of tweets during the 2009 H1N1 outbreak, Chew and Eysenbach (2010) find that retweets contained significantly fewer tweets with personal experience compared to original tweets. Third, at least some state leaders may consider original tweets and retweets as a slightly different activity. Donald Trump, the most followed state leader on Twitter, has been recently asked by NBC News journalist about his retweet regarding a conspiracy theory about Joe Biden. Trump responded, “That was a retweet, that was an opinion of somebody, and that was a retweet. I'll put it out there, people can decide for themselves, I don't take a position.” (Watson, 2020).

One may assume that the world leaders with greater Twitter activity gain more followers than leaders who use Twitter very little. State leaders with a very high number of followers are limited in the possibility of the growth of Twitter followers. Therefore, this possibility was measured for growth in the following way: number of followers as of 27 January 2020/number of users of internet in the country x 100. Here, the ratio of the number of followers to internet users was calculated as a percentage. Data on internet usage in a country was provided by the International Telecommunications Union (2019). One may expect that it would be more likely that world leaders would gain more followers when the ratio was fairly low, especially when leaders would use Twitter actively because this would mean that citizens would see Twitter as a new source of information. The impact of the COVID-19 pandemic was not equal across countries. Some countries were affected more heavily. Therefore, some may assume that this could disproportionality affect the need for information from citizens. The impact was measured as follows: the number of confirmed cases of COVID-19 as of 3 May 2020/population of a country x 1,000,000. Here, the number of cases per one million people was calculated. Furthermore, all variables, as all of them were heavily skewed, were logged^5^.

Another important factor in the usage of Twitter is the language that is used. Users tweeting in English may attract more attention. Therefore, it is possible that leaders tweeting in global languages can have an advantage in also gaining followers from other countries. This may be particularly in the case of neighboring countries with the same language. Citizens follow foreign leaders to see how the virus might have spread in neighboring countries but also world leaders that use global language. Therefore, four languages were chosen that might have a significant impact on the boosting of followers. These were English, Spanish, French, and Arabic. Therefore, the fourth variable, language, is a categorical variable. All other languages used by world leaders were included in the group “other languages”. Even though there were languages that are widely spoken in the group “other languages,” they were used only by a few leaders^6^, and they did not seem to have properties (being used in several neighboring countries or significantly across the globe) that could impact the significant increase of followers. When state leaders used more than one language, the language they used most frequently in their tweets was chosen. In relation to models, ordinary least squares (OLS) regression was used.

## 3 Results

### 3.1 Insights from social media analytics

The first insight from social media analytics concerns with the first mention of COVID-19 on Twitter. It is important to examine when the state leaders first mentioned COVID-19 on Twitter. To do this, several keywords, such as “COVID,” “corona,” “virus”, were looked for within the accounts of leaders. However, many leaders use a non-Latin script. Therefore, the words coronavirus and virus were translated into all languages. After that, tweets found corresponded with COVID-19 were manually re-checked. Leaders, by mentioning COVID-19 on Twitter, acknowledge it as a significant threat. However, not all leaders use Twitter in the same fashion. While leaders who frequently use Twitter might have informed people about the danger of COVID-19 when the disease remained mostly in China and a few other countries, others waited until they started putting security measures in place in their own countries before they informed citizens. Therefore, how swift people were informed about COVID-19 might differ significantly. Table 1^7^ shows leaders who informed the public already in January.

**Table 1:** First mentioned COVID-19.

21 January	Prime Minister Scott Morrison (Australia) President Tsai Ing-wen (Taiwan)
23 January	Prime Minister Andrej Babiš (Czechia Republic)
24 January	Prime Minister Lee Hsien Loong (Singapore) Prime Minister Saadeddine Othmani (Morocco) President Donald Trump (United States)
26 January	Prime Minister Justin Trudeau (Canada) President Kassym-Jomart Tokayev (Kazakhstan) President Moon Jae-in (South Korea) President Gotabaya Rajapaksa (Sri Lanka) Prime Minister Abe Shinzo (Japan)
27 January	President Joko Widodo (Indonesia)
28 January	President Lenín Moreno (Ecuador) President Alberto Fernández (Argentina) Prime Minister Andrew Holness (Jamaica) Prime Minister Hubert Minnis (Bahamas)
29 January	Prime Minister Lotay Tshering (Bhutan)
30 January	Prime Minister Giuseppe Conte (Italy)
31 January	President Laurentino Cortizo (Panama) Prime Minister Mark Rutte (Netherlands) President Jair Messias Bolsonaro (Brazil) Prime Minister Pedro Sánchez (Spain) President Alejandro Giammattei (Guatemala)

*Note:* Taiwan is not a UN member state.

Figure 1 shows the percentage of leaders who tweeted about COVID-19 across a timeline. It indicates that 64.8% of United Nations (UN) member states had leaders that tweeted about COVID-19 as of 7 May 2020. Furthermore, the second line (blue) is drawn that offers information about confirmed cases in the countries. The second line does not refer to all (193) UN member states. It refers only to countries where leaders tweeted about COVID-19. For clarity, the scales are the same. Therefore, 64.8% confirmed cases (blue line) signify that 100% of the included member states had confirmed cases. From Figure 1, it is clear that while only some state leaders considered it important to share information about COVID-19 in January, the majority of leaders were tweeting about COVID-19 in mid-March. Understandably, this was because COVID-19, by then, had begun to spread in most African, Asian, European, and Latin American countries that were not affected early. The primary source of data about confirmed cases was China Data Lab (2020).

**Figure 2:**
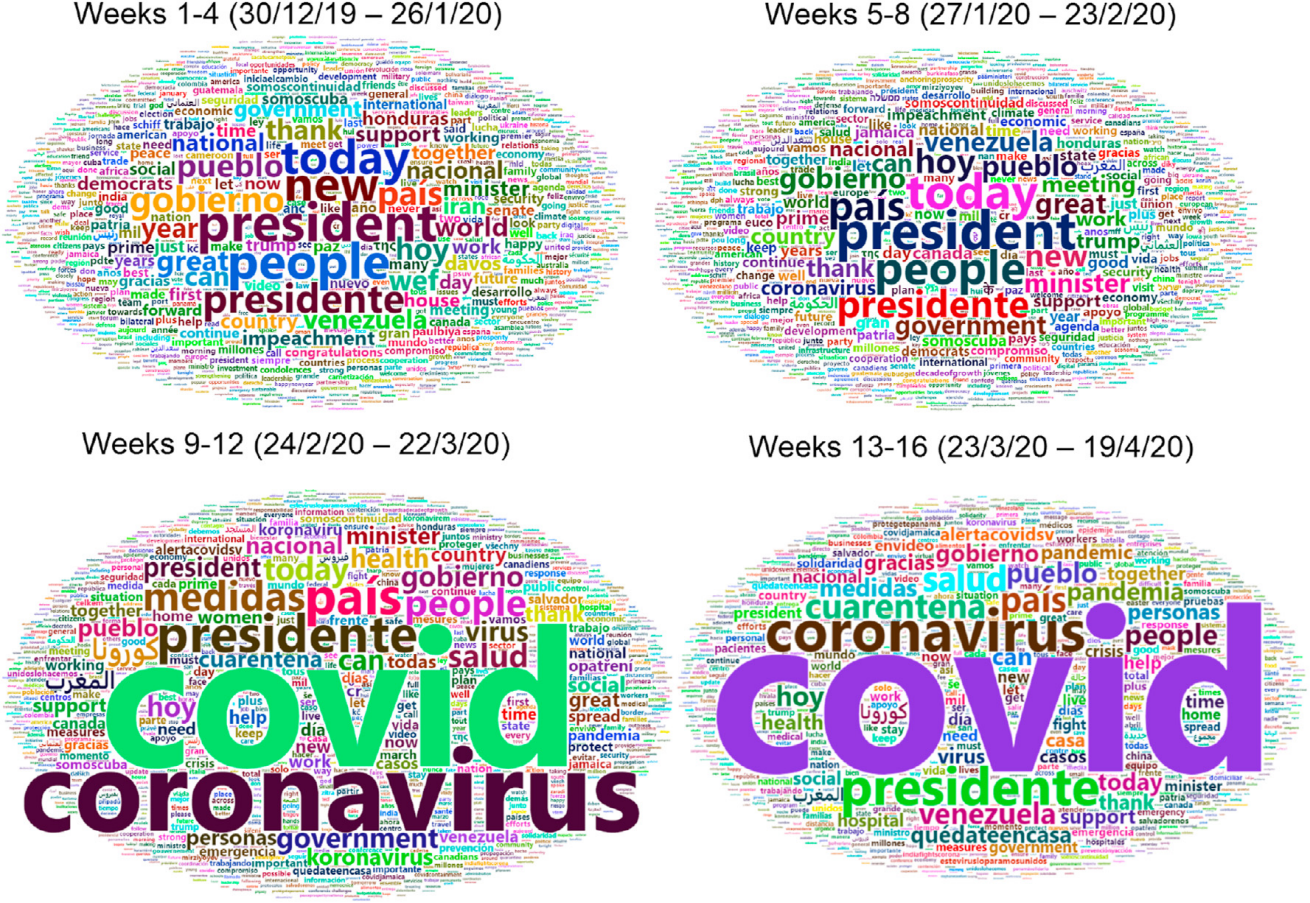
Word clouds.

The second insight from social media analytics relates to what words leaders used. While it is possible to express the content of tweets by certain groups on Twitter in different ways, one of the most effective ways is to visualize it as word clouds. It is a commonly used technique in social media analytics (Grover et al., 2020; Kar, 2020). This has been done in Figure 2. Figure 2 shows the words most frequently used in 16 weeks in four word clouds. The text of each tweet was processed to filter out stop words by using R package stopwords (Benoit et al., 2020). The source of stop words was either “snowball” or “stopwords-iso” depending on the availability of stop words for languages. Tweets, including deleting numbers, were processed to get the words. Also, words were converted to lowercase. In the tweets, the most common language was English followed by Spanish. Therefore, words from these languages dominate. These four word clouds provide visual observation. The word cloud of weeks 1–4 shows that there was no mention of COVID-19. The disease had only just started spreading globally at this point. As Table 1 shows, it was not until the end of January that politicians began to inform people about it. The word cloud of weeks 5–8 indicates that the word “coronavirus” was used^8^. However, it was still not significantly discussed among politicians at that time. The terms “covid” and “coronavirus” dominate weeks 9–12 and 13–16. In weeks 13–16 “covid” was used more often than “coronavirus” in comparison to previous weeks. Other words that were frequently used are associated with the measures put in place against coronavirus such as social distancing, quarantine, and the equivalent words in languages other than English. When March and April are compared to January and even February, it is possible to see how much the discourse on Twitter changed. Therefore, state leaders communicated about the same topic with the same or similar words. There has probably not been many events where one word or term, in this case, “covid”, was used across the globe at the same time by state leaders.

**Figure 3:**
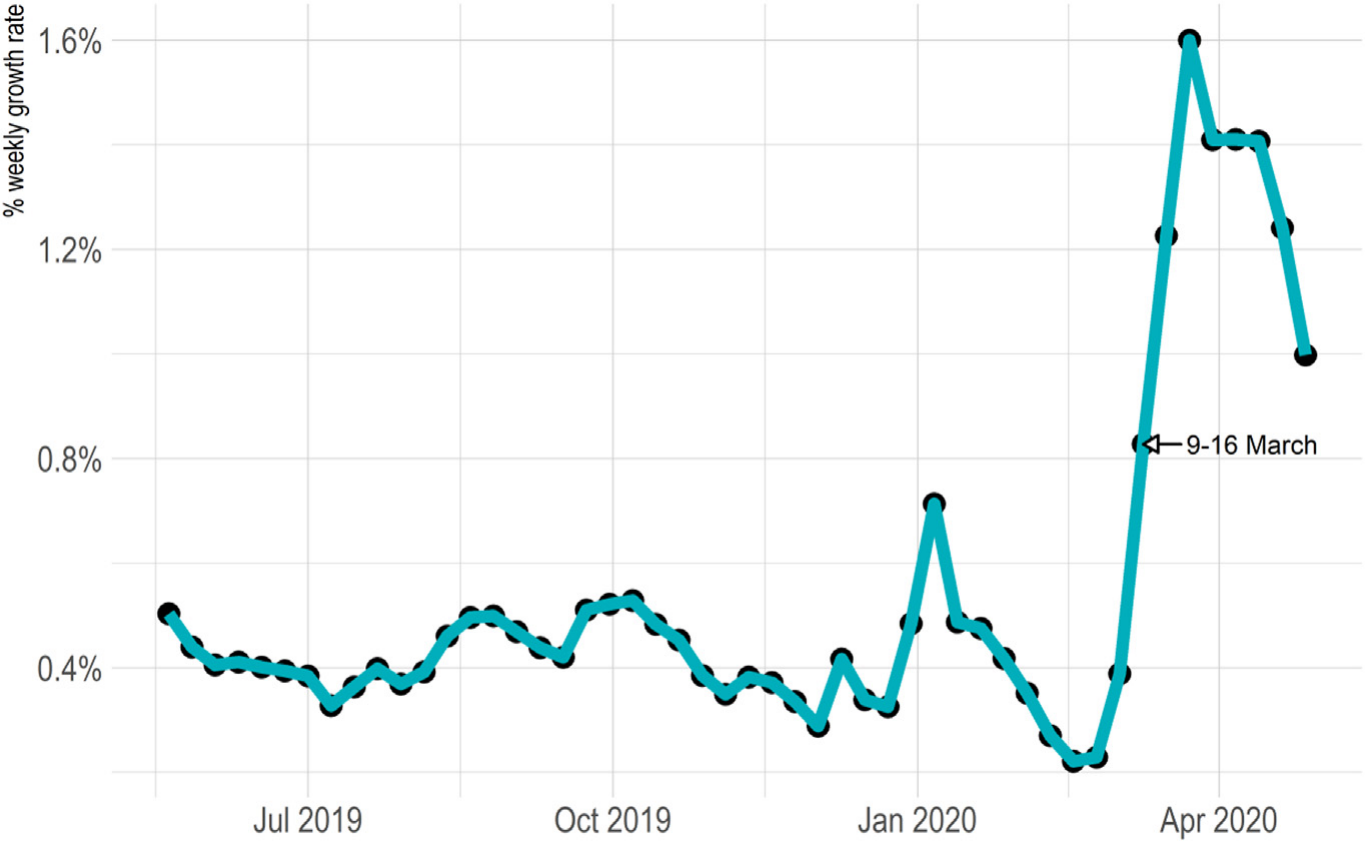
The growth of Twitter followers on leaders' accounts.

Third insight from social media analytics concerns with how often state leaders tweeted. The fourth insight from social media analytics relates to the growth of Twitter followers. First, the number of tweets sent by state leaders was analyzed. Second, the number of followers that state leaders gained on Twitter was examined. One may assume that these matters are connected. Therefore, the next section analyzes them in regression models. However, this section describes and visualizes them. Figure 3 shows the median value of the weekly growth of state leaders. In the case of a change of government, a period for politicians before taking office and two months after taking office was filtered out to mitigate its impact on the growth. Furthermore, as already mentioned, a complete dataset of all weeks for all politicians was not available. However, the lowest number of cases was 110 (the beginning of the timeline) and the highest was 134 (the end of the timeline). Figure 3 clearly shows that the general weekly growth rate was around 0.5% or less. After 9 March 2020, there was a significant increase in the weekly growth rate. In the following weeks, the growth rate was around 1.5%. In this period, there was a significant increase in the number cases worldwide across continents. At the end of April, the weekly growth rate decreased because most countries' measures against the spread of COVID-19 were already put in place by then.

**Figure 4:**
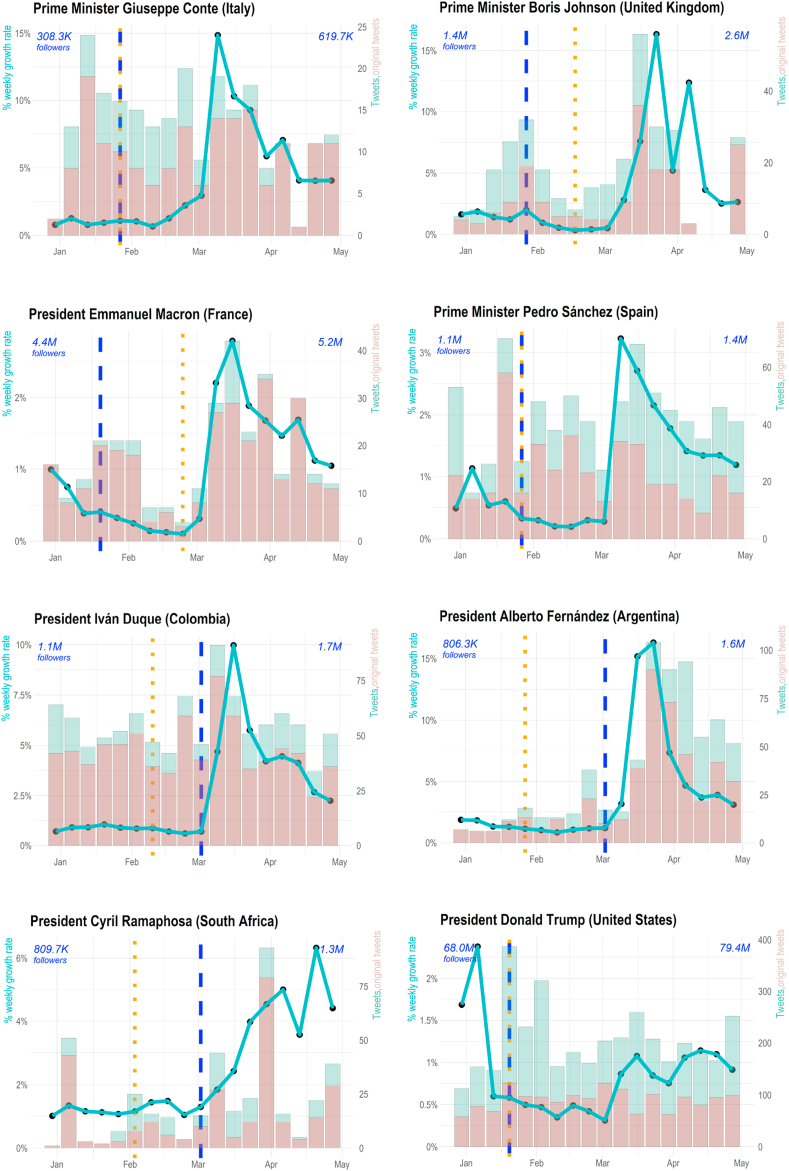
Graphs with eight state leaders. Notes: The blue dashed line indicates the first case of COVID-19 in a country while the dotted orange line denotes the first mention of COVID-19 on Twitter by a particular leader; other figures of state leaders are included as supplementary content.

The growth rate and frequency of tweeting were not equal, and there are significant differences between state leaders. Figure 4 visualizes several points and presents 8 graphs of selected state leaders who are important regional or world leaders and who actively use Twitter. However, the figures of 108 state leaders are available in the supplementary material. For each leader, the growth rate in percentage is the first y-axis in the form of a line, and the number of tweets and retweets is the second y-axis in the form of a histogram. The light blue corresponds with retweets and the pink with original tweets. There is a difference between the usage of Twitter. While some users may use it for original content, others may use it mainly as a tool for spreading news and tweets from external sources and users.

Moreover, there are two vertical lines. The blue dashed line indicates the first case of COVID-19 in a country, while the dotted orange line denotes the first mention of COVID-19 on Twitter by a particular leader. These two lines are important to visualize as one may expect that the growth rate would start after the first mention of COVID-19 and the first confirmed case in a country. Each graph starts with the first week of January (30 December 2019) and the last week ends on 3 May 2020. Therefore, it covers a period of a little over four months. The weeks of January can be considered as a baseline for growth. It is possible to see that there is a sharp increase in the growth rate for all presented leaders in February and especially March. In April, the growth rate declines as the room for growth was partially exhausted. It is more likely that citizens would start following their leaders in February and especially March as the pandemic started to significantly spread. As graphs clearly show, European politicians such as Giuseppe Conte and Boris Johnson even gained around 15% of followers within one week. Similar growth was also experienced by Latin American leaders Iván Duque and Alberto Fernández. Emmanuel Macron and Pedro Sánchez had a lower increase that could have been caused by an already significant number of followers. Interestingly, in the case of Cyril Ramaphosa, the growth rate continued to increase until April. This may be because South Africa, unlike European countries, experienced a significant spread in COVID-19 later. Donald Trump had a higher growth rate at the beginning of the year in the middle of impeachment than later during the COVID-19 pandemic.

### 3.2 Insights from regression models

Descriptive statistics are presented in Table 2 and the results of regression models are presented in Table 3. Model 1 uses all tweets, including retweets, while Model 2 uses only original tweets. However, overall, models show the same results. Models show that state leaders that tweeted more often gained more followers. Citizens want to have new updates about the status of the pandemic. Therefore, it is possible that people who did not use Twitter previously created accounts to start following their state leaders if they believed that they would receive frequent updates in this way. Another explanation could be that citizens may have already been Twitter users but not have followed their state leaders, as they would not see value in that. However, during the COVID-19 pandemic when state leaders have been tweeting frequently, citizens could be persuaded to follow their state leader. Another significant variable is the ratio of the number of followers to internet users. This finding relates to the previous possible explanation. The possibility for the growth of Twitter followers is limited when already leaders are followed by a significant amount of people that have access to the internet. Therefore, in many countries where Twitter was not widely used, the citizens might see it as a new information source and a way of gaining quick access to news.

**Table 2:** Descriptive statistics.

Statistic	N	Mean	St. Dev.	Min	Max
The growth of followers (log)	113	2.833	0.989	0.000	5.660
Tweets (log)	113	4.726	1.478	1.099	8.444
Original tweets (log)	113	4.478	1.354	1.099	7.284
Number of followers to internet users (log)	113	1.466	1.440	-2.590	4.783
Number of cases per one million people (log)	113	5.056	2.232	-0.523	8.714

**Table 3:** Ordinary least-squares (OLS) models.

	Dependent variable	
	The growth of followers (log)	
	(1)	(2)
Tweets (log)	0.162∗∗ (0.051)	
Original tweets (log)		0.169∗∗ (0.055)
Number of followers to internet users (log)	-0.411∗∗∗ (0.050)	-0.408∗∗∗ (0.050)
Number of cases per one million people (log)	-0.053 (0.032)	-0.053 (0.032)

Languages (reference category “other languages”)		
Arabic	0.301 (0.274)	0.306 (0.275)
English	0.377∗ (0.175)	0.366∗ (0.176)
Spanish	0.465 (0.237)	0.491∗ (0.236)
French	1.009∗∗∗ (0.244)	0.992∗∗∗ (0.245)
Constant	2.591∗∗∗ (0.317)	2.598∗∗∗ (0.321)
Observations	113	113
R^2^	0.485	0.482

*Note:*∗p < 0.05; ∗∗p < 0.01; ∗∗∗p < 0.001.

The number of cases per one million people has a negative association with the dependent variable and does not reach statistical significance in the model. One might consider this surprising. However, even countries with a small number of cases have introduced strict measures to prevent the spread of COVID-19. Therefore, the need for information from state leaders may be similar for citizens from countries with a low and high number of cases. The last variable was language. Models show that when country leaders used French their growth rate increased significantly in comparison to the reference group (“other languages”). Many neighboring African countries use French as their main language, and, therefore, there was a possibility to follow various leaders in the region. All other global languages also have a positive direction, and English and Spanish also reach statistical significance. However, their coefficients are lower compared to French.

## 4 Discussion

### 4.1 Contributions to theory and literature

This paper contributes to the significant body of literature examining the COVID-19 pandemic. The study showed that the global pandemic COVID-19 significantly impacted the discourse on Twitter accounts of state leaders. It was the most important topic at that time. Twitter is an important tool for state leaders as it allows them to easily communicate directly with their citizens. They recognize this importance. Therefore, it is not a surprise that most governments have an active presence on social media (Barberá and Zeitzoff, 2018). Findings support that state leaders had an active role on Twitter also during the COVID-19 pandemic. Increased news consumption by people is associated with COVID-19 pandemic (Casero-Ripolles, 2020). Understandably, people want to have information about the health situation in their countries. Twitter is a medium through which news and information can be obtained. Therefore, under the assumption that state leaders provide information on their Twitter accounts, information-seeking citizens should be interested in it. In the analysis of why people follow political actors, Parmelee and Bichard (2012) show that one of the motives for using Twitter is information-seeking. Other studies concluded that for some people information-seeking is the primary motive for using Twitter (Hughes et al., 2012; Johnson and Yang, 2009). During the health emergency that contributes to increased news consumption, mentioned motives should affect Twitter followers count for accounts that provide health and other relevant information during times of crisis. Indeed, findings of this study confirm that most state leaders provide information about the COVID-19 pandemic and their number of Twitter followers greatly increased during the pandemic.

These findings correspond to the UGT (Katz et al., 1973) that states that people want to seek out specific media to fulfill their concrete needs. The UGT is commonly used with social media (Chen, 2011; Hollenbaugh and Ferris, 2014; Hunt et al., 2012; Johnson and Yang, 2009; Pittman and Reich, 2016; Sheldon, 2008; Sheldon and Bryant, 2016; Smock et al., 2011). This approach explains the growth of followers. Some people did not have a need to have a Twitter account before the pandemic. Others might have a Twitter account, but they did not have a need and reason to follow their state leaders. They could use Twitter for entertainment to follow their favorite celebrities. As mentioned throughout the paper, there are various motives for using Twitter than information-seeking. However, the outbreak of COVID-19 affected the needs of many people and at that moment, the need for many was to be informed about matters related to this health crisis. Some may satisfy this need by watching television or listening to the radio. However, some people also chose Twitter as a medium that can fulfill these needs. Then, this led to significant growth of Twitter followers when the pre-pandemic and pandemic period is compared.

The finding on the growth of followers offers some contrast to the literature on spreading misinformation during the COVID-19 pandemic (Gruzd and Mai, 2020; Lovari, 2020; Pérez-Dasilva et al., 2020; Pulido et al., 2020; Rodríguez et al., 2020; Vraga et al., 2020). It is indeed true that misinformation can be spread via social media. However, state leaders should provide relevant and legitimate information about the health situation. This can potentially lead to better knowledge about the topic and adopting adequate measures to limit the spread of COVID-19. It seems that a significant amount of people might be interested in this information. Therefore, after all, the use of social media during the pandemic may not be as harmful if citizens consume relevant content.

### 4.2 Implications to practice

There are some clear implications to practice from this study. During times of crisis, political actors and relevant authorities should offer relevant information through different media. Twitter, as a social media website, is a relatively new medium compared to traditional television or radio. At the moment, not all state leaders use Twitter. However, this study shows that state leaders who tweeted more frequently gained more Twitter followers. Therefore, people will follow state leaders when they offer information to fulfill their needs during health emergencies. Understandably, there are fewer reasons and less motivation to follow rather inactive accounts. In today's digital age, governments should make use of modern technology, such as Twitter, to be transparent on the state of the country and provide relevant health information for their citizens. Recommendation and information from relevant authorities are important for preventing the spread of COVID-19 as a study on U. S. governors' Twitter communications confirms (Grossman et al., 2020). However, of course, information and government transparency are not connected only with the COVID-19 pandemic. In general, state actors, governments, and their agencies should offer relevant and swift information via Twitter on state issues and matter.

### 4.3 Limitations and future research

The limitation of the study is that it was not feasible to confirm that followers are legitimate accounts of citizens. However, the same limitation also applies to the pre-pandemic period. The study focused only on Twitter follower counts and did not analyze followers. This is understandable as the study would have to analyze the activity of hundreds of millions of Twitter accounts in different time periods. It is very difficult to detect whether accounts are legitimate, especially if accounts are passive and do not tweet.

In relation to this study, there is a possibility for future research. It will be especially interesting to see whether politicians retain newly obtained followers or whether users will unfollow them in large numbers when the crisis is over. It is already possible to see that the growth rate is decreasing in comparison to March 2020 in the case of the majority of politicians. However, it does not seem likely that the crisis will be over soon. Therefore, it is possible that there will not be any mass unfollowing soon and that more people will use Twitter.

## 5 Conclusion

The study showed that the global pandemic COVID-19 significantly impacted the discourse on Twitter. It was the most important topic at that time. Furthermore, most leaders reacted to the spread of COVID-19. While some mentioned COVID-19 already in January, others mentioned it first in February, March, or April. However, in total, 64.8% of UN member states had a leader that tweeted about COVID-19. Another key finding is that there was a significant growth rate of Twitter followers for state leaders in comparison with pre-pandemic months. Leaders had a higher growth of followers when they used their Twitter accounts more frequently. Therefore, it seems that citizens were interested in the latest update from their leading politicians. Furthermore, especially French-speaking politicians had a higher growth rate compared to leaders speaking other languages and the growth rate was higher when the ratio of the number of followers to internet users was lower as there was room for growth.

## Declarations

### Author contribution statement

M. Haman: Conceived and designed the experiments; Performed the experiments; Analyzed and interpreted the data; Contributed reagents, materials, analysis tools or data; Wrote the paper.

### Funding statement

This work was supported by The Philosophical Faculty, University of Hradec Králové, Czech Republic.

### Data availability statement

Data included in article/supplementary material/referenced in article.

### Declaration of interests statement

The authors declare no conflict of interest.

### Additional information

No additional information is available for this paper.

## Appendix A Supplementary data

appendix_figures01_heliyon appendix_figures01_heliyon

appendix_figures02_heliyon appendix_figures02_heliyon

## Footnotes

1 The first calendar week in 2020 began on this date. No leader could inform about COVID-19 before this date as the WHO first tweeted about “a cluster of pneumonia cases” in Wuhan on 4 January 2020.

2 However, in 10 cases, Presidents were also included when Prime Ministers were not active if a President was and if they had more than 30,000 followers. In many parliamentary systems, Presidents are directly elected. Therefore, their role is not insignificant.

3 For regression models, it was sufficient to have information on the growth of followers from 27 January to 3 May 2020.

4 Tweets also include retweets.

5 For the growth of followers, a constant (1.0036) was added to each number before log transformation so the lowest value was positive and a log-transformed number equal 0.

6 Of the languages included in the group “other languages”, Portuguese was the most used by four leaders.

7 The creation date (GMT zone) of Tweets was used from the Twitter API. Therefore, the time zone may not correspond to the leaders' time zones.

8 In the word cloud, the term “coronavirus” is to the left of the word “people”.
