# Supplementary material for: The use of Twitter by state leaders and its impact on the public during the COVID-19 pandemic
Source: Heliyon. 2020 Nov 19;6(11):e05540. doi: 10.1016/j.heliyon.2020.e05540 (PMC7695954; doi:10.1016/j.heliyon.2020.e05540)
Supplement: appendix_figures01_heliyon [file mmc1.pdf]

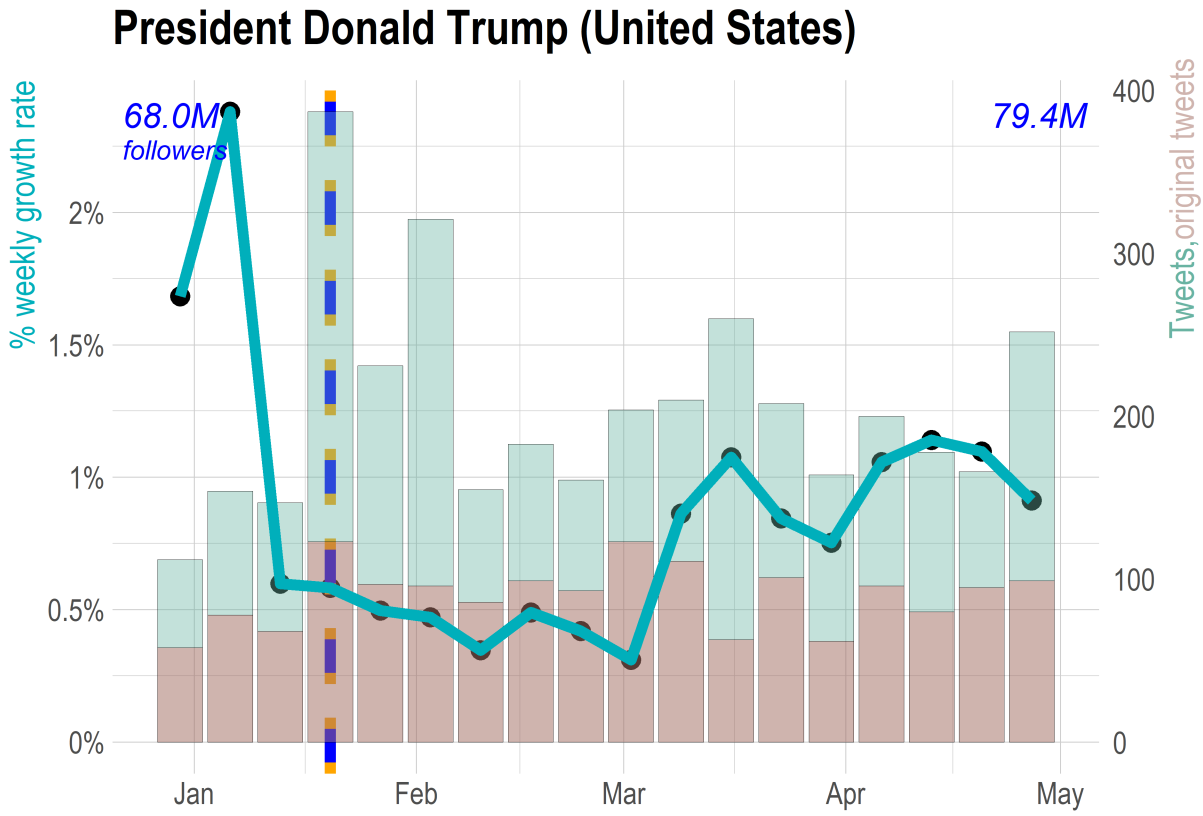

President Donald Trump (United States)
68.0M
followers
79.4M
% weekly growth rate
Tweets, original tweets
0%
0.5%
1%
1.5%
2%
0
100
200
300
400
Jan
Feb
Mar
Apr
May

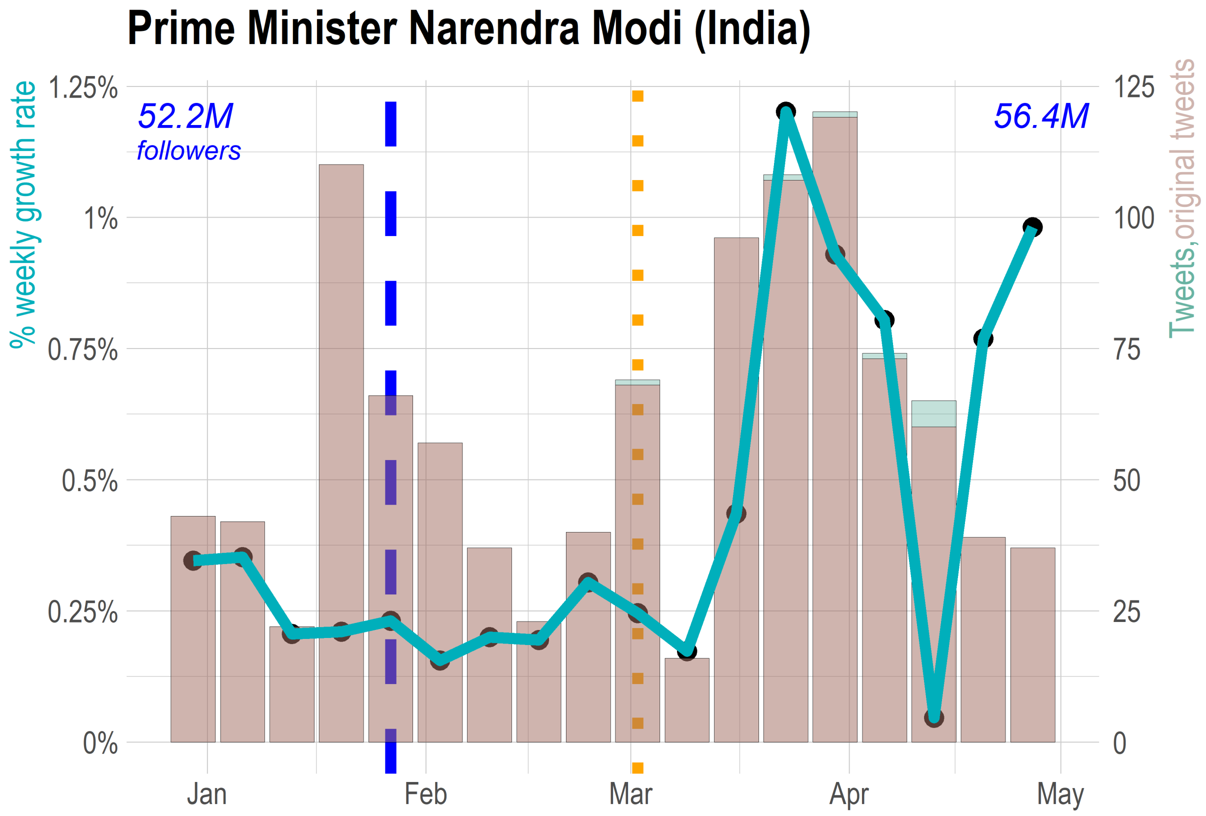

Prime Minister Narendra Modi (India)
% weekly growth rate
Tweets, original tweets
52.2M
followers
56.4M
1.25%
1%
0.75%
0.5%
0.25%
0%
125
100
75
50
25
0
Jan
Feb
Mar
Apr
May

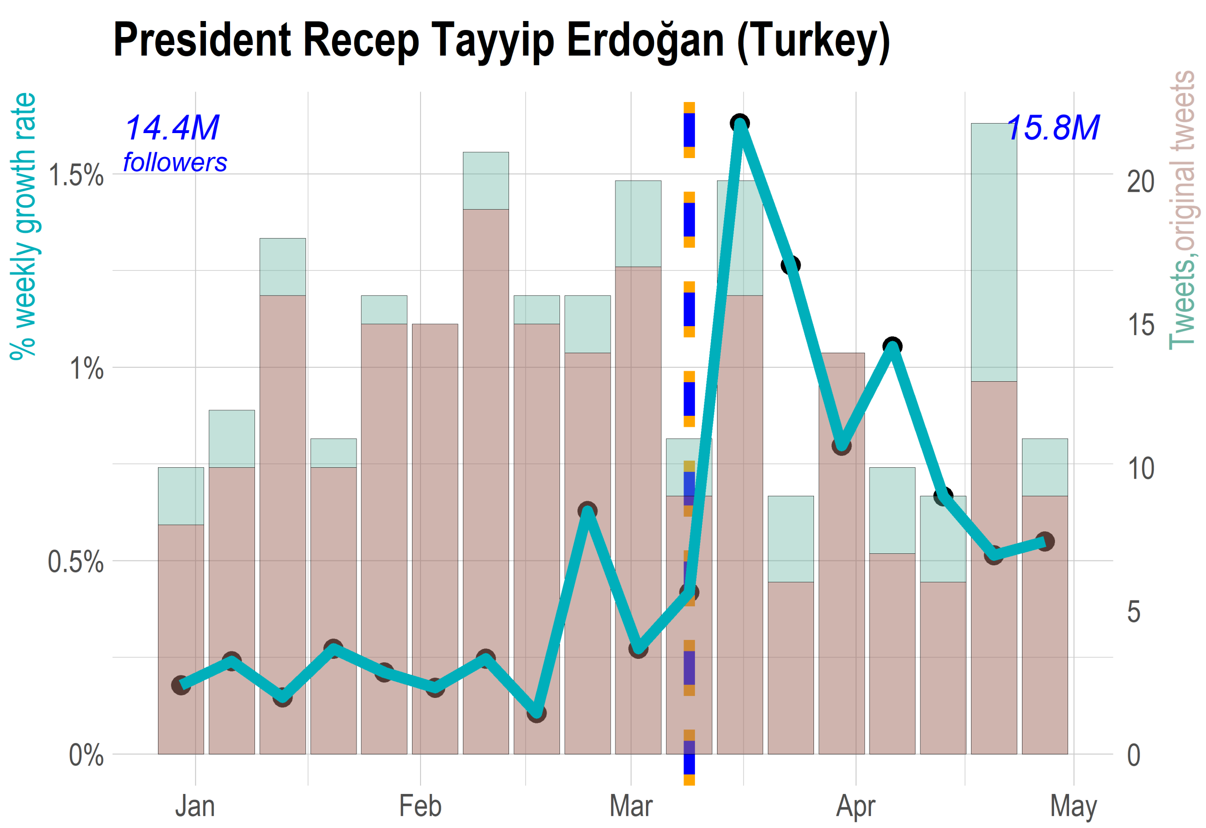
President Recep Tayyip Erdoğan (Turkey)
14.4M
followers
15.8M
% weekly growth rate
Tweets, original tweets
1.5%
1%
0.5%
0%
20
15
10
5
0
Jan
Feb
Mar
Apr
May

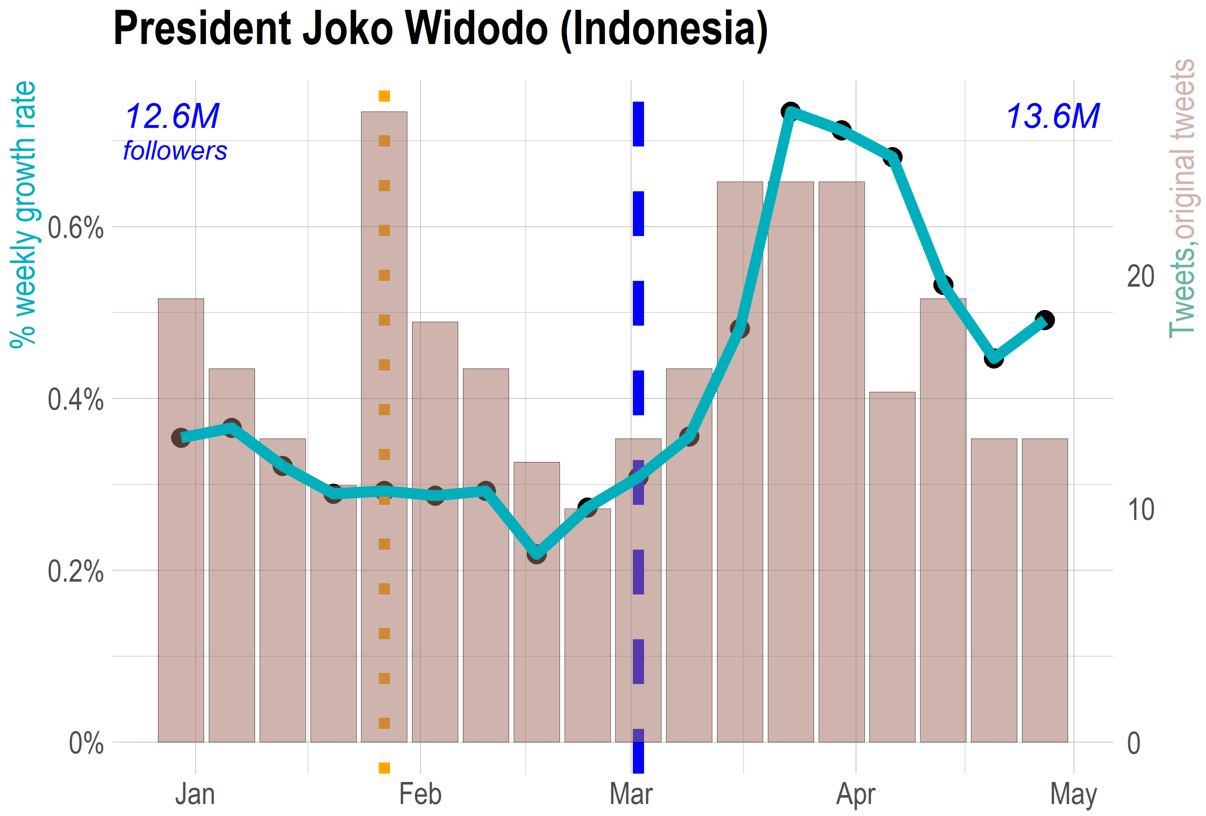

President Joko Widodo (Indonesia)
% weekly growth rate
Tweets, original tweets
12.6M
followers
13.6M
0.6%
0.4%
0.2%
0%
20
10
0
Jan
Feb
Mar
Apr
May

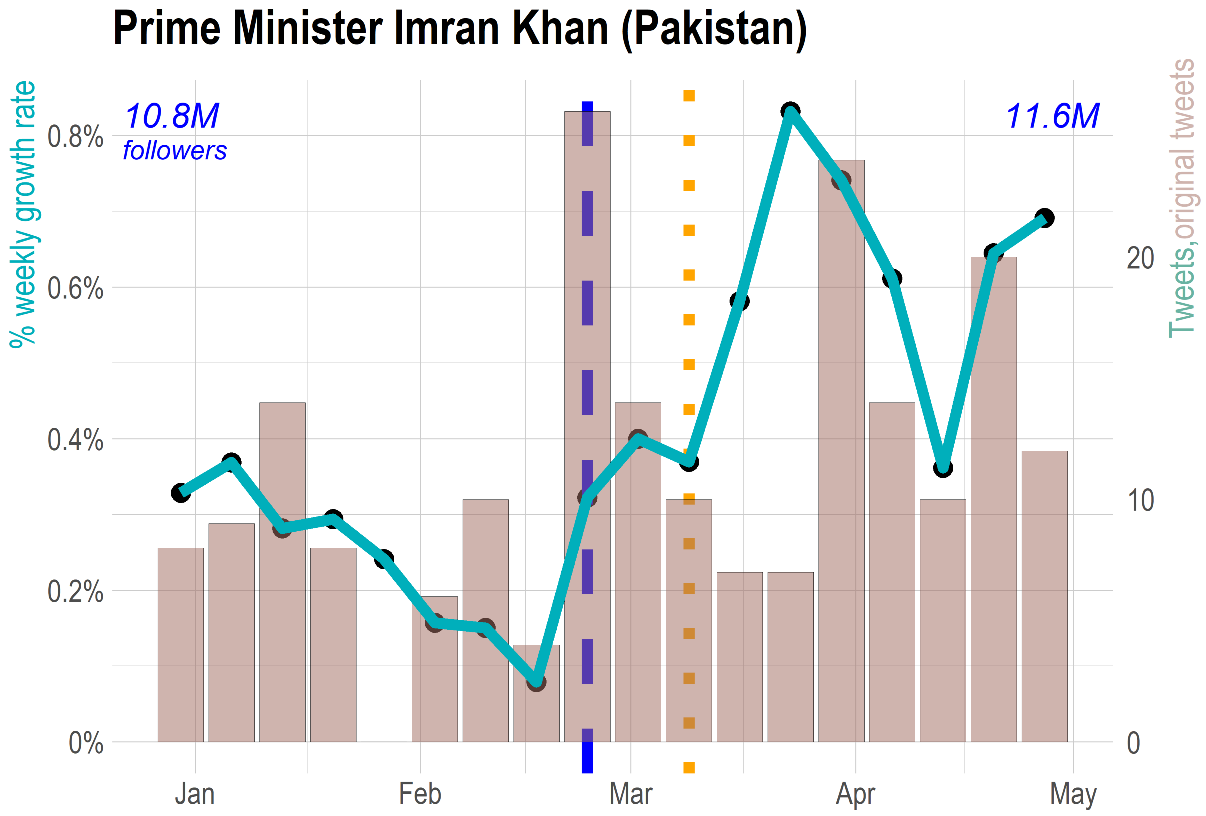

Prime Minister Imran Khan (Pakistan)
10.8M
followers
11.6M
% weekly growth rate
Tweets, original tweets
0.8%
0.6%
0.4%
0.2%
0%
20
10
0
Jan
Feb
Mar
Apr
May

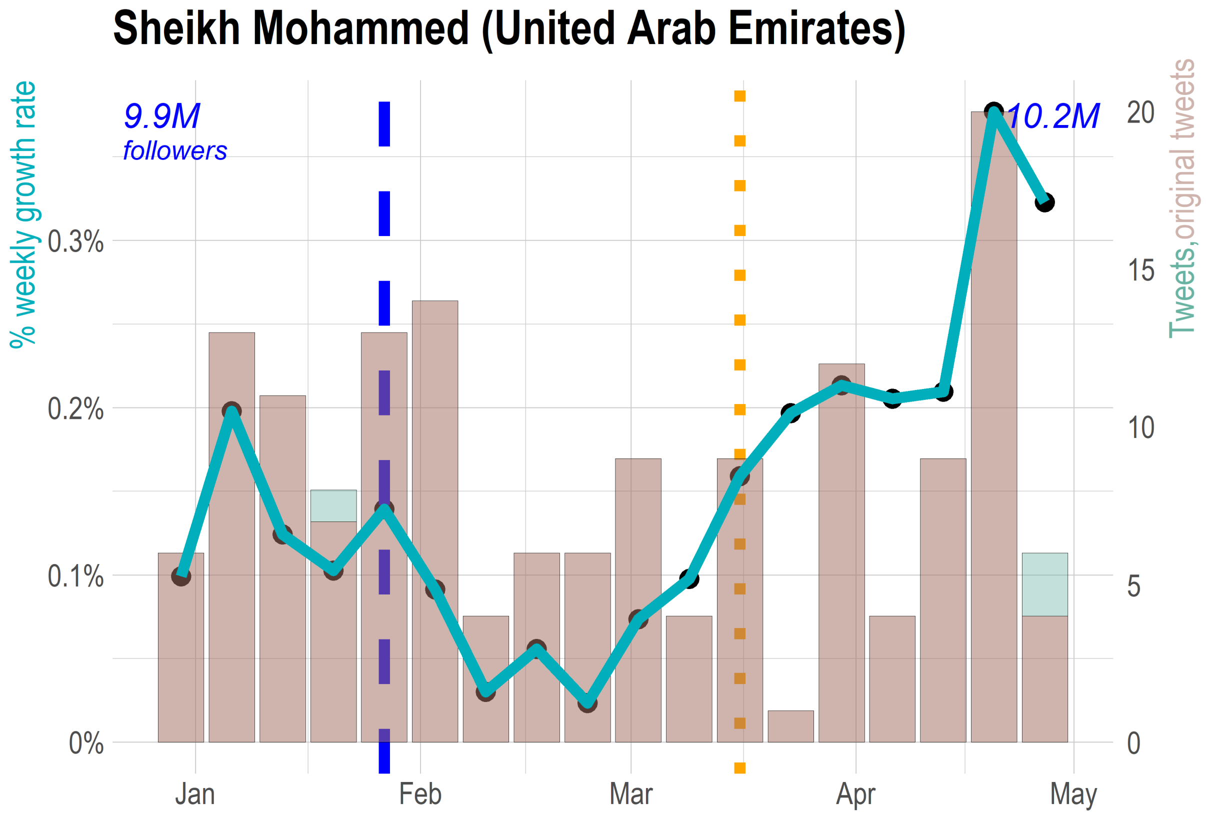
Sheikh Mohammed (United Arab Emirates)
9.9M
followers
10.2M
% weekly growth rate
Tweets, original tweets
0.3%
0.2%
0.1%
0%
20
15
10
5
0
Jan
Feb
Mar
Apr
May

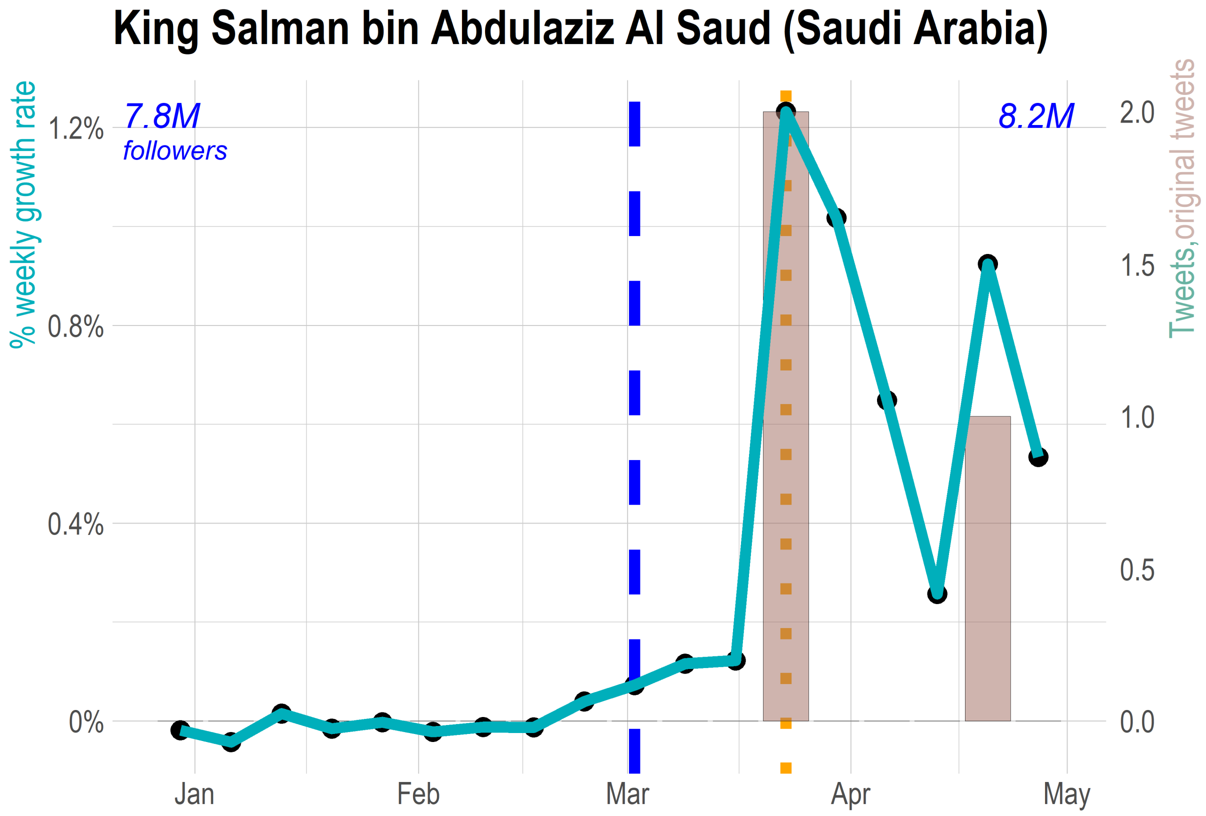
King Salman bin Abdulaziz Al Saud (Saudi Arabia)
% weekly growth rate
Tweets, original tweets
7.8M
followers
8.2M
1.2%
0.8%
0.4%
0%
2.0
1.5
1.0
0.5
0.0
Jan
Feb
Mar
Apr
May

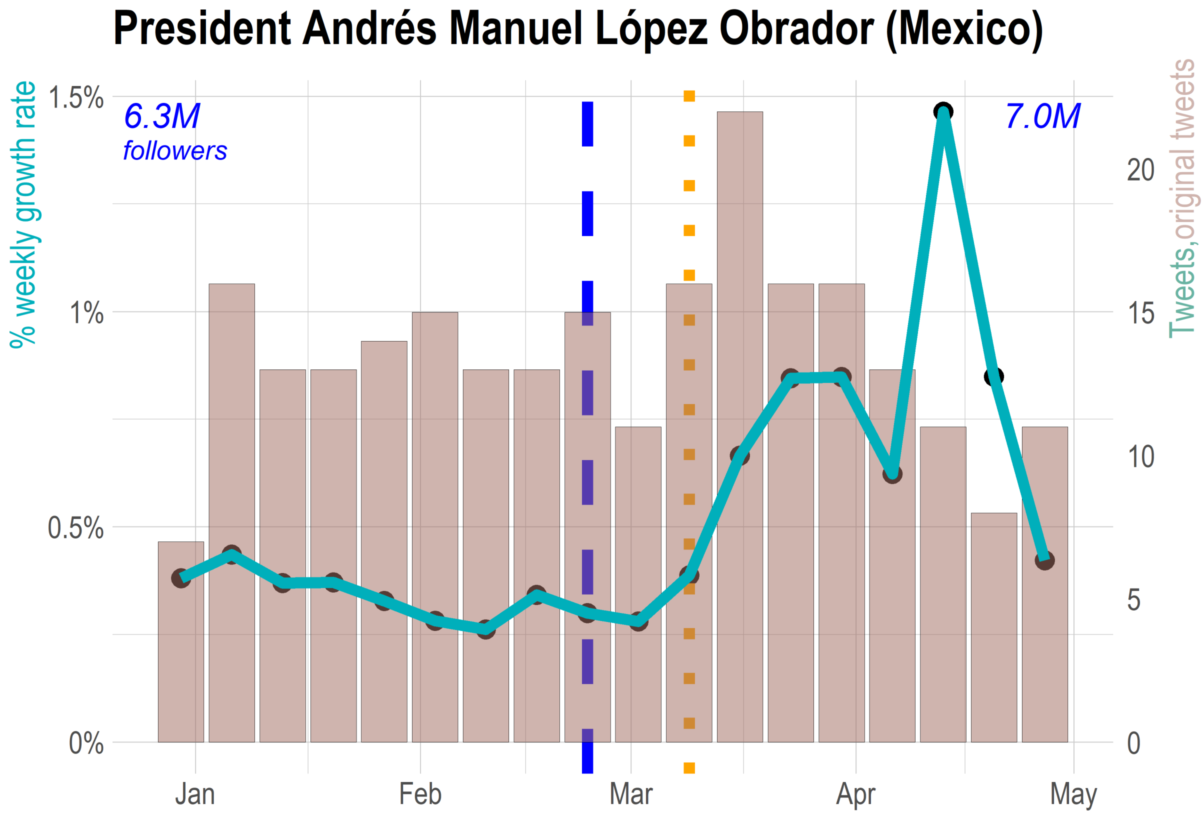

President Andrés Manuel López Obrador (Mexico)
% weekly growth rate
Tweets, original tweets
6.3M
followers
7.0M
1.5%
1%
0.5%
0%
20
15
10
5
0
Jan
Feb
Mar
Apr
May

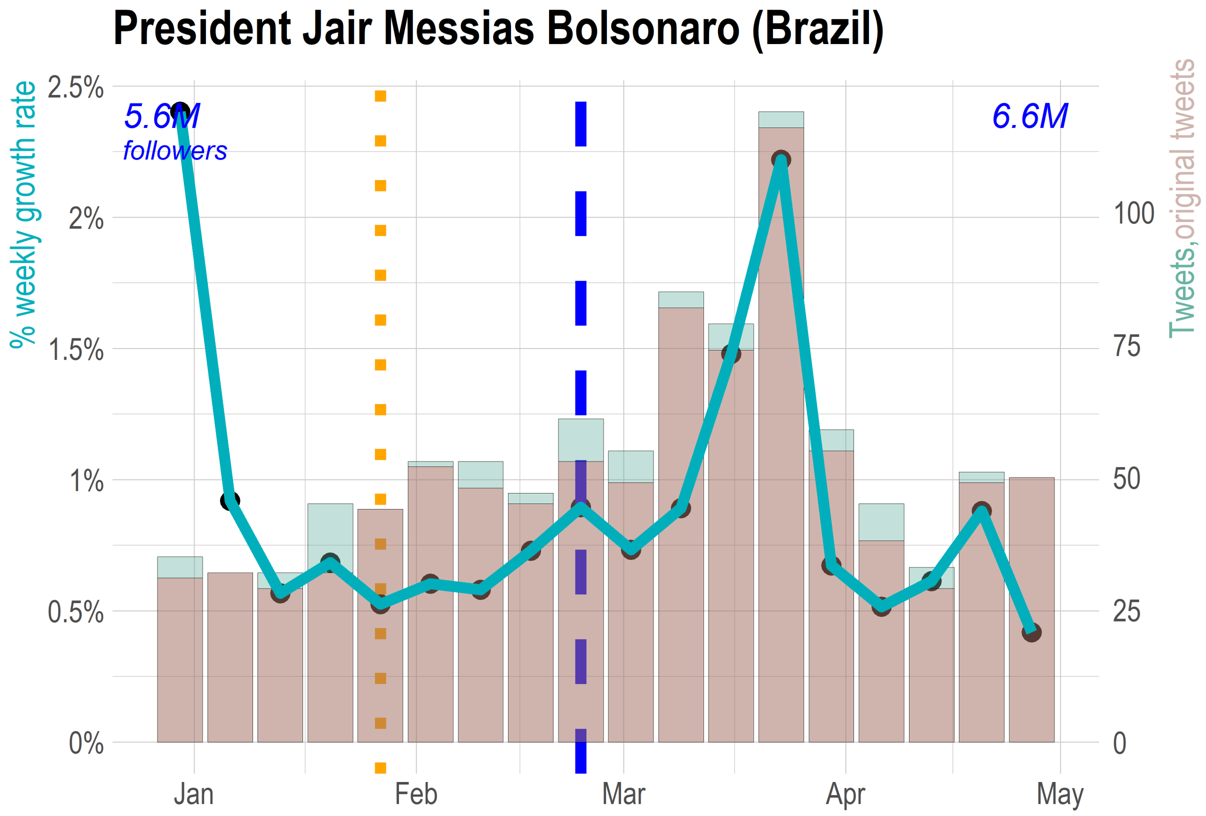

President Jair Messias Bolsonaro (Brazil)
% weekly growth rate
Tweets, original tweets
5.6M
followers
6.6M
2.5%
2%
1.5%
1%
0.5%
0%
100
75
50
25
0
Jan
Feb
Mar
Apr
May

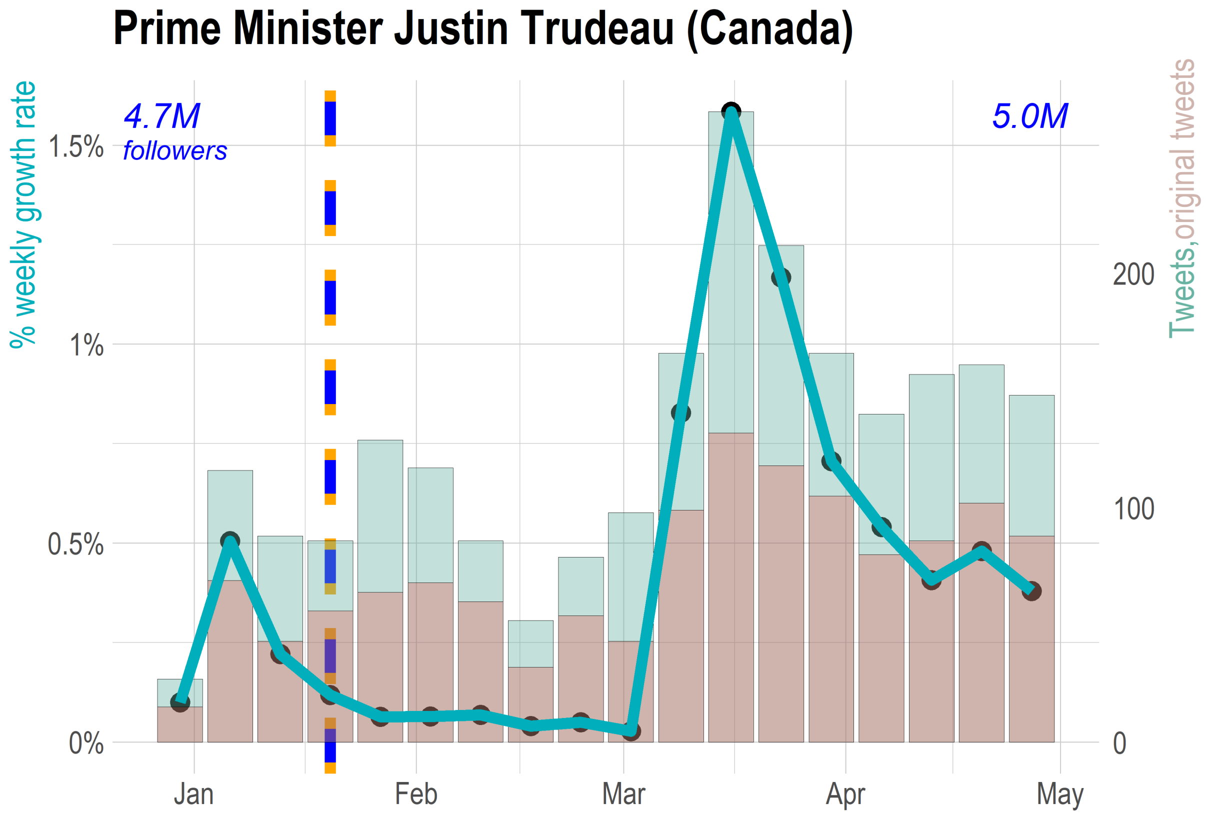

Prime Minister Justin Trudeau (Canada)
% weekly growth rate
Tweets, original tweets
4.7M
followers
5.0M
1.5%
1%
0.5%
0%
200
100
0
Jan
Feb
Mar
Apr
May

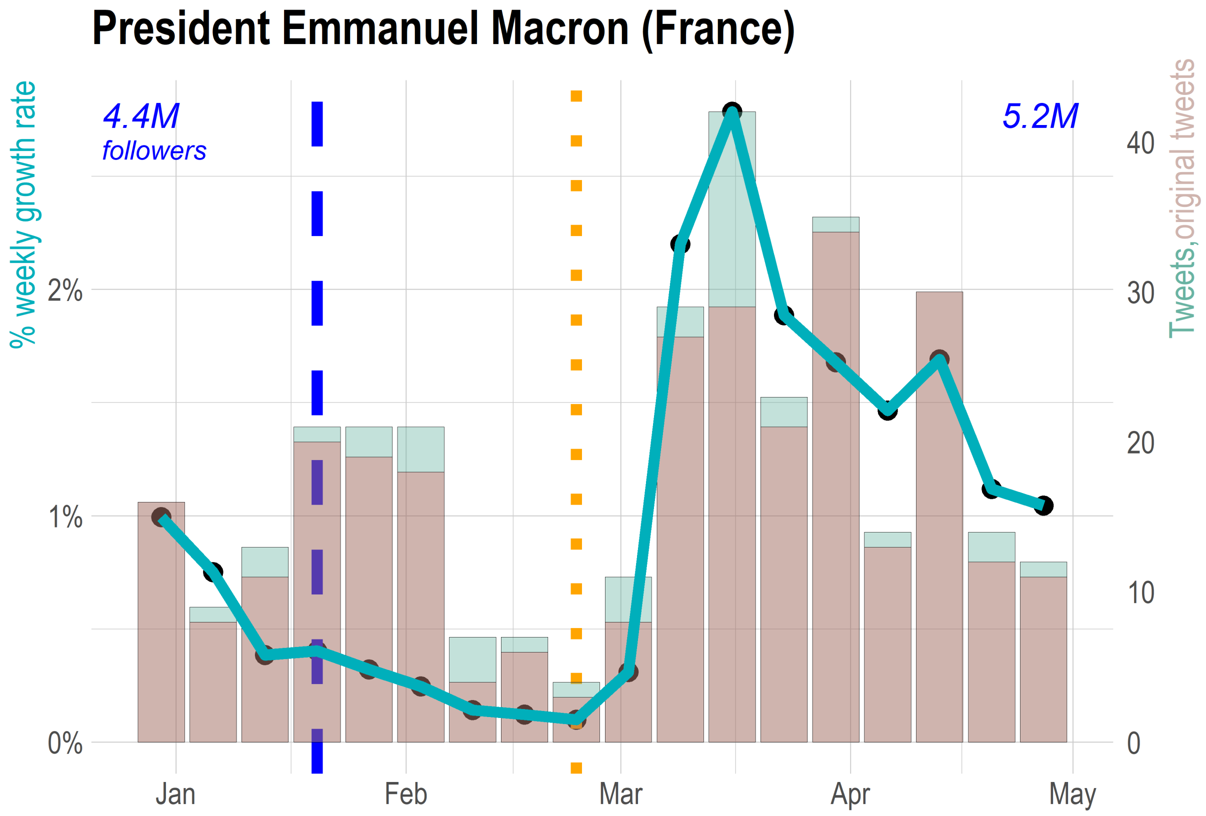

President Emmanuel Macron (France)
% weekly growth rate
Tweets, original tweets
4.4M
followers
5.2M
0%
1%
2%
0
10
20
30
40
Jan
Feb
Mar
Apr
May

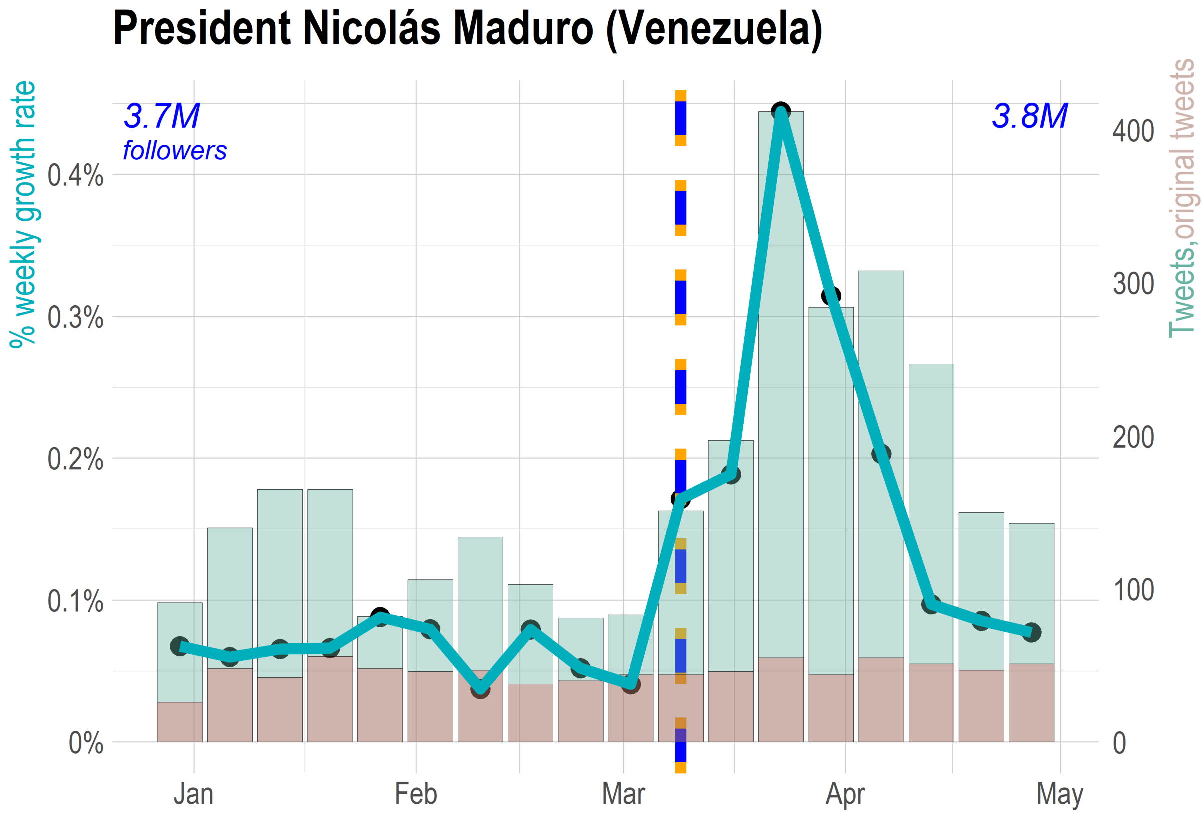

President Nicolás Maduro (Venezuela)
% weekly growth rate
Tweets, original tweets
3.7M
followers
3.8M
0.4%
0.3%
0.2%
0.1%
0%
400
300
200
100
0
Jan
Feb
Mar
Apr
May

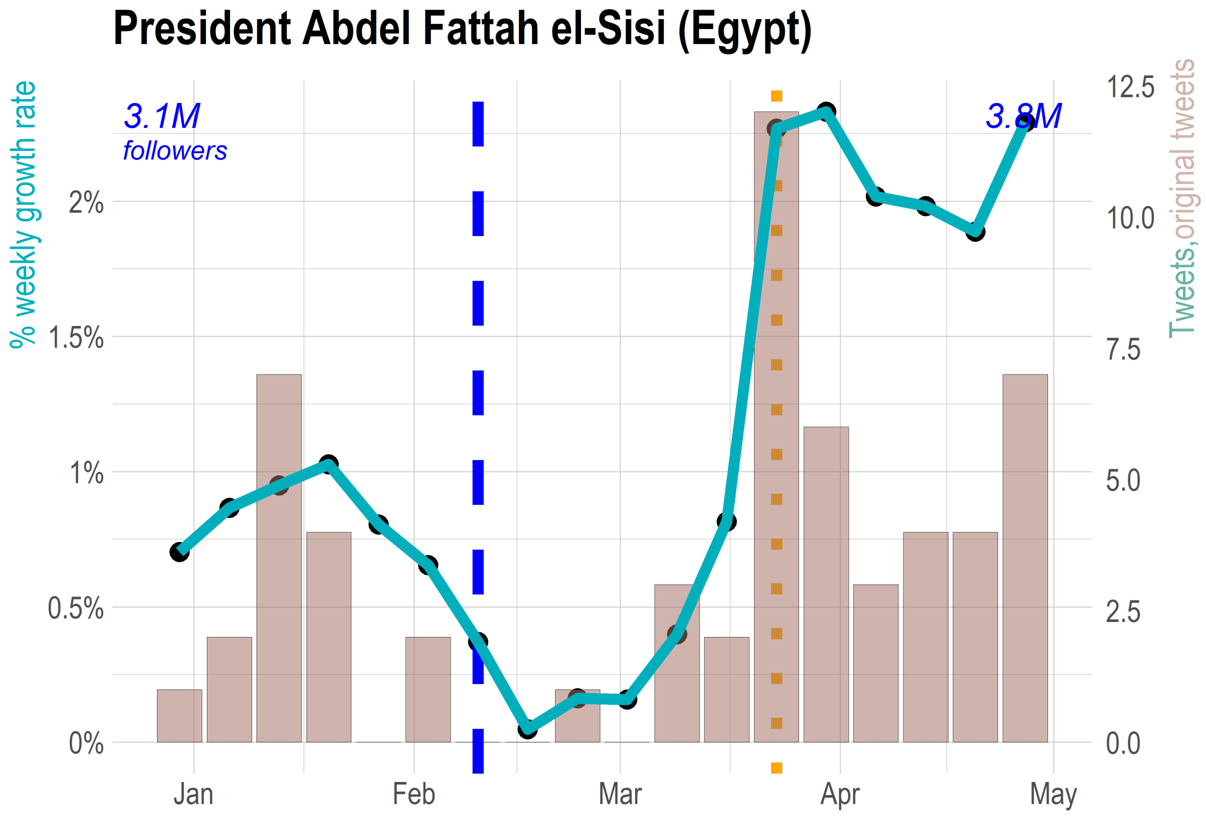
President Abdel Fattah el-Sisi (Egypt)
% weekly growth rate
Tweets, original tweets
3.1M
followers
3.8M
2%
1.5%
1%
0.5%
0%
12.5
10.0
7.5
5.0
2.5
0.0
Jan
Feb
Mar
Apr
May

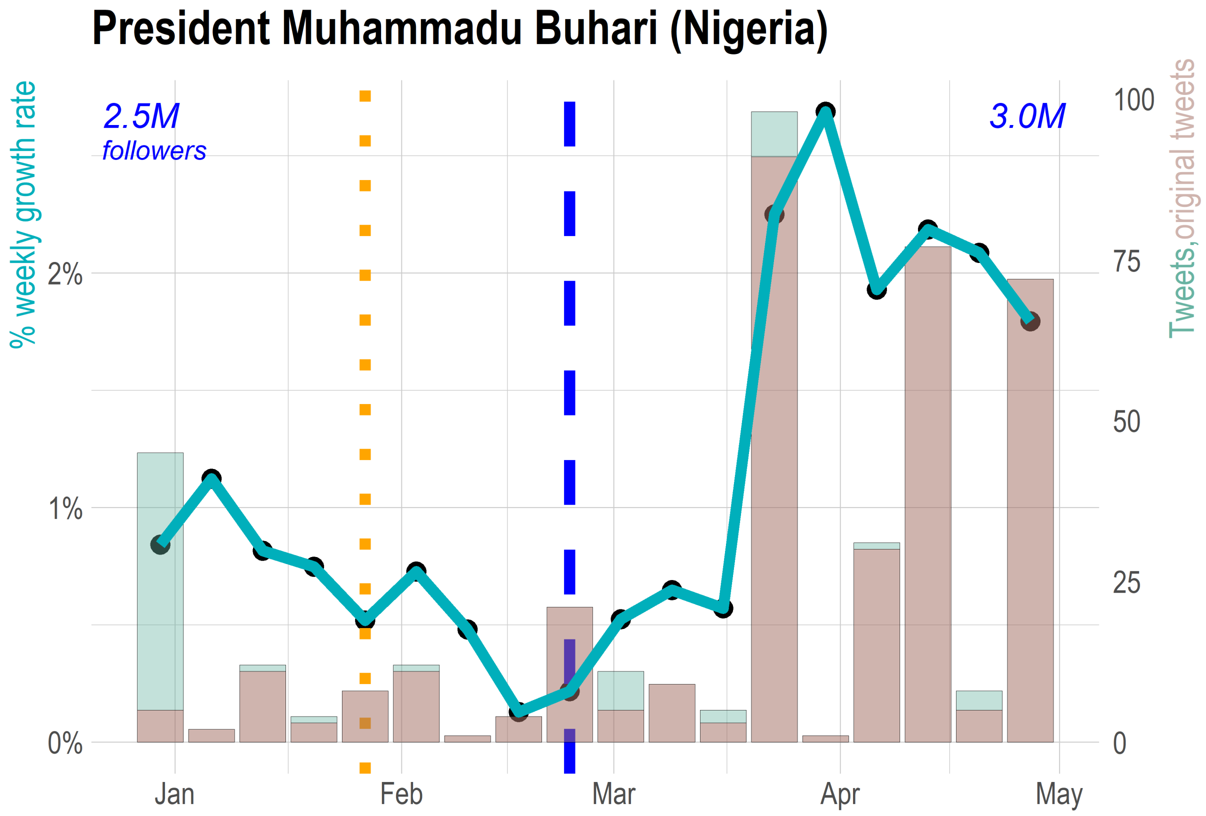

President Muhammadu Buhari (Nigeria)
% weekly growth rate
Tweets, original tweets
2.5M
followers
3.0M
0%
1%
2%
0
25
50
75
100
Jan
Feb
Mar
Apr
May

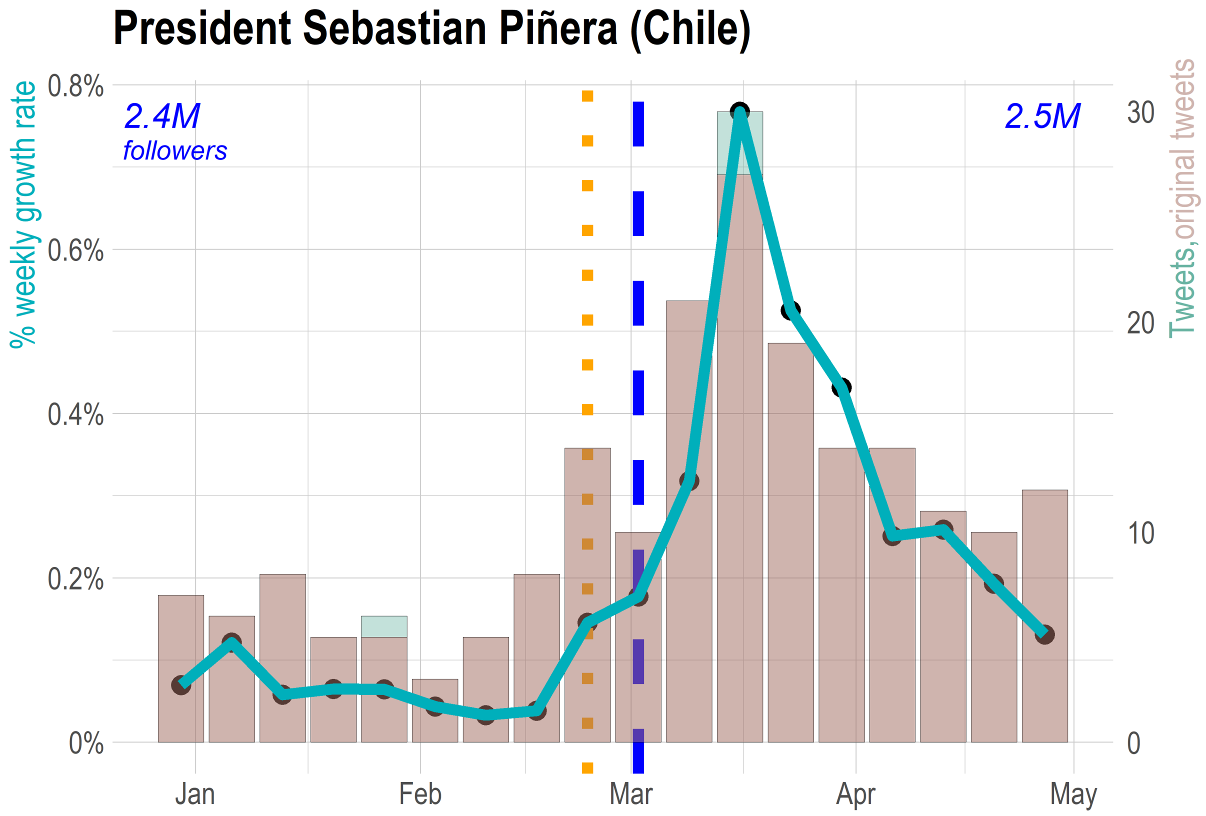
President Sebastian Piñera (Chile)
% weekly growth rate
0.8%
0.6%
0.4%
0.2%
0%
2.4M
followers
2.5M
Tweets, original tweets
30
20
10
0
Jan
Feb
Mar
Apr
May

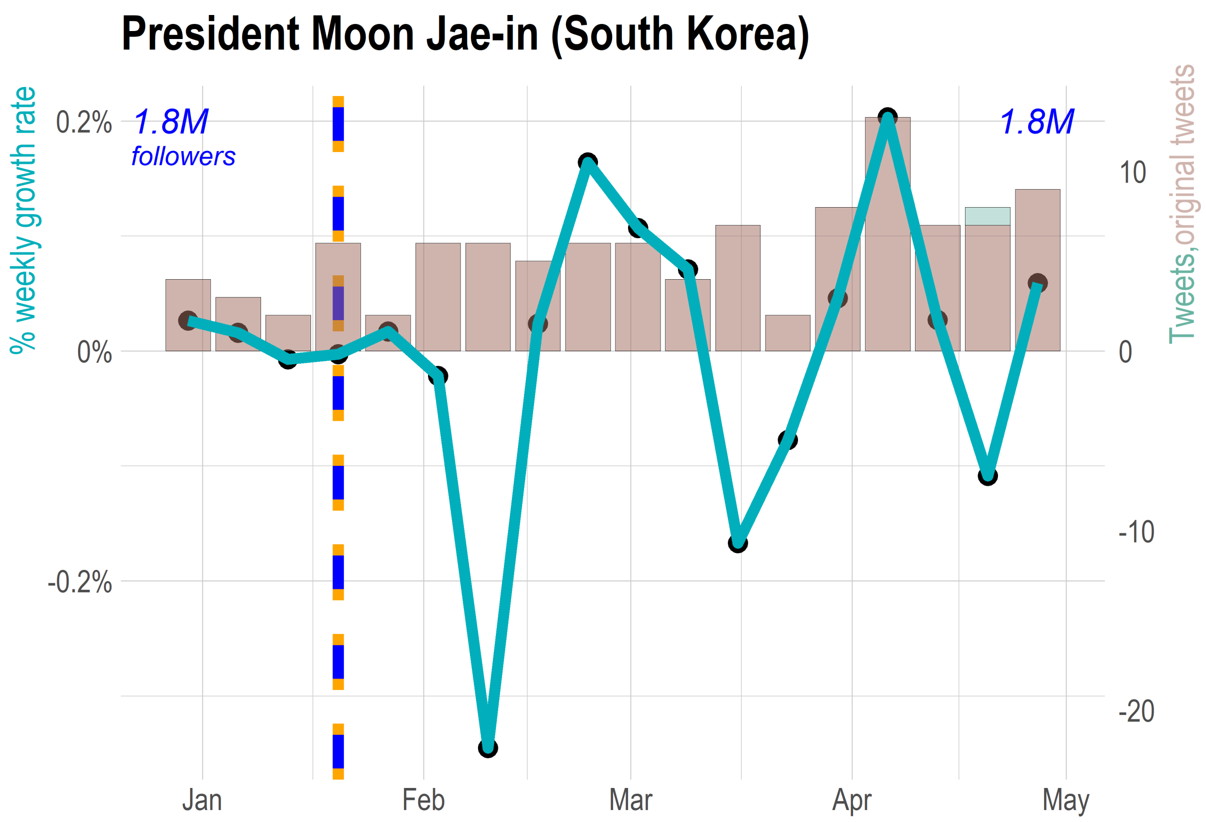

President Moon Jae-in (South Korea)
% weekly growth rate
Tweets, original tweets
1.8M
followers
1.8M
0.2%
0%
-0.2%
10
0
-10
-20
Jan
Feb
Mar
Apr
May

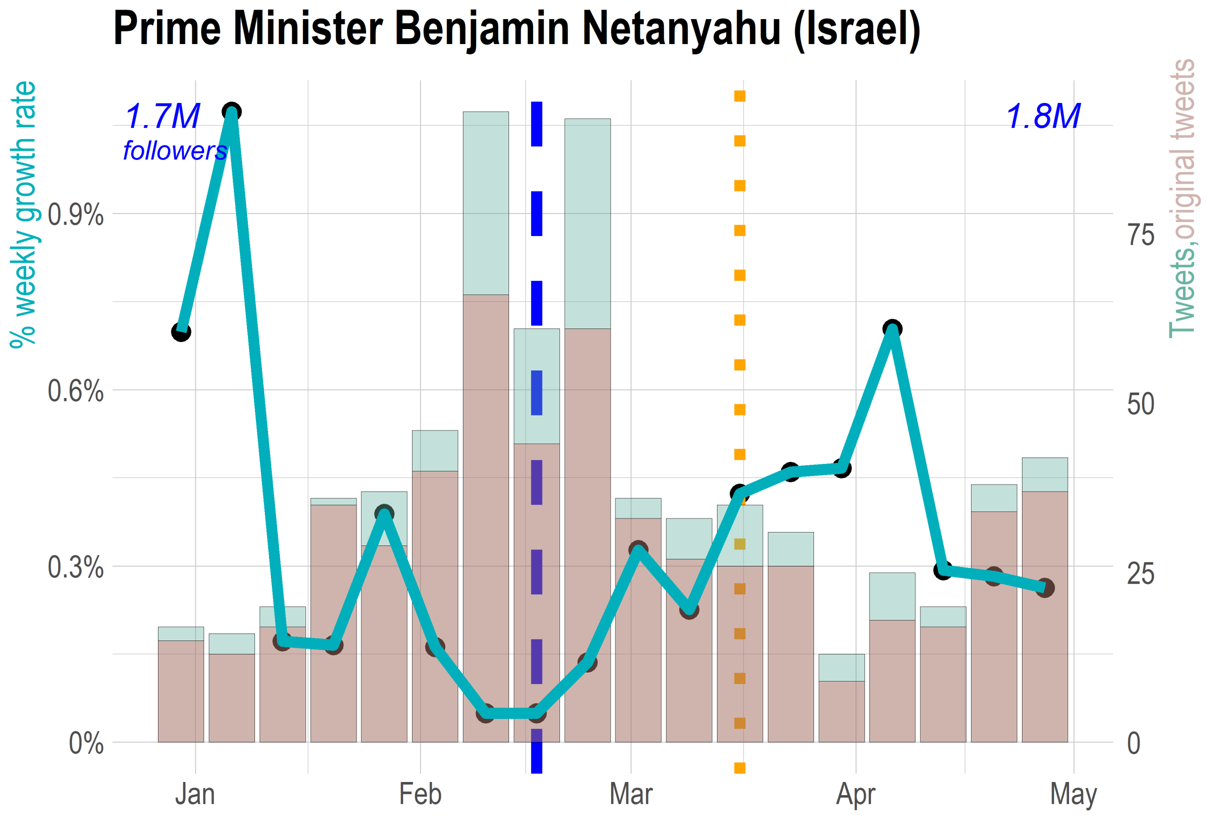
Prime Minister Benjamin Netanyahu (Israel)
% weekly growth rate
Tweets, original tweets
1.7M
followers
1.8M
0.9%
0.6%
0.3%
0%
75
50
25
0
Jan
Feb
Mar
Apr
May

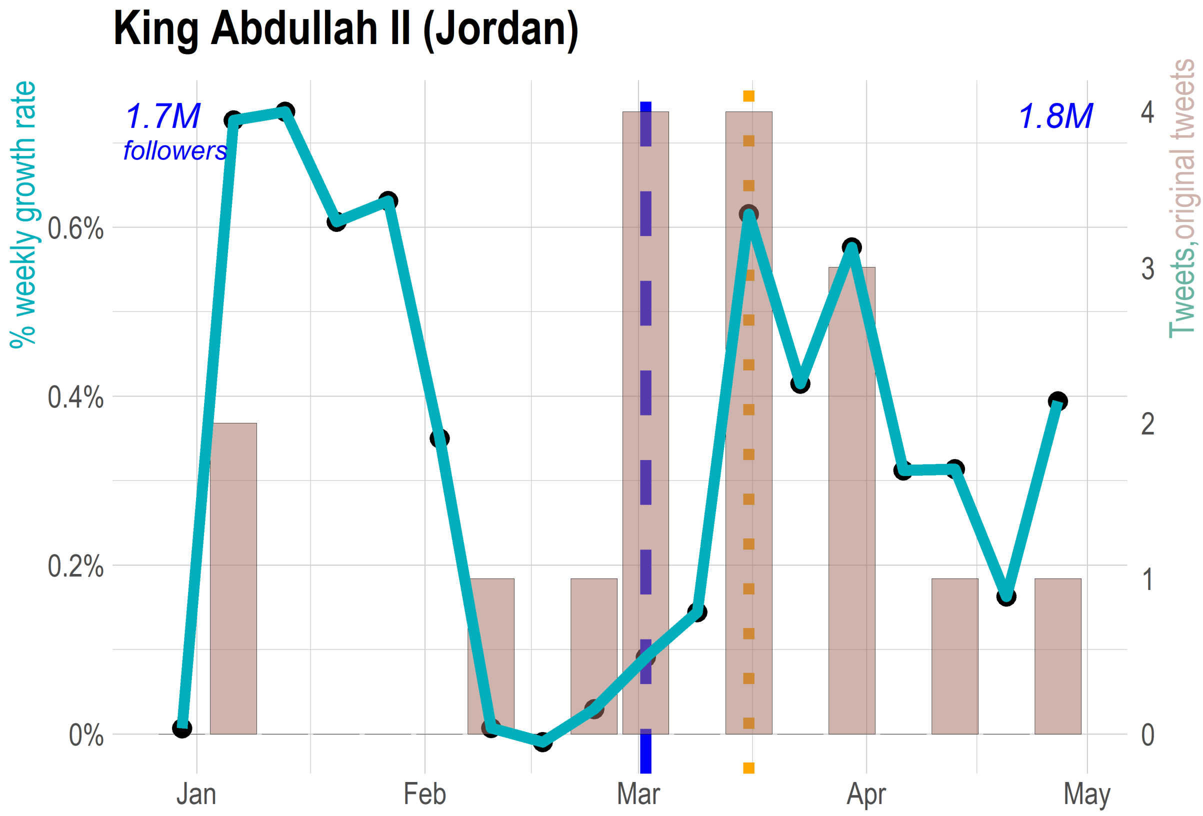

King Abdullah II (Jordan)
% weekly growth rate
Tweets, original tweets
1.7M
followers
1.8M
0.6%
0.4%
0.2%
0%
4
3
2
1
0
Jan
Feb
Mar
Apr
May

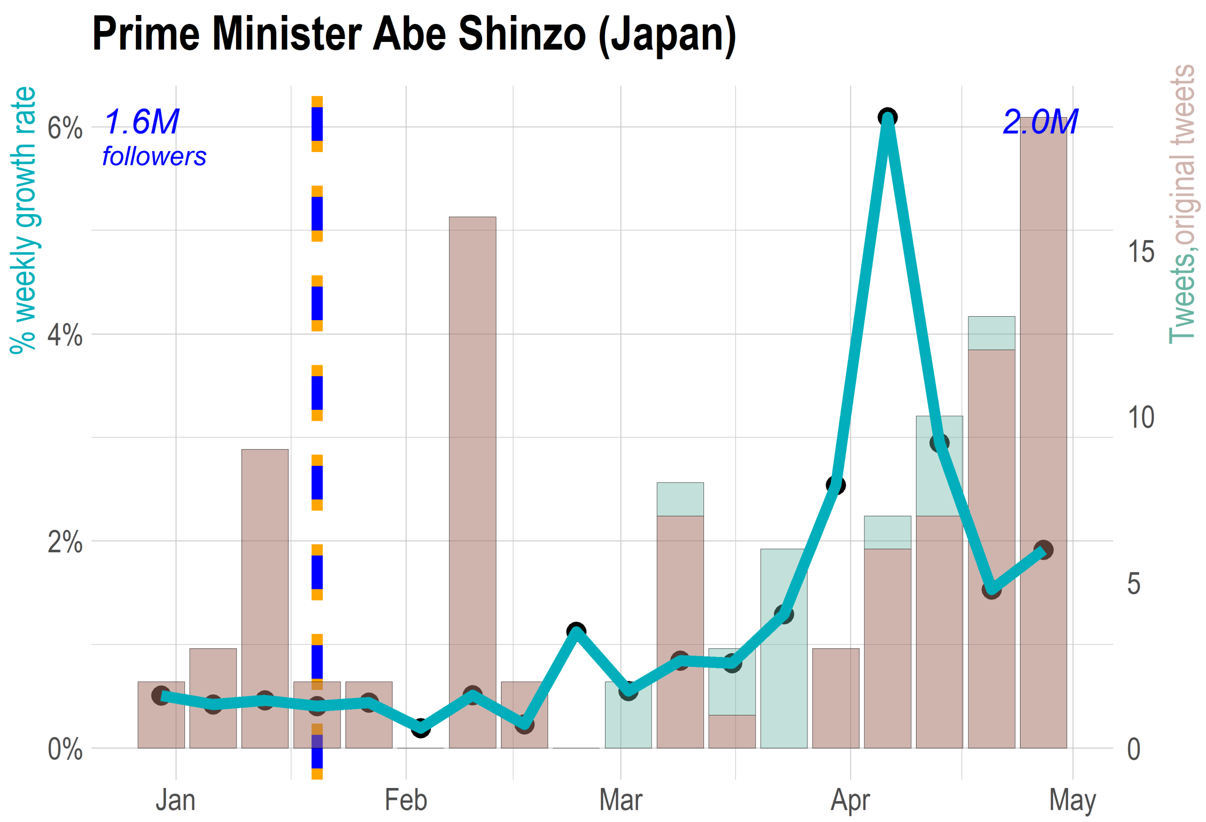

Prime Minister Abe Shinzo (Japan)
% weekly growth rate
Tweets, original tweets
1.6M
followers
2.0M
6%
4%
2%
0%
15
10
5
0
Jan
Feb
Mar
Apr
May

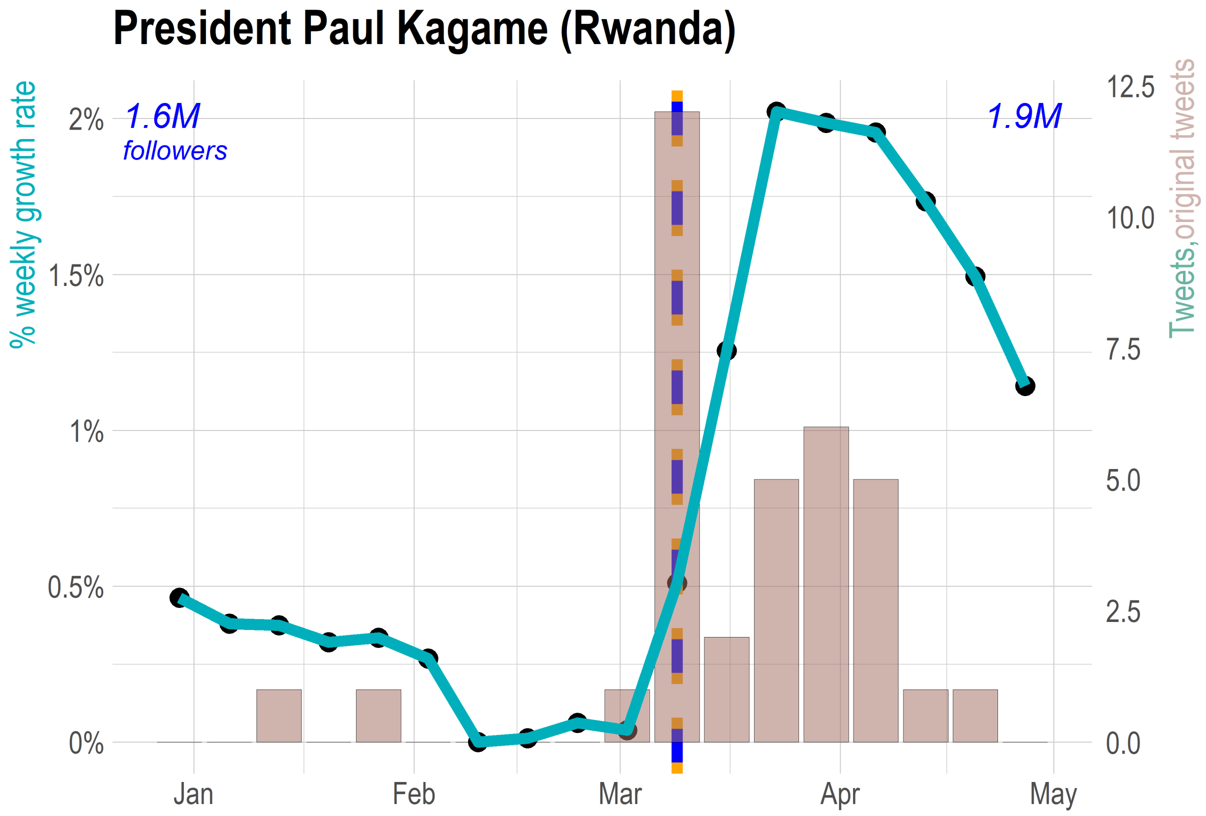

President Paul Kagame (Rwanda)
% weekly growth rate
Tweets, original tweets
1.6M
followers
1.9M
2%
1.5%
1%
0.5%
0%
12.5
10.0
7.5
5.0
2.5
0.0
Jan
Feb
Mar
Apr
May

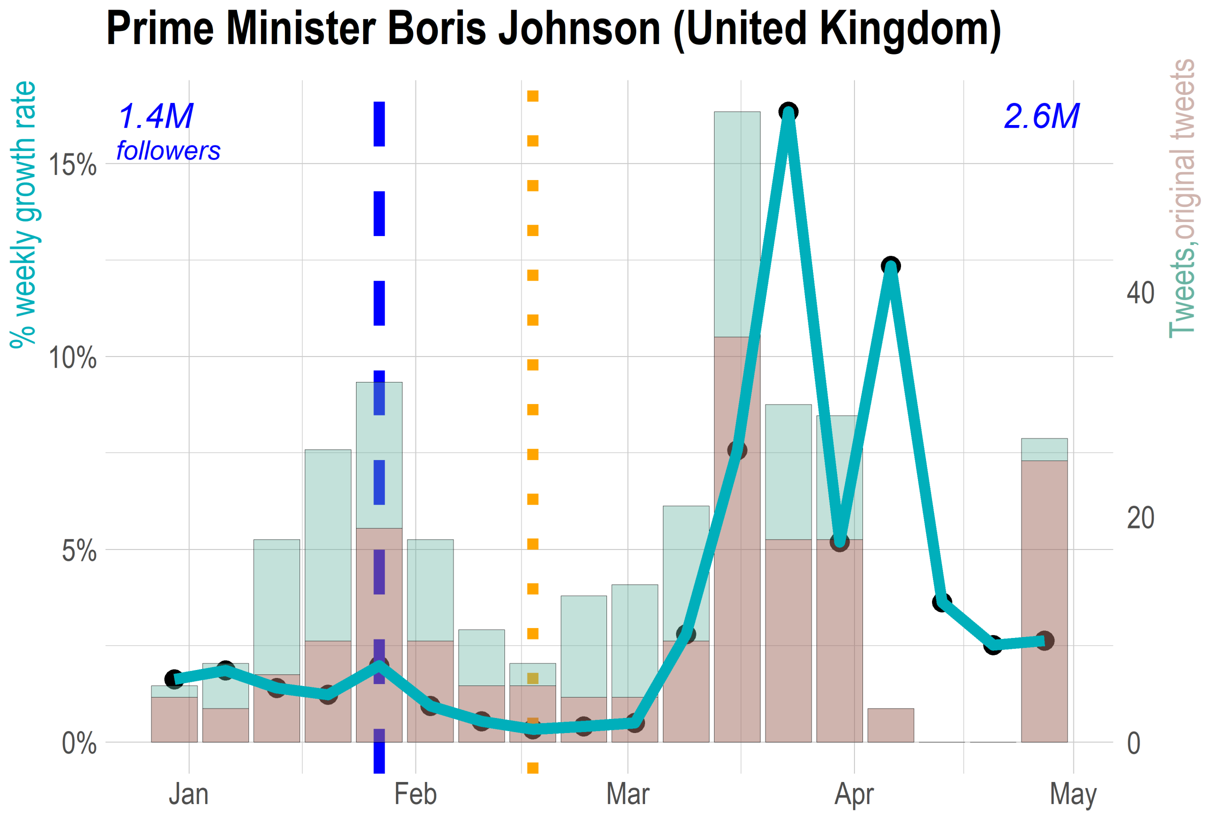
Prime Minister Boris Johnson (United Kingdom)
% weekly growth rate
Tweets, original tweets
1.4M
followers
2.6M
15%
10%
5%
0%
40
20
0
Jan
Feb
Mar
Apr
May

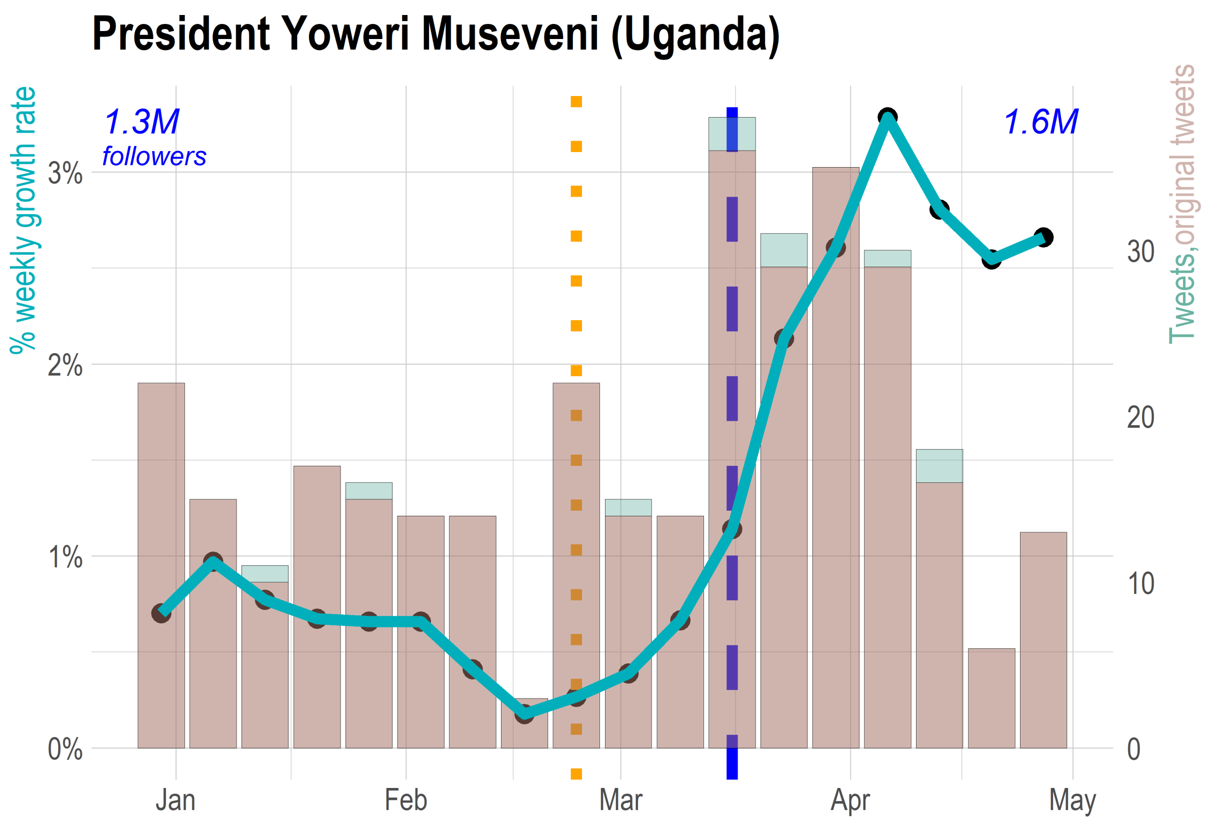
President Yoweri Museveni (Uganda)
% weekly growth rate
Tweets, original tweets
1.3M
followers
1.6M
3%
2%
1%
0%
30
20
10
0
Jan
Feb
Mar
Apr
May

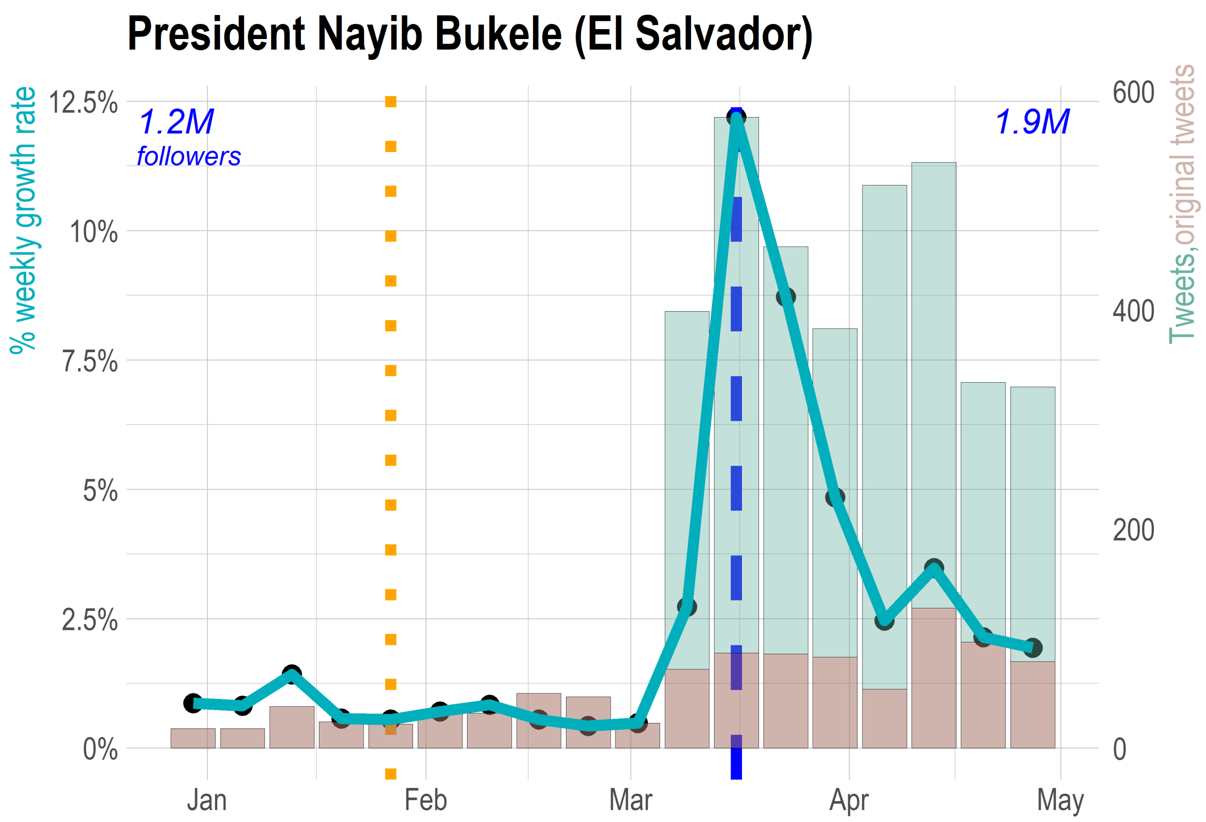

President Nayib Bukele (El Salvador)
% weekly growth rate
Tweets, original tweets
1.2M
followers
1.9M
12.5%
10%
7.5%
5%
2.5%
0%
600
400
200
0
Jan
Feb
Mar
Apr
May

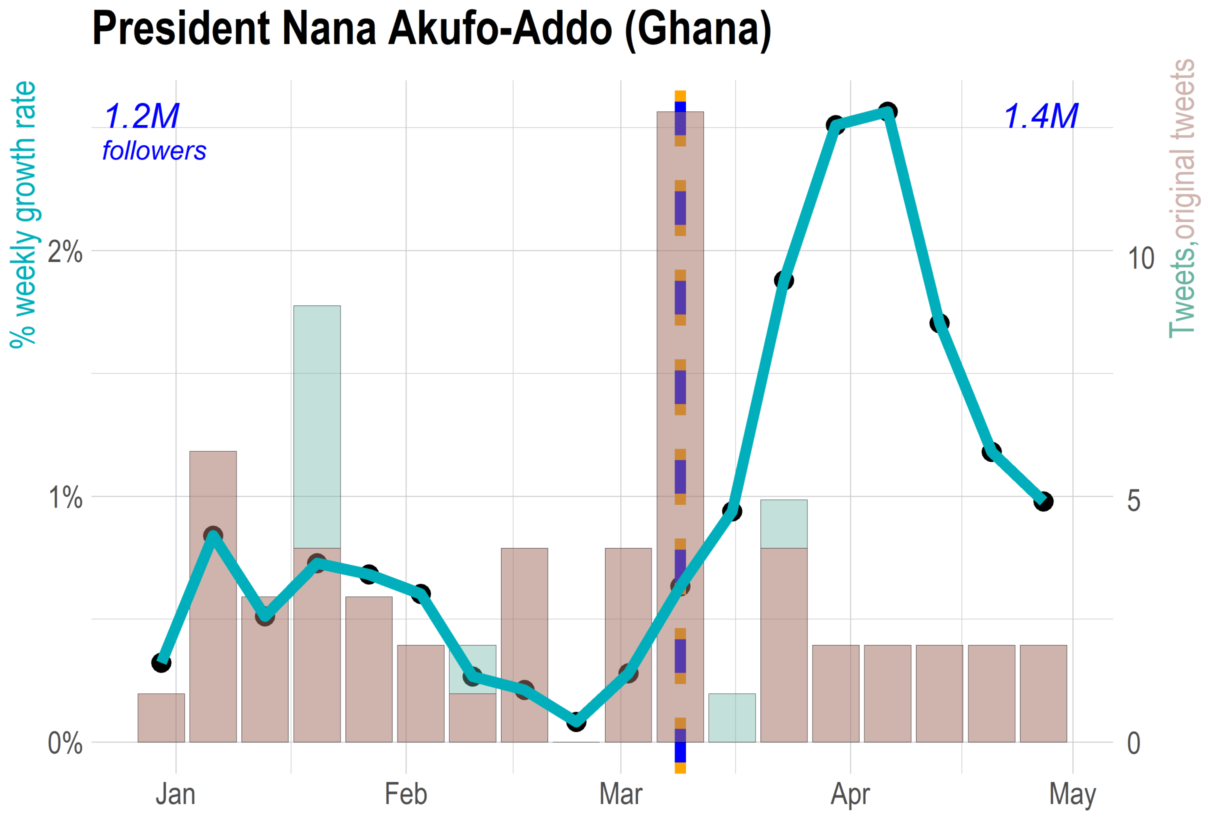

President Nana Akufo-Addo (Ghana)
1.2M
followers
1.4M
% weekly growth rate
Tweets, original tweets
2%
1%
0%
10
5
0
Jan
Feb
Mar
Apr
May

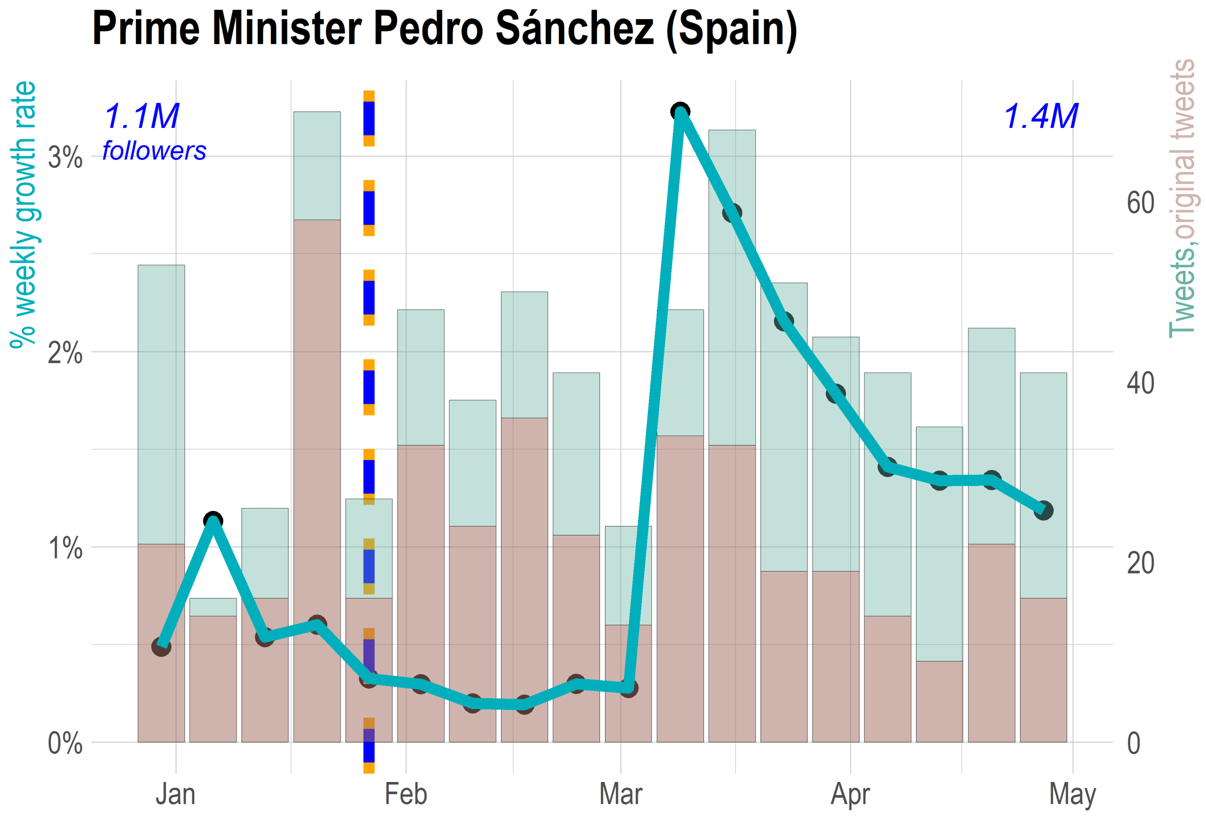

Prime Minister Pedro Sánchez (Spain)
% weekly growth rate
Tweets, original tweets
1.1M
followers
1.4M
3%
2%
1%
0%
60
40
20
0
Jan
Feb
Mar
Apr
May

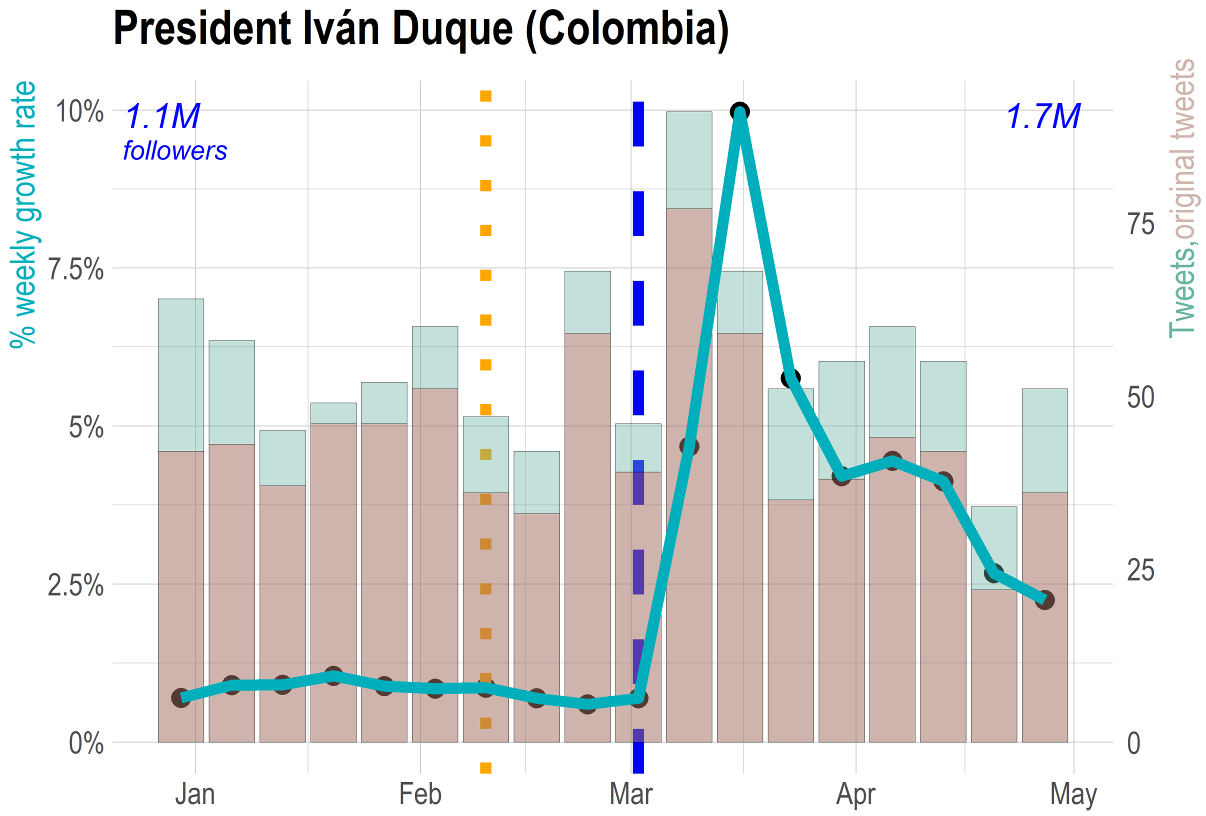
President Iván Duque (Colombia)
% weekly growth rate
Tweets, original tweets
1.1M
followers
1.7M
10%
7.5%
5%
2.5%
0%
75
50
25
0
Jan
Feb
Mar
Apr
May

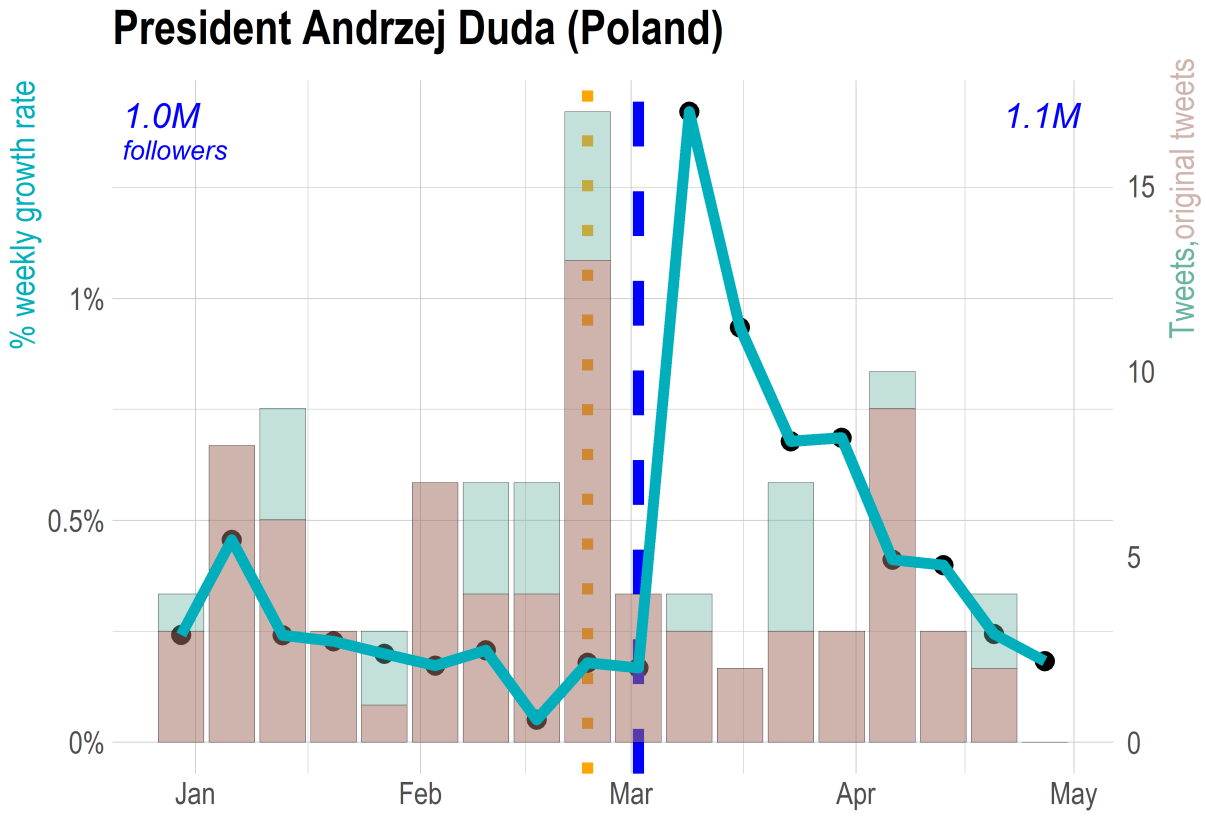

President Andrzej Duda (Poland)
% weekly growth rate
Tweets, original tweets
1.0M
followers
1.1M
1%
0.5%
0%
15
10
5
0
Jan
Feb
Mar
Apr
May

President Macky Sall (Senegal)

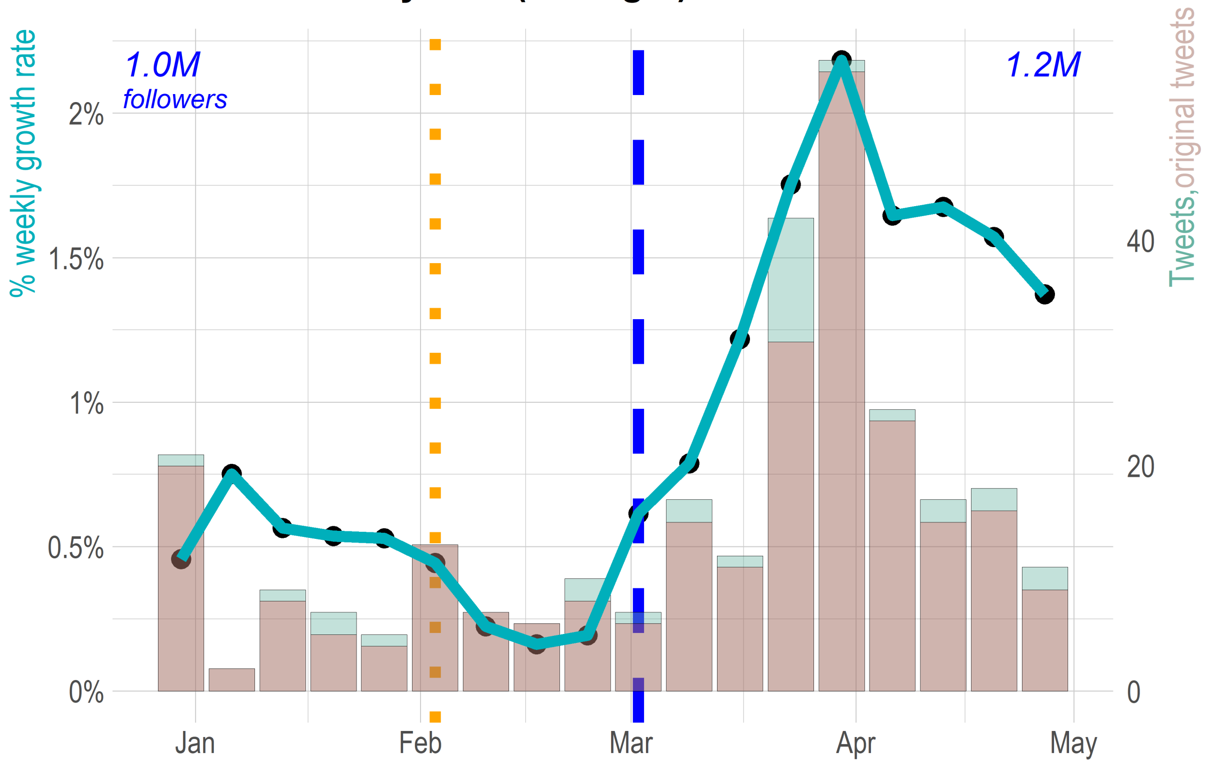

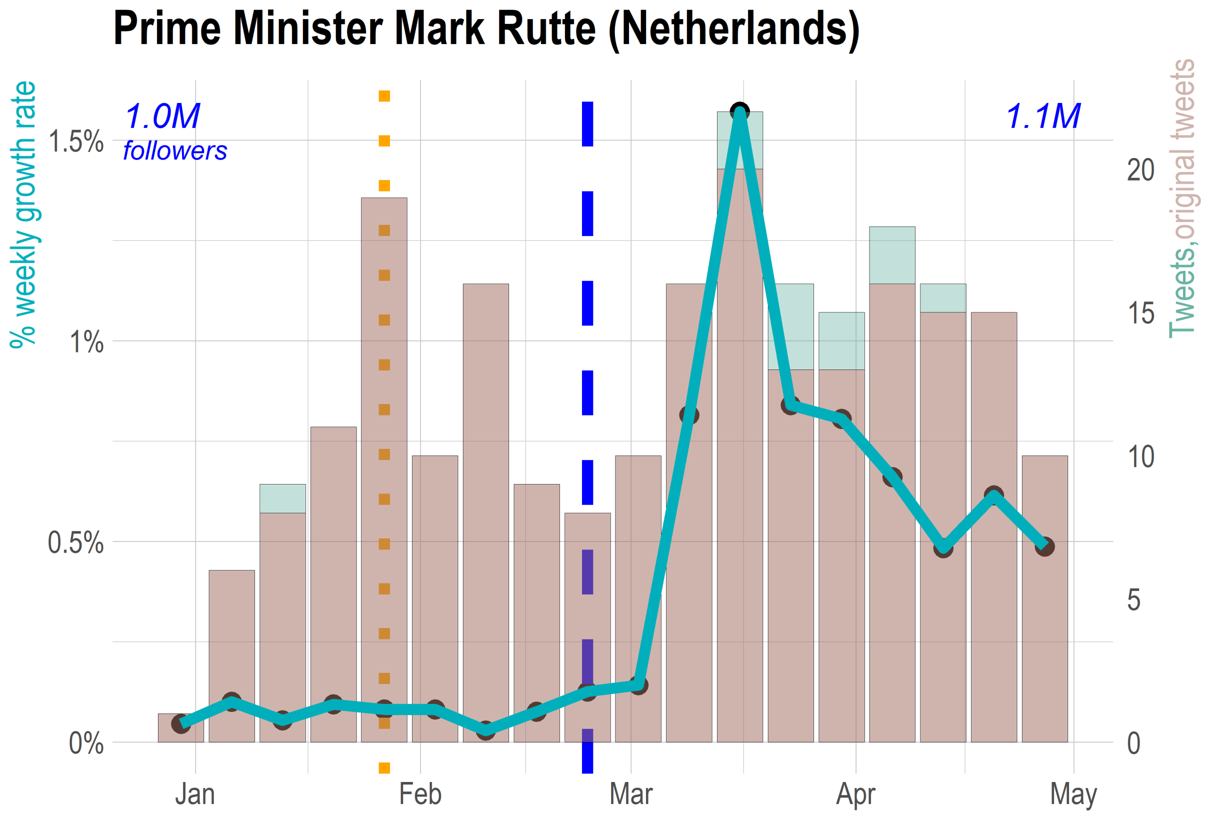
Prime Minister Mark Rutte (Netherlands)
1.0M
followers
1.1M
% weekly growth rate
Tweets, original tweets
1.5%
1%
0.5%
0%
20
15
10
5
0
Jan
Feb
Mar
Apr
May

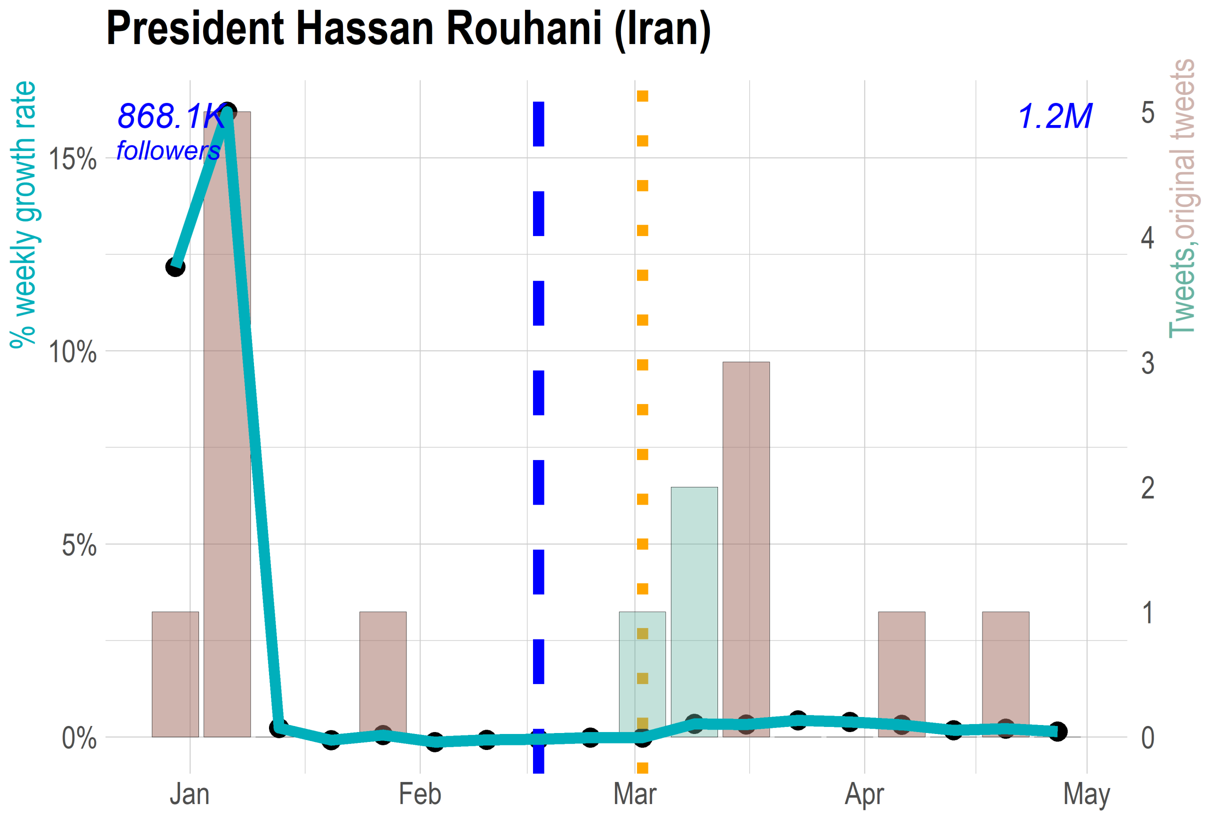

President Hassan Rouhani (Iran)
% weekly growth rate
Tweets, original tweets
868.1K
followers
1.2M
15%
10%
5%
0%
5
4
3
2
1
0
Jan
Feb
Mar
Apr
May

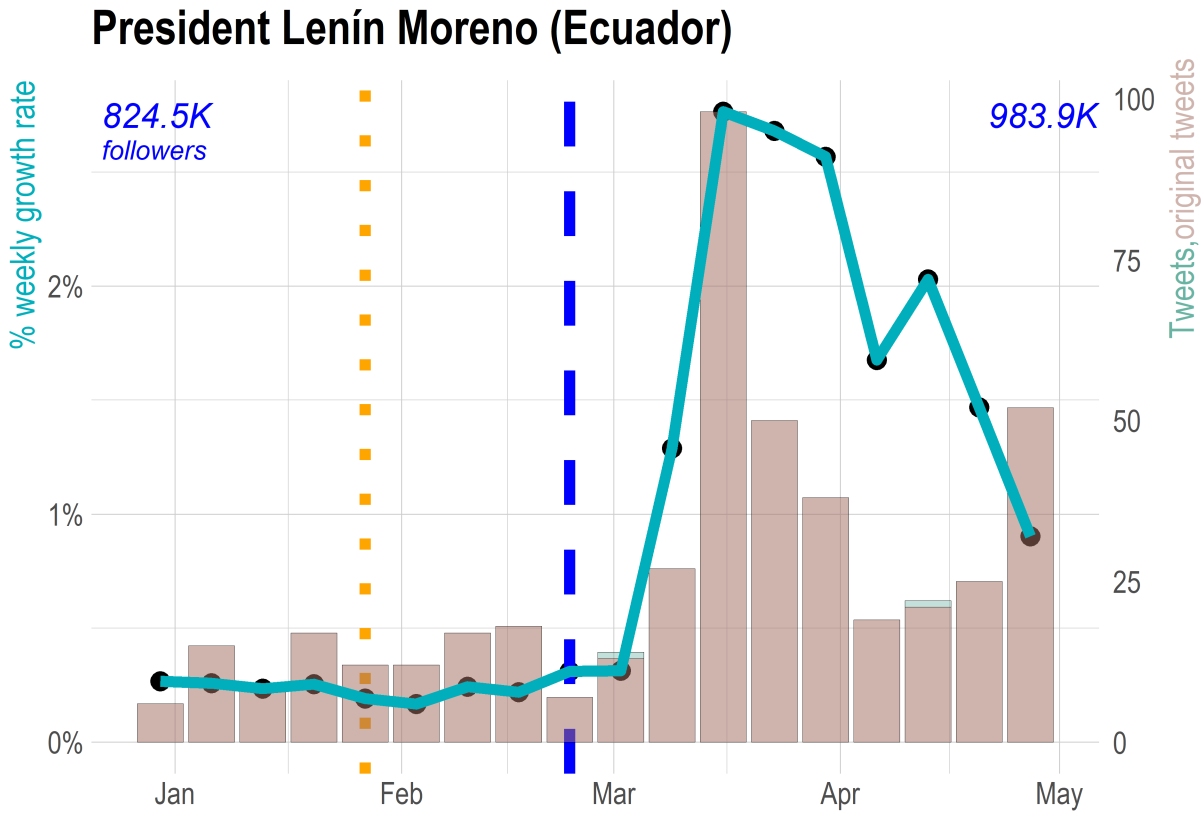
President Lenín Moreno (Ecuador)
824.5K
followers
983.9K
% weekly growth rate
Tweets, original tweets
0%
1%
2%
0
25
50
75
100
Jan
Feb
Mar
Apr
May

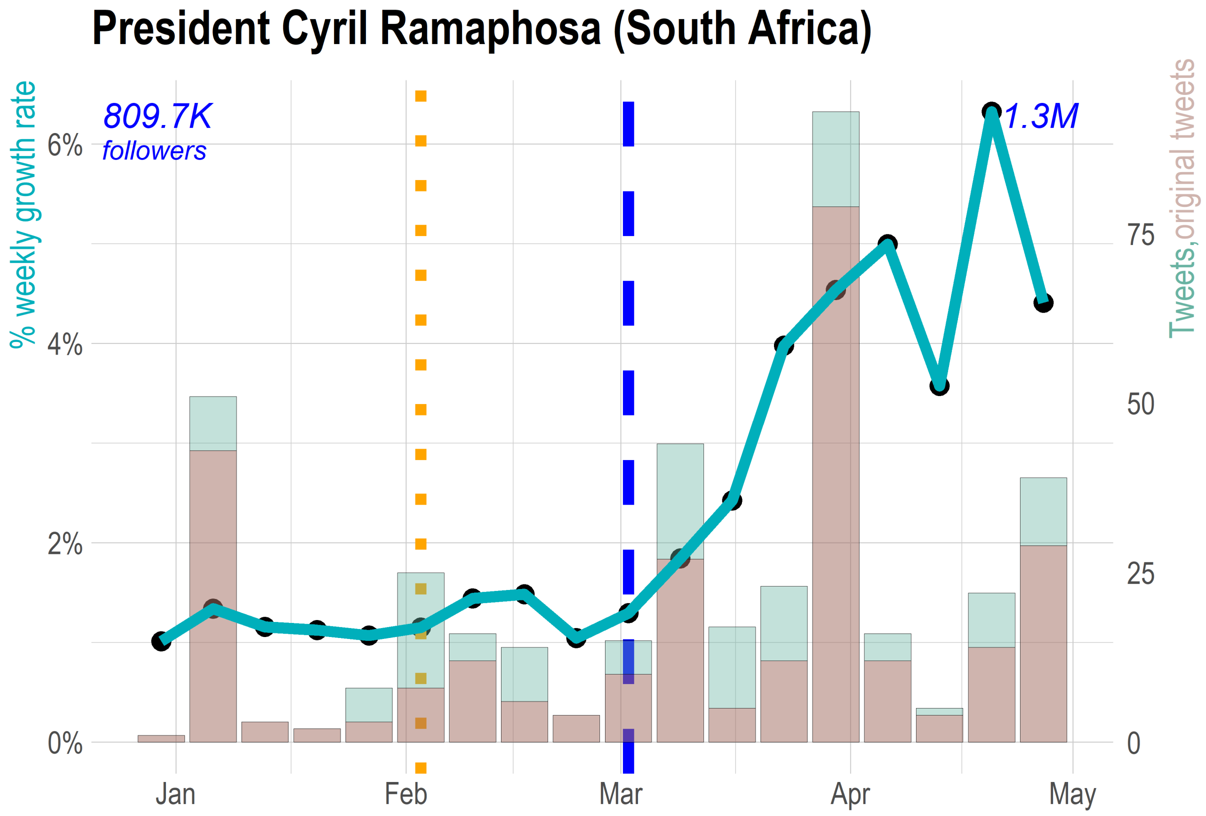

President Cyril Ramaphosa (South Africa)
809.7K
followers
1.3M
% weekly growth rate
Tweets, original tweets
6%
4%
2%
0%
75
50
25
0
Jan
Feb
Mar
Apr
May

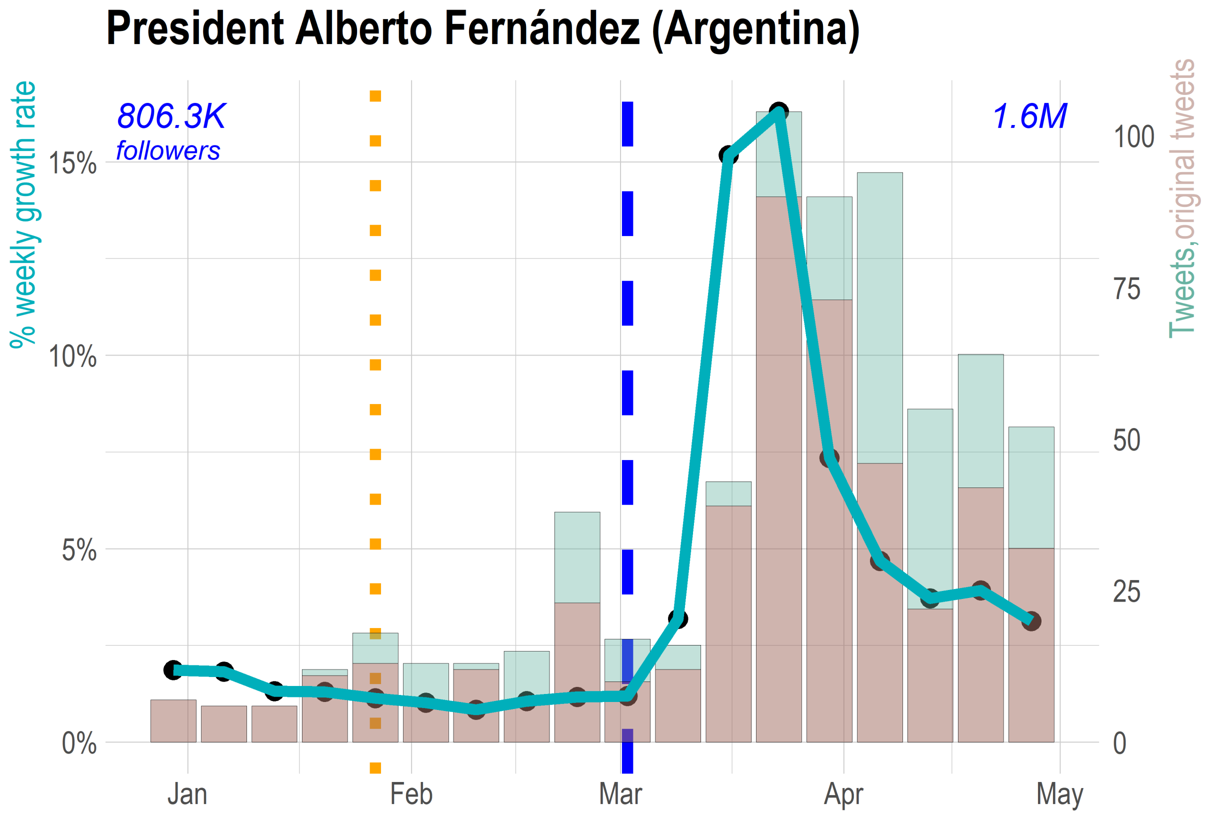
President Alberto Fernández (Argentina)
806.3K
followers
1.6M
% weekly growth rate
Tweets, original tweets
15%
10%
5%
0%
100
75
50
25
0
Jan
Feb
Mar
Apr
May

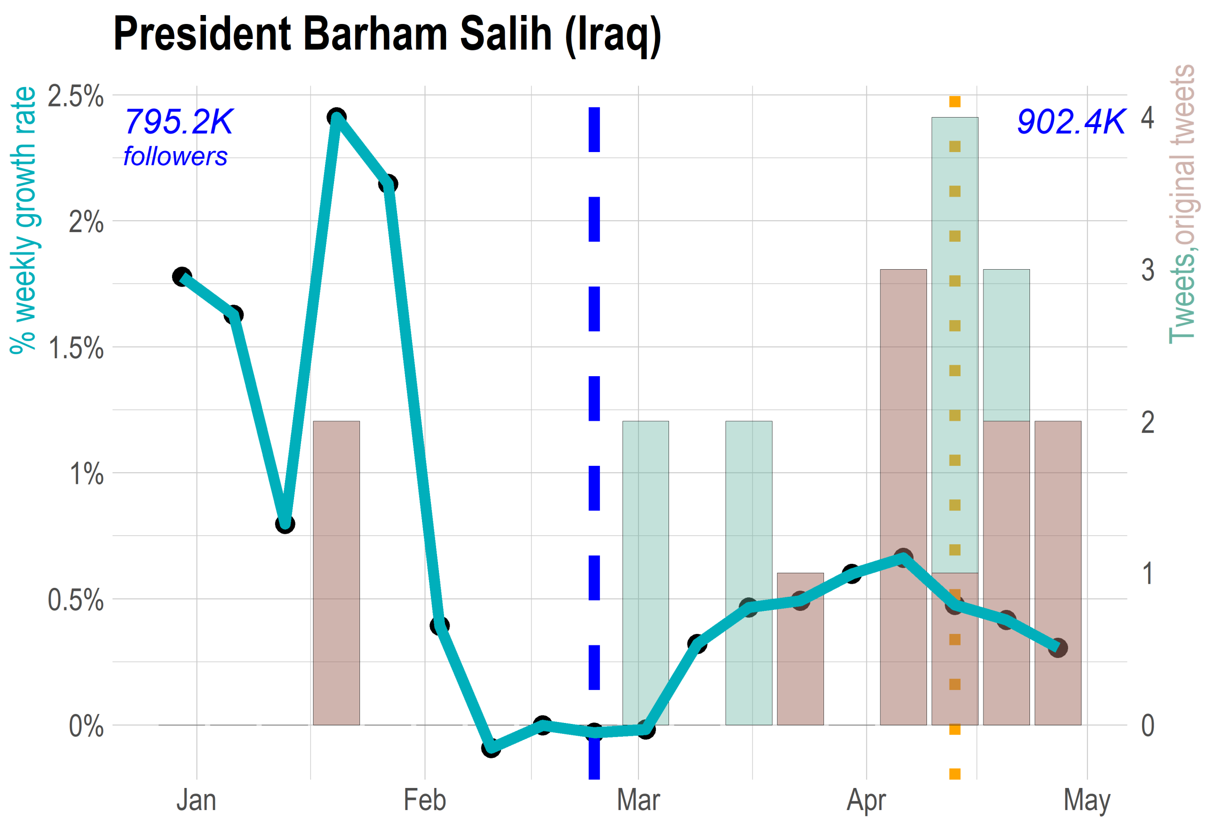

President Barham Salih (Iraq)
% weekly growth rate
2.5%
2%
1.5%
1%
0.5%
0%
795.2K
followers
902.4K
Tweets, original tweets
4
3
2
1
0
Jan
Feb
Mar
Apr
May

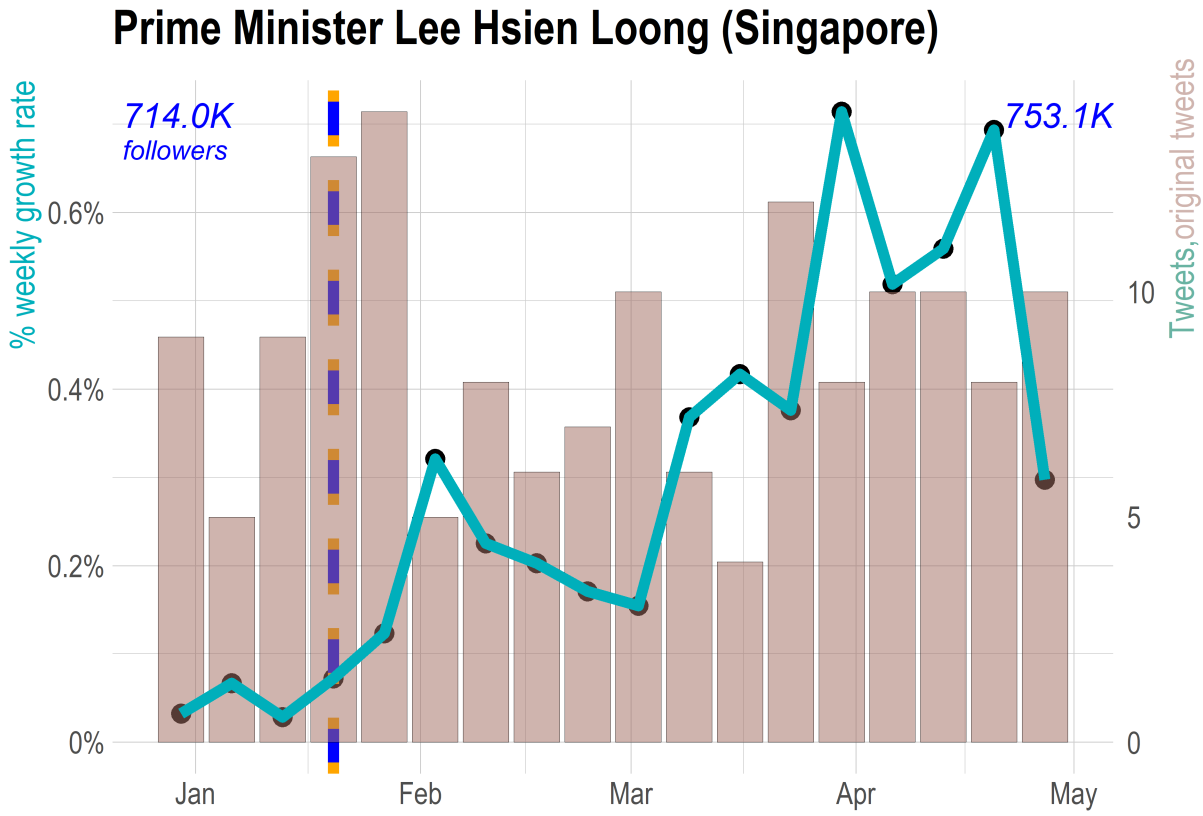

Prime Minister Lee Hsien Loong (Singapore)
% weekly growth rate
Tweets, original tweets
714.0K
followers
753.1K
0.6%
0.4%
0.2%
0%
10
5
0
Jan
Feb
Mar
Apr
May

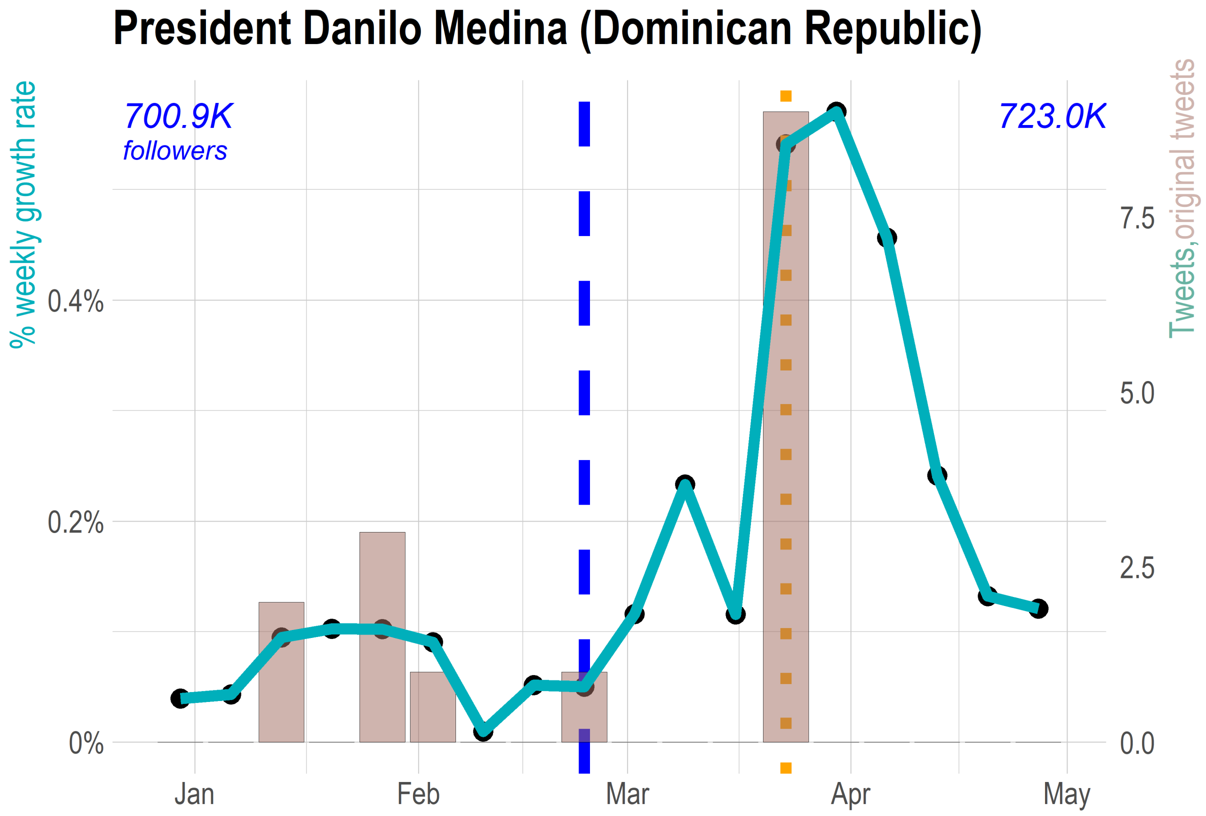

President Danilo Medina (Dominican Republic)
700.9K
followers
723.0K
% weekly growth rate
0.4%
0.2%
0%
Tweets, original tweets
7.5
5.0
2.5
0.0
Jan
Feb
Mar
Apr
May

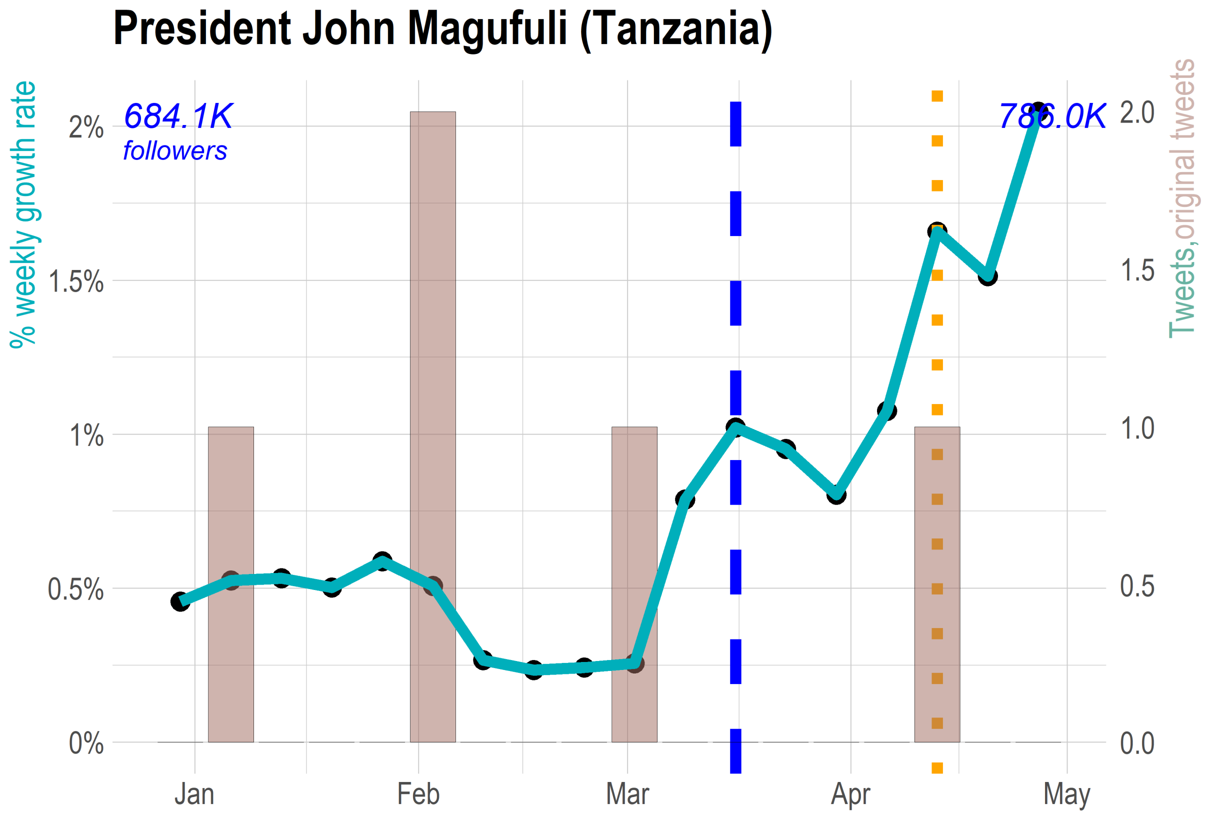

President John Magufuli (Tanzania)
% weekly growth rate
Tweets, original tweets
684.1K
followers
786.0K
2%
1.5%
1%
0.5%
0%
2.0
1.5
1.0
0.5
0.0
Jan
Feb
Mar
Apr
May

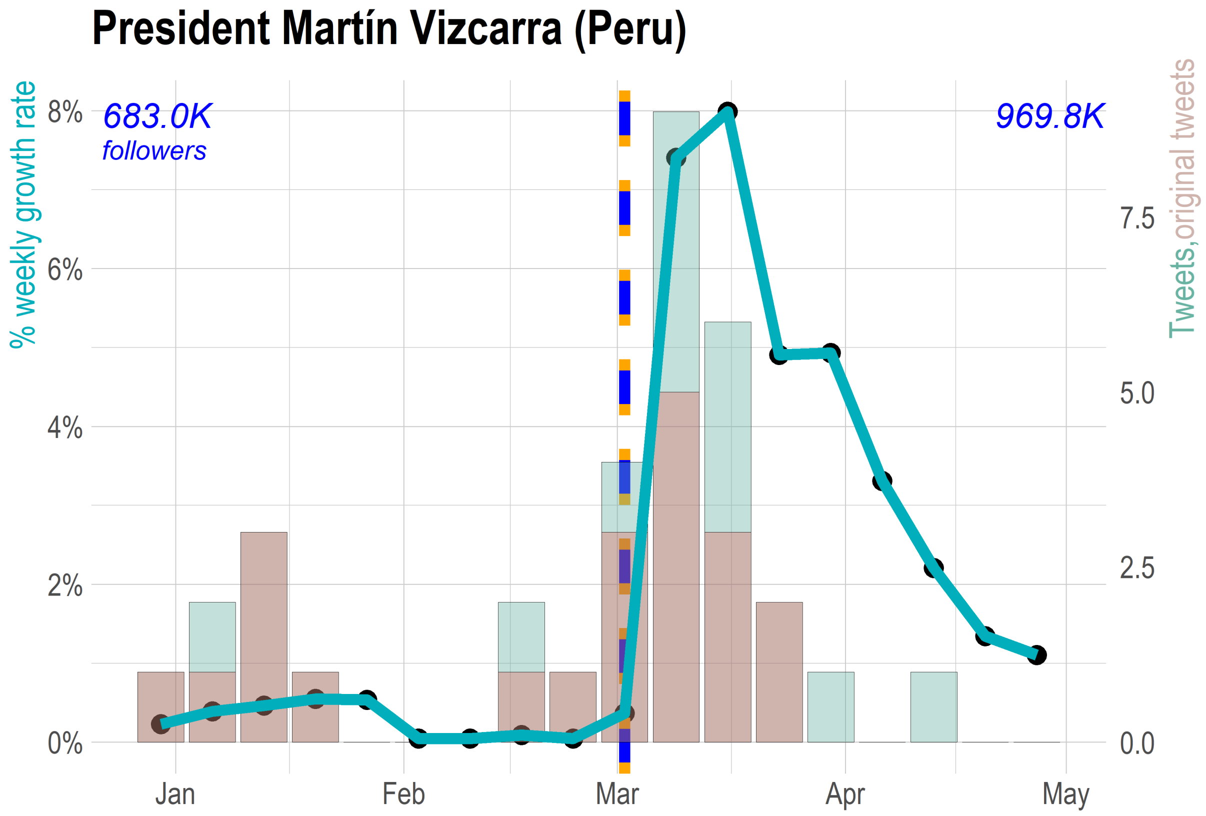
President Martín Vizcarra (Peru)
% weekly growth rate
Tweets, original tweets
683.0K
followers
969.8K
8%
6%
4%
2%
0%
7.5
5.0
2.5
0.0
Jan
Feb
Mar
Apr
May

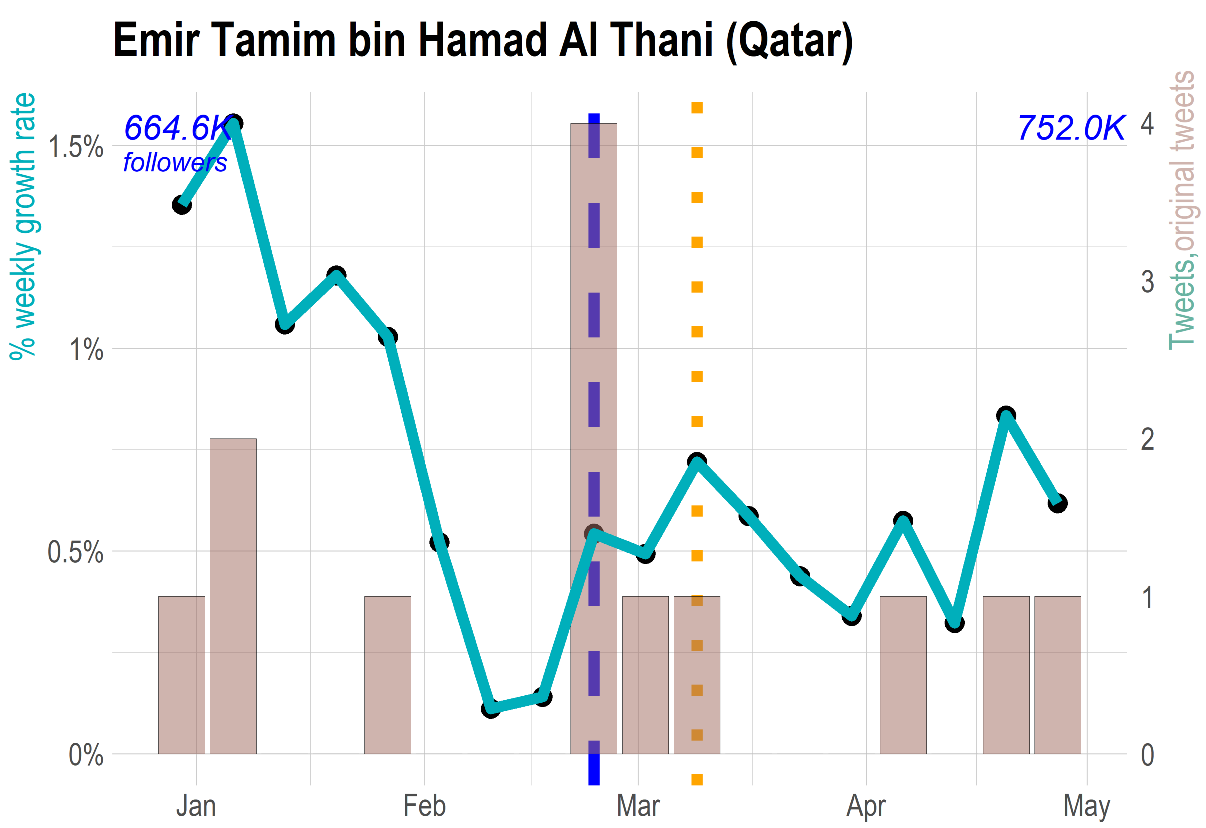

Emir Tamim bin Hamad Al Thani (Qatar)
% weekly growth rate
Tweets,original tweets
664.6K
followers
752.0K
1.5%
1%
0.5%
0%
4
3
2
1
0
Jan
Feb
Mar
Apr
May

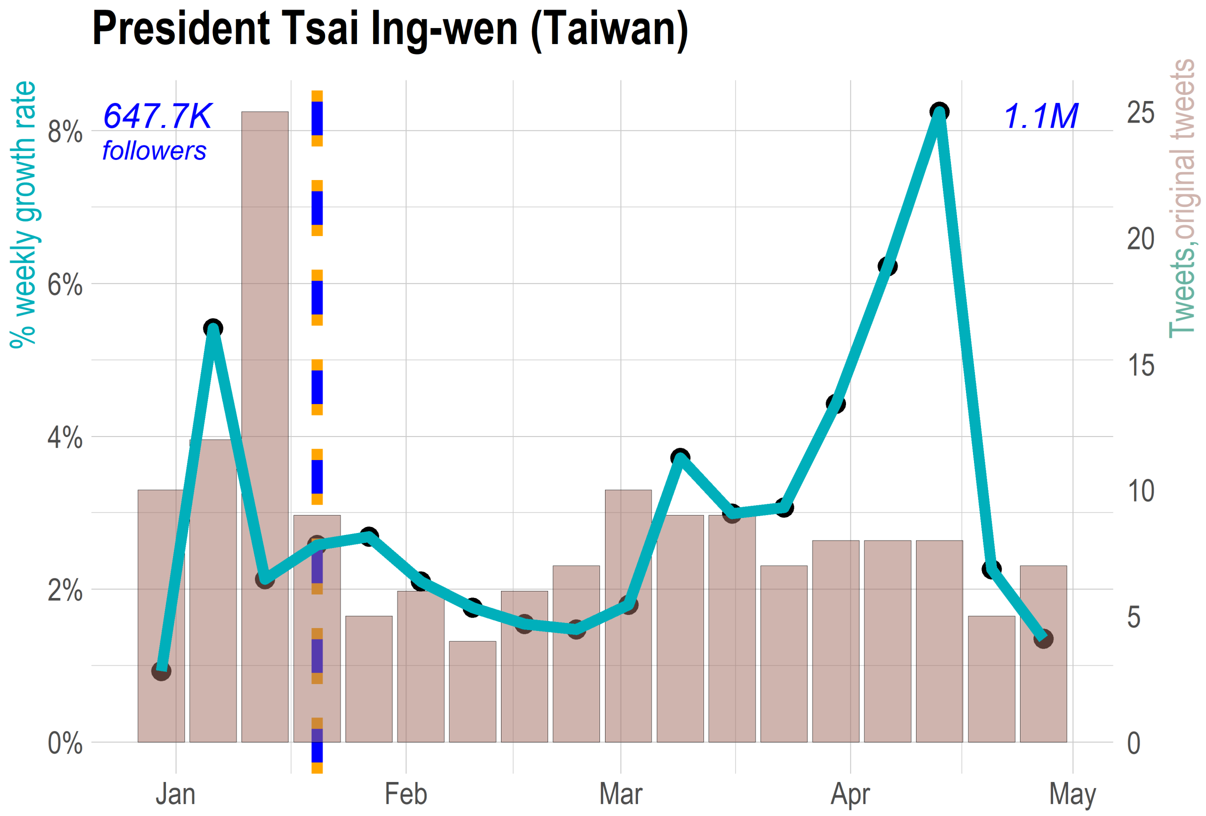
President Tsai Ing-wen (Taiwan)
% weekly growth rate
647.7K
followers
1.1M
8%
6%
4%
2%
0%
25
20
15
10
5
0
Tweets, original tweets
Jan
Feb
Mar
Apr
May

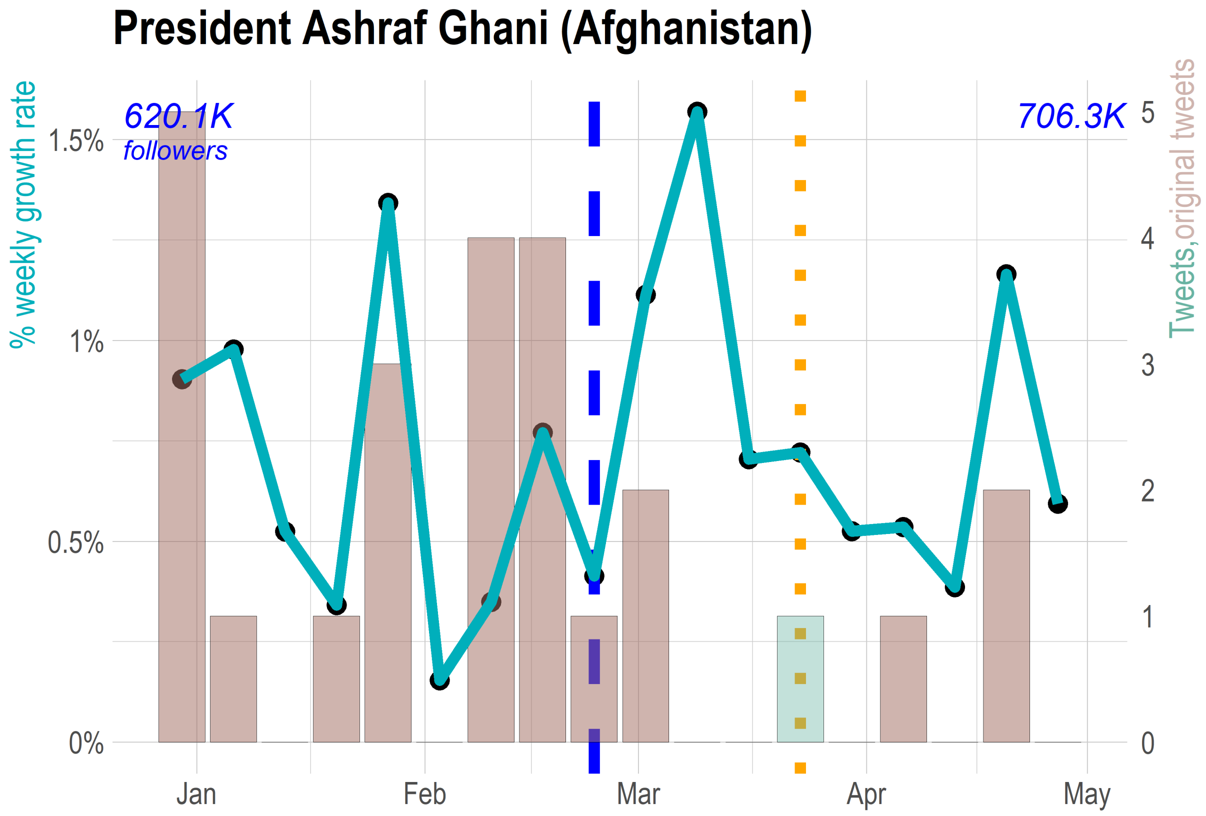

President Ashraf Ghani (Afghanistan)
% weekly growth rate
Tweets, original tweets
620.1K
followers
706.3K
1.5%
1%
0.5%
0%
5
4
3
2
1
0
Jan
Feb
Mar
Apr
May

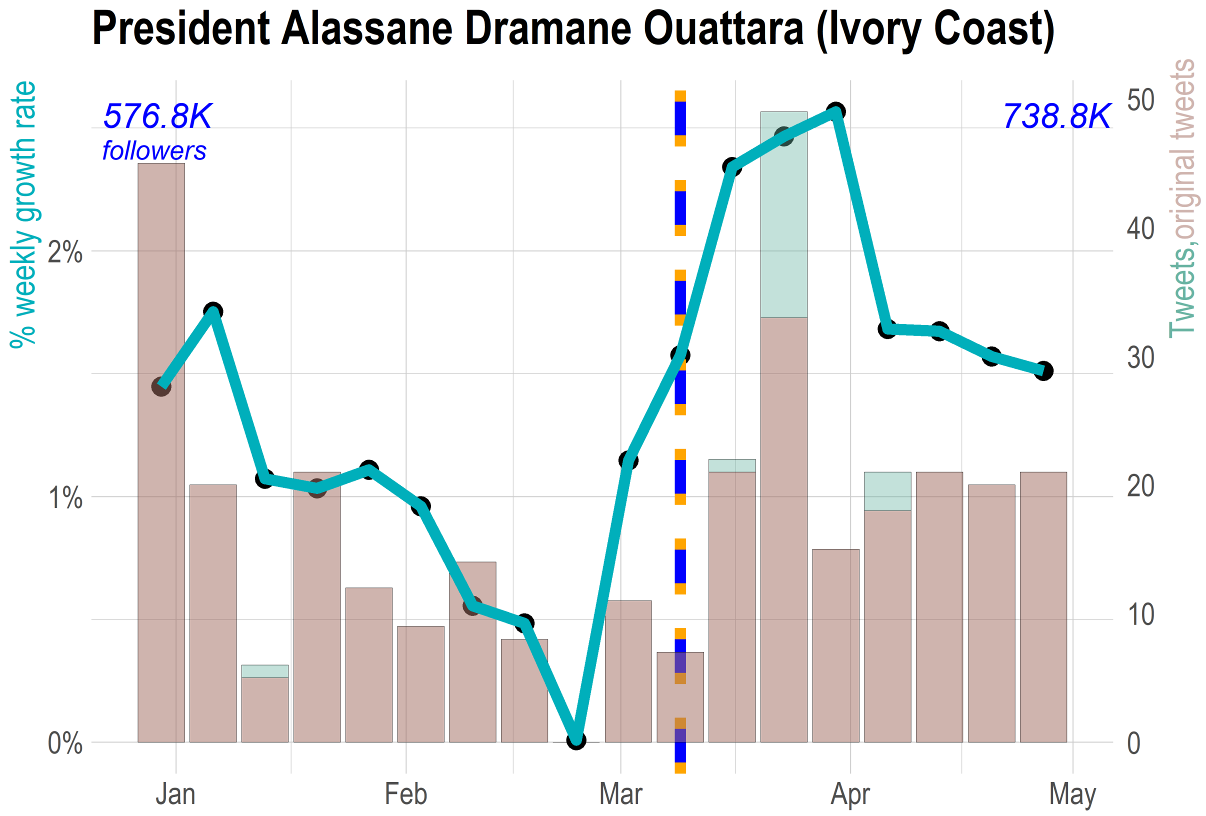

President Alassane Dramane Ouattara (Ivory Coast)
% weekly growth rate
Tweets, original tweets
576.8K
followers
738.8K
2%
1%
0%
50
40
30
20
10
0
Jan
Feb
Mar
Apr
May

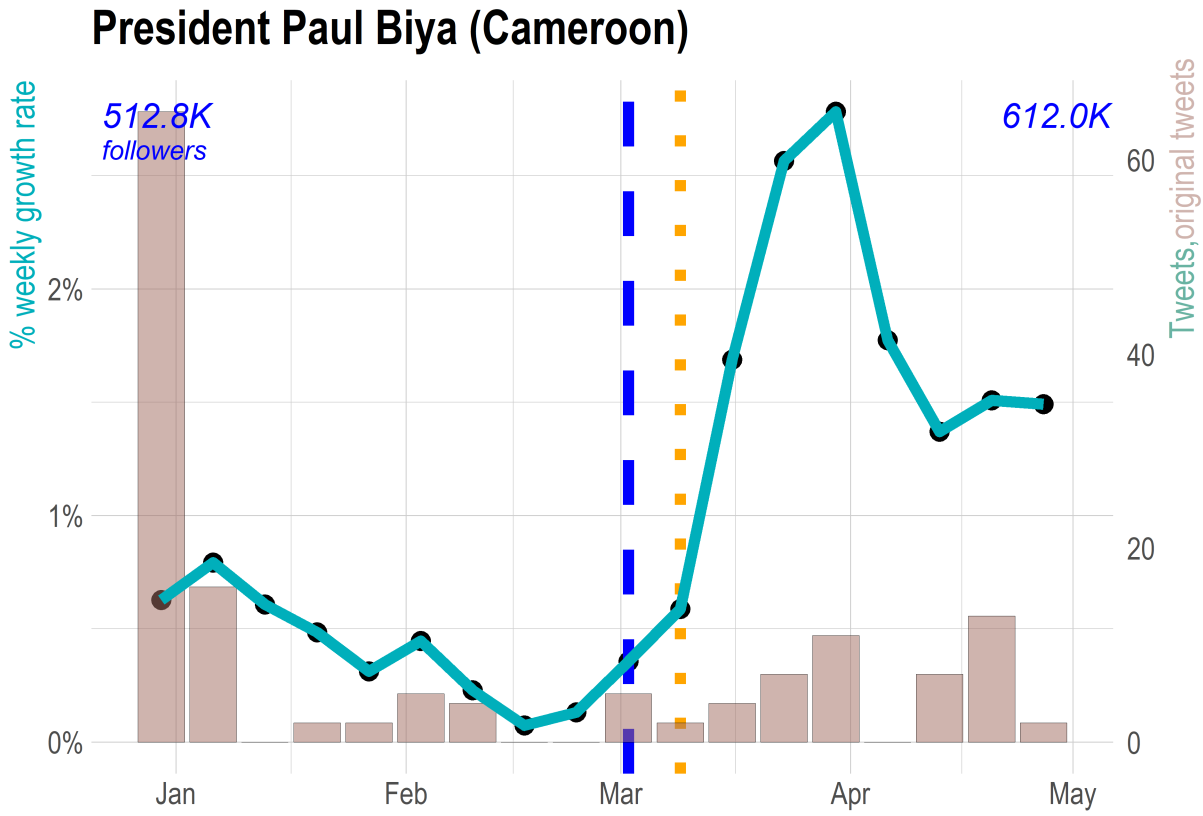

President Paul Biya (Cameroon)
% weekly growth rate
Tweets, original tweets
512.8K
followers
612.0K
2%
1%
0%
60
40
20
0
Jan
Feb
Mar
Apr
May

Prime Minister Kyriakos Mitsotakis (Greece)

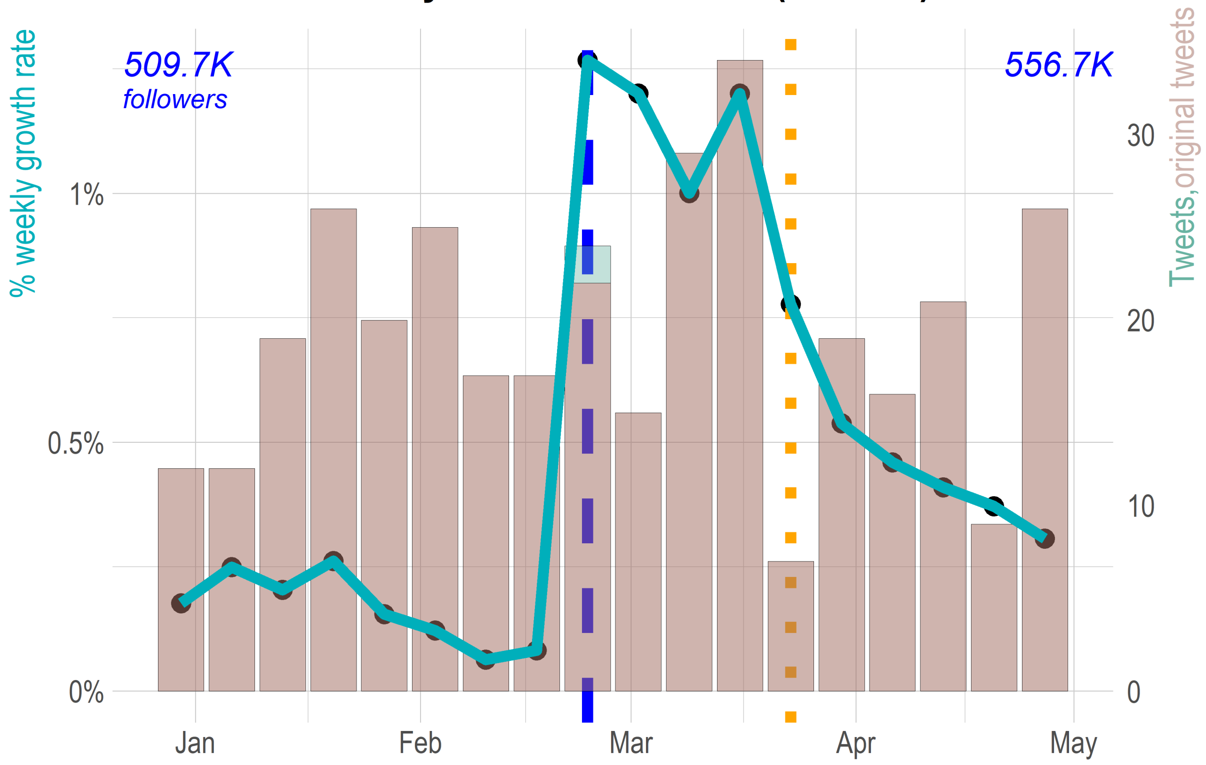

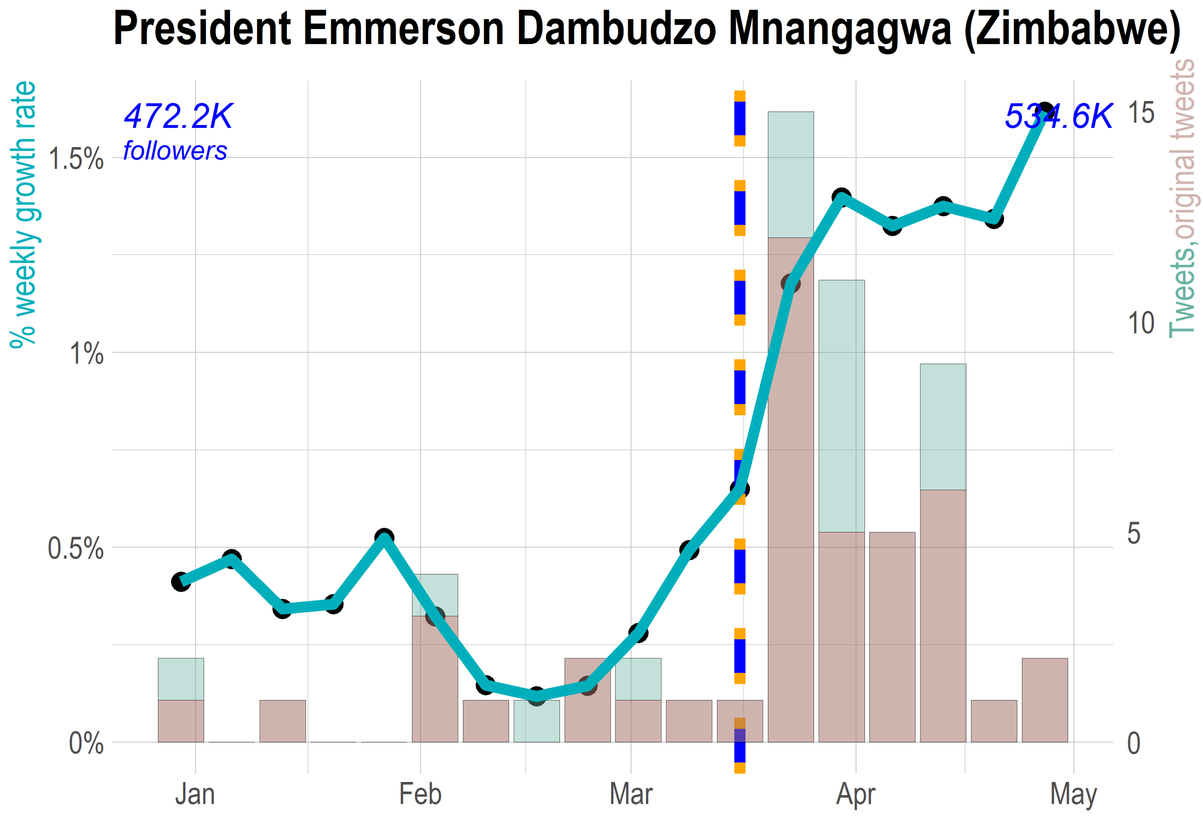

President Emmerson Dambudzo Mnangagwa (Zimbabwe)
% weekly growth rate
Tweets, original tweets
472.2K
followers
534.6K
1.5%
1%
0.5%
0%
15
10
5
0
Jan
Feb
Mar
Apr
May

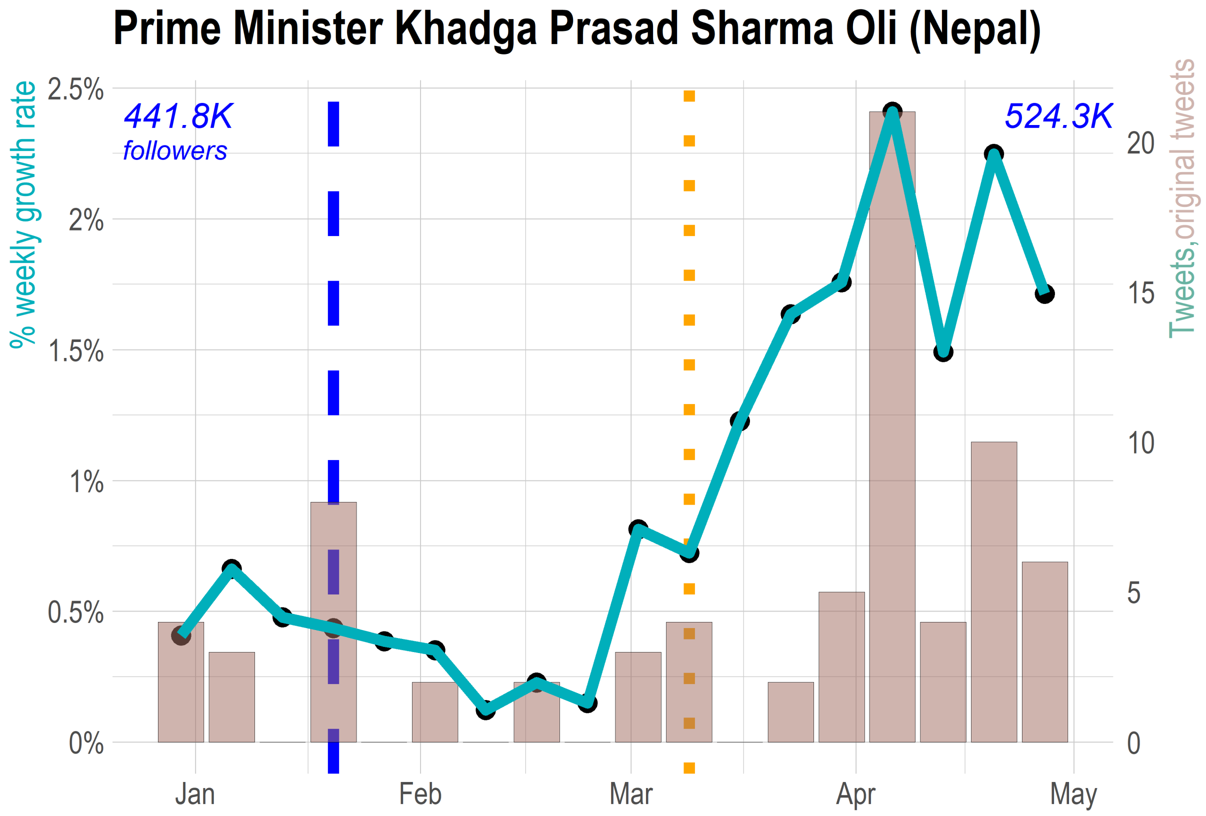

Prime Minister Khadga Prasad Sharma Oli (Nepal)
% weekly growth rate
Tweets, original tweets
441.8K
followers
524.3K
2.5%
2%
1.5%
1%
0.5%
0%
20
15
10
5
0
Jan
Feb
Mar
Apr
May

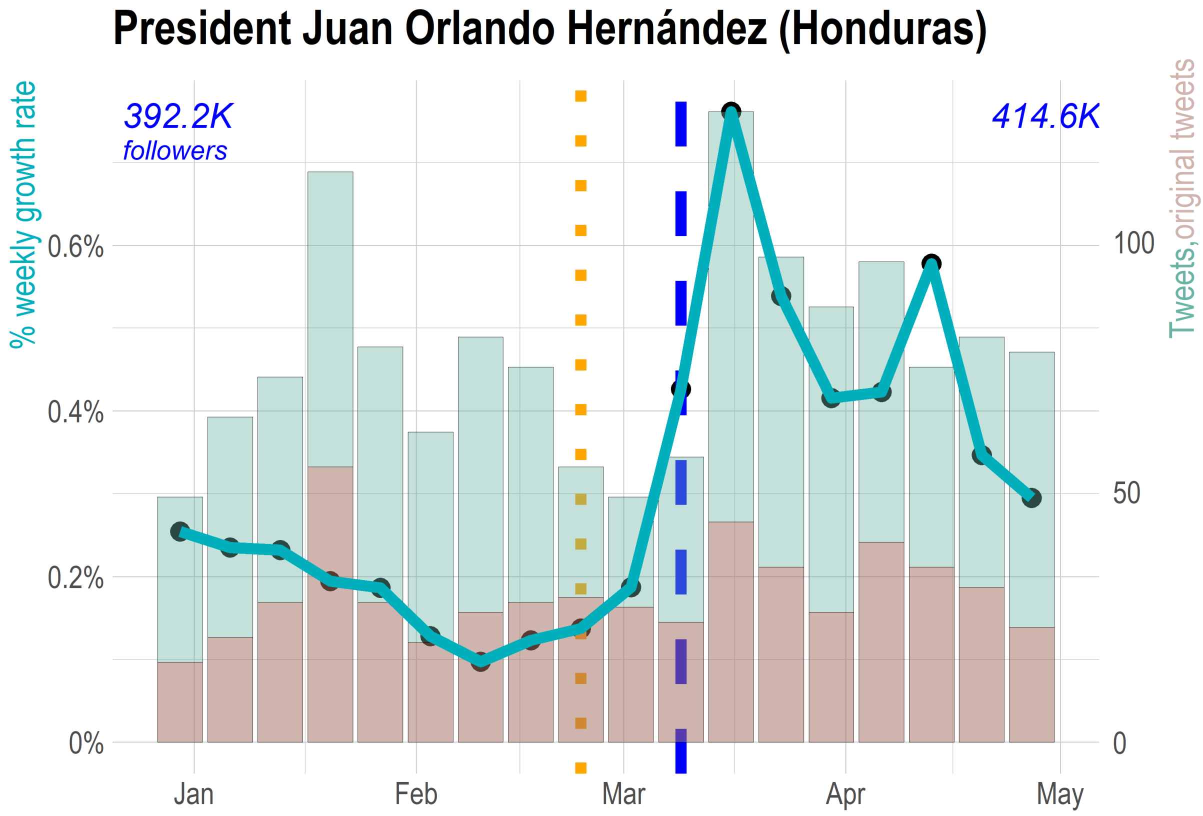

President Juan Orlando Hernández (Honduras)
392.2K
followers
414.6K
% weekly growth rate
Tweets, original tweets
0.6%
0.4%
0.2%
0%
100
50
0
Jan
Feb
Mar
Apr
May

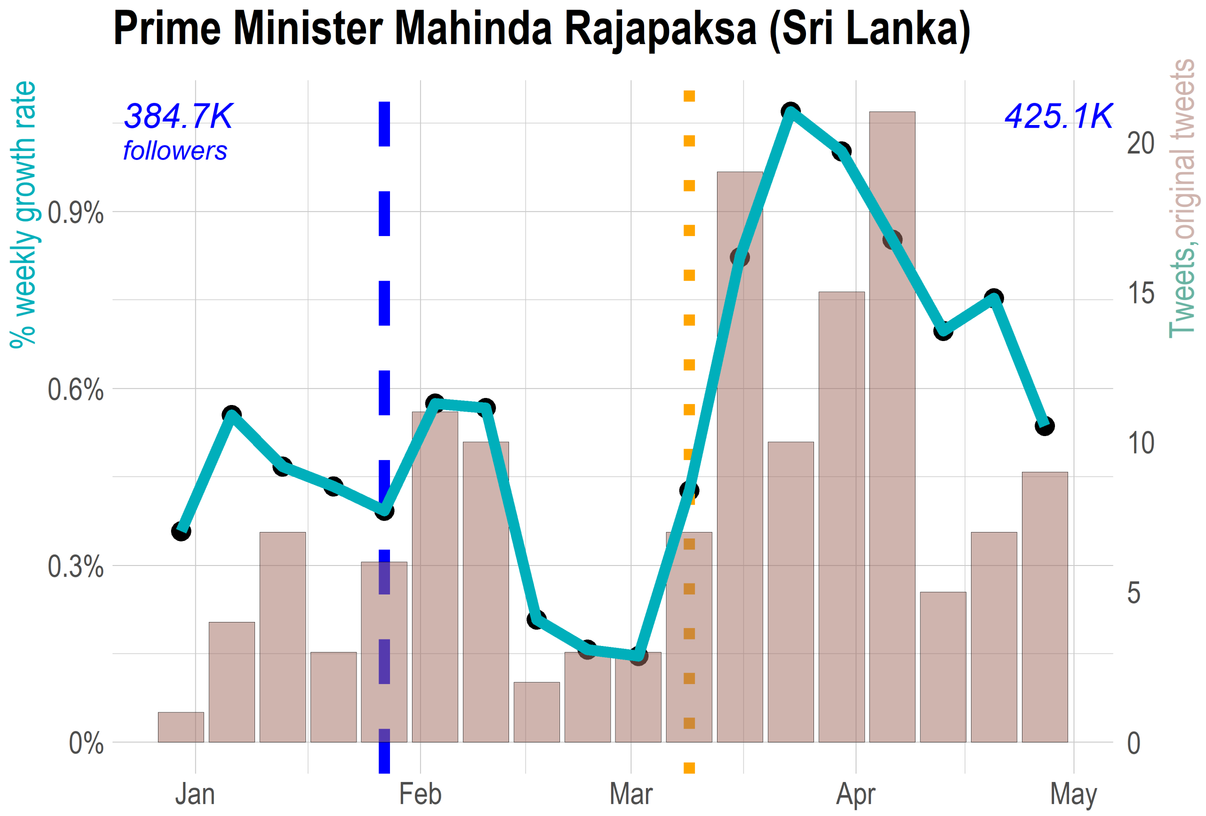
Prime Minister Mahinda Rajapaksa (Sri Lanka)
384.7K
followers
425.1K
% weekly growth rate
Tweets, original tweets
0%
0.3%
0.6%
0.9%
0
5
10
15
20
Jan
Feb
Mar
Apr
May

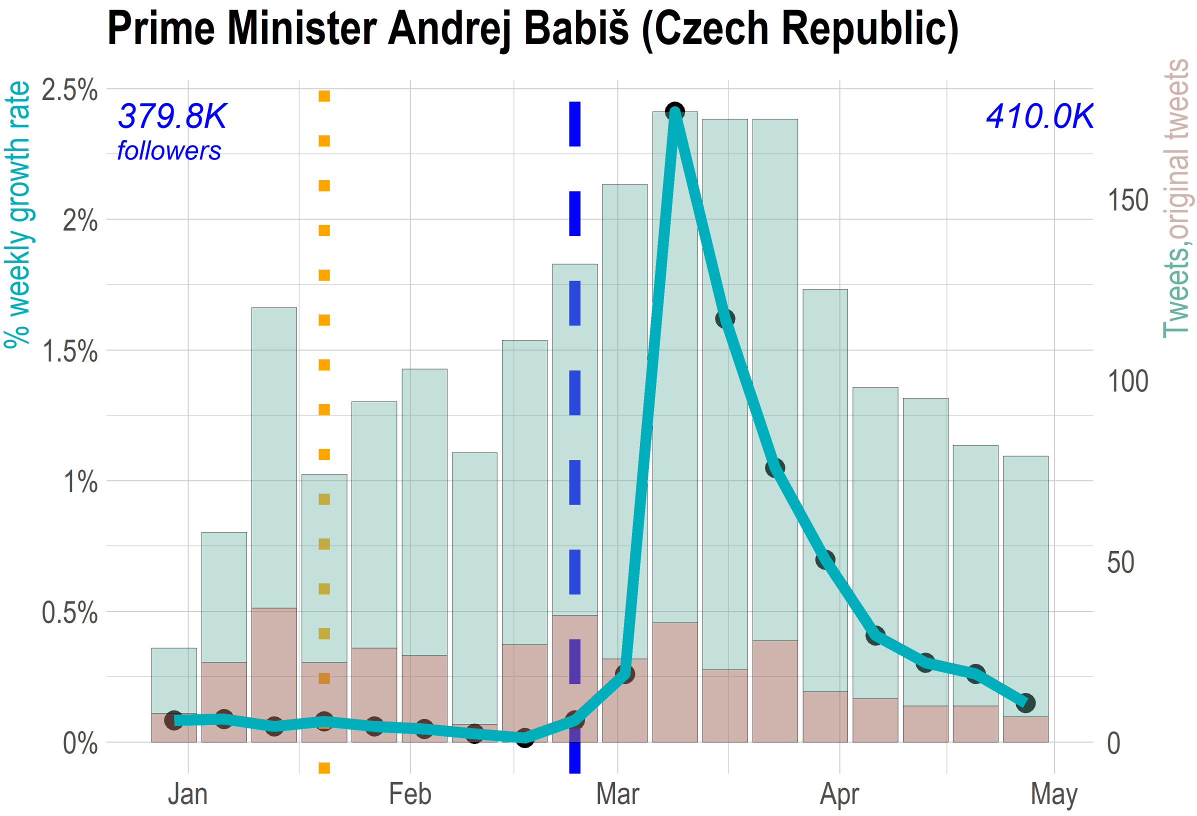

Prime Minister Andrej Babiš (Czech Republic)
% weekly growth rate
Tweets, original tweets
379.8K
followers
410.0K
2.5%
2%
1.5%
1%
0.5%
0%
150
100
50
0
Jan
Feb
Mar
Apr
May

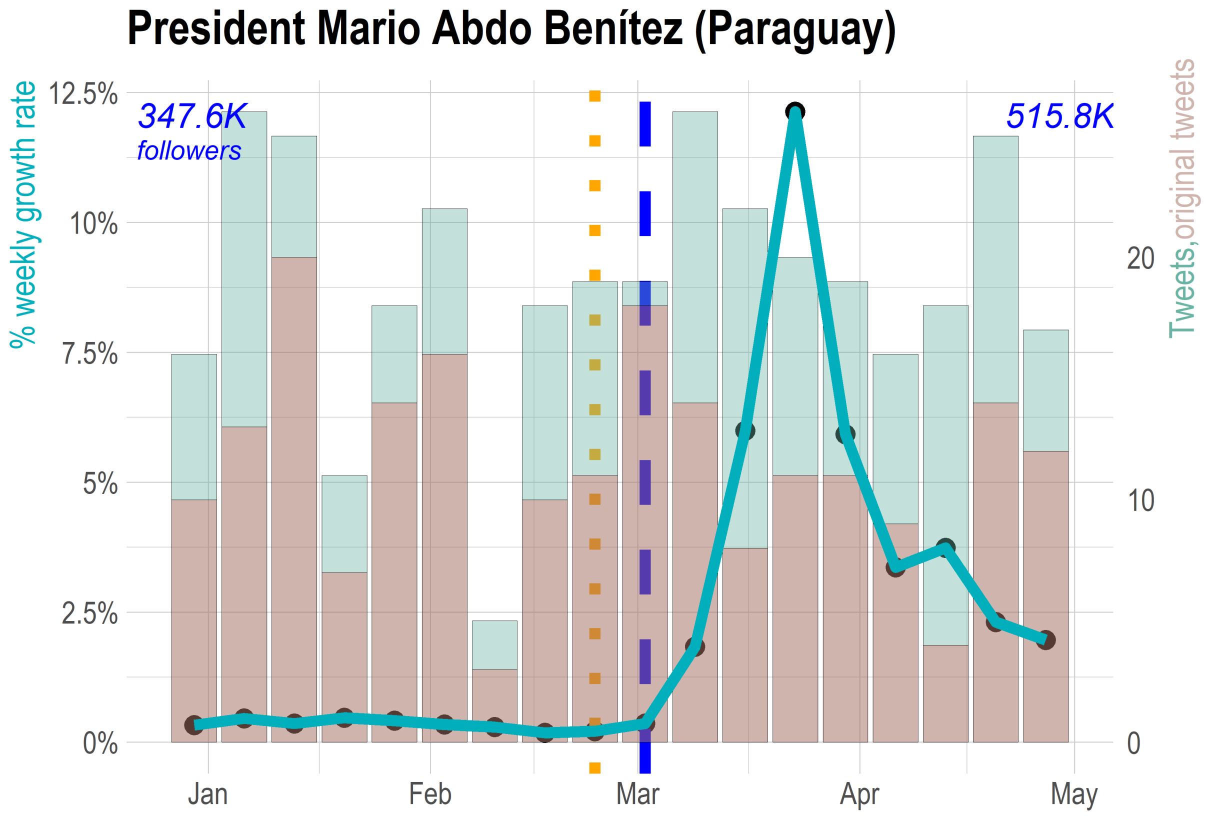

President Mario Abdo Benítez (Paraguay)
% weekly growth rate
Tweets, original tweets
347.6K
followers
515.8K
12.5%
10%
7.5%
5%
2.5%
0%
20
10
0
Jan
Feb
Mar
Apr
May

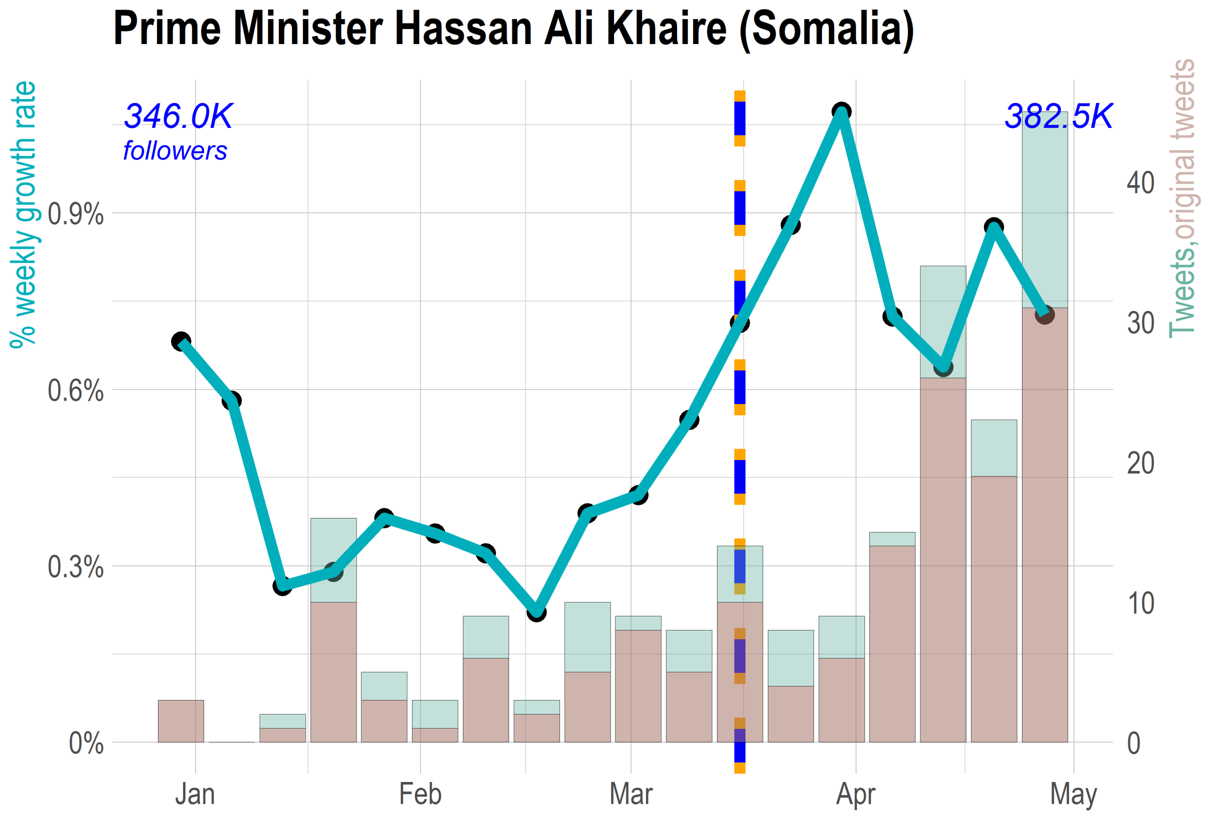

Prime Minister Hassan Ali Khaire (Somalia)
346.0K
followers
382.5K
% weekly growth rate
0.9%
0.6%
0.3%
0%
Tweets, original tweets
40
30
20
10
0
Jan
Feb
Mar
Apr
May

Prime Minister Edi Rama (Albania)

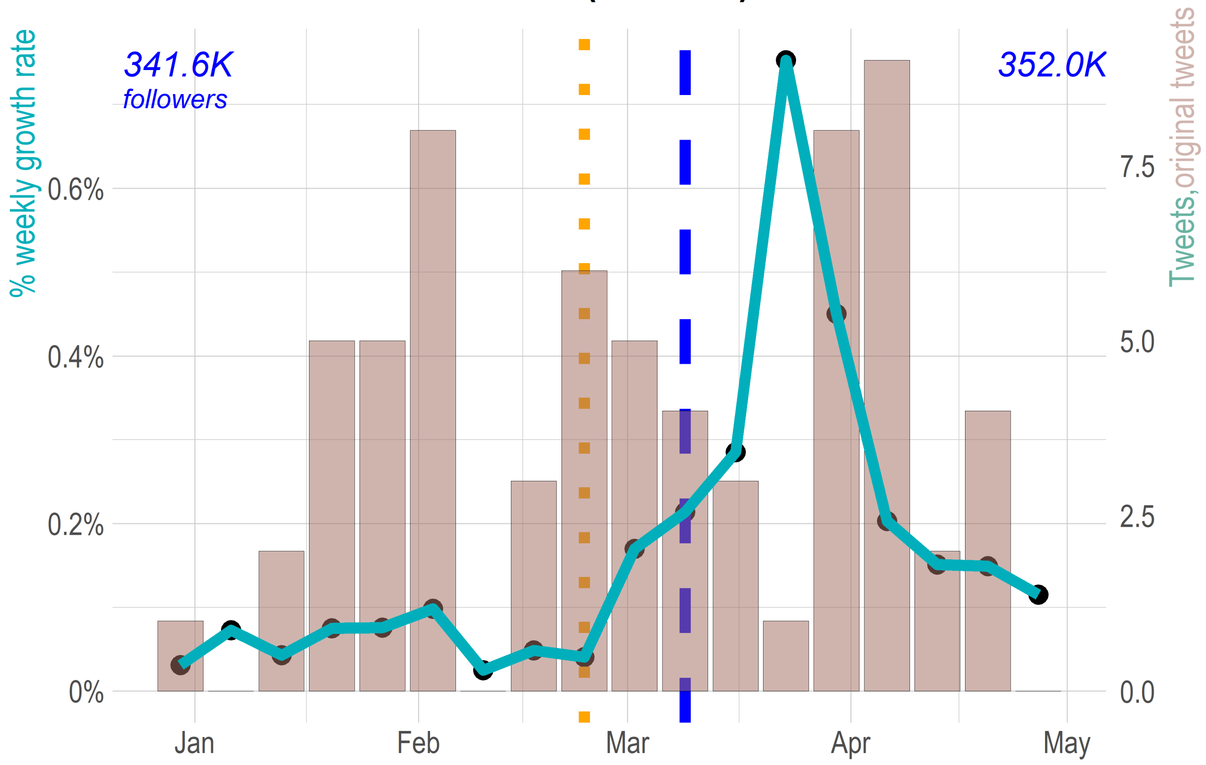

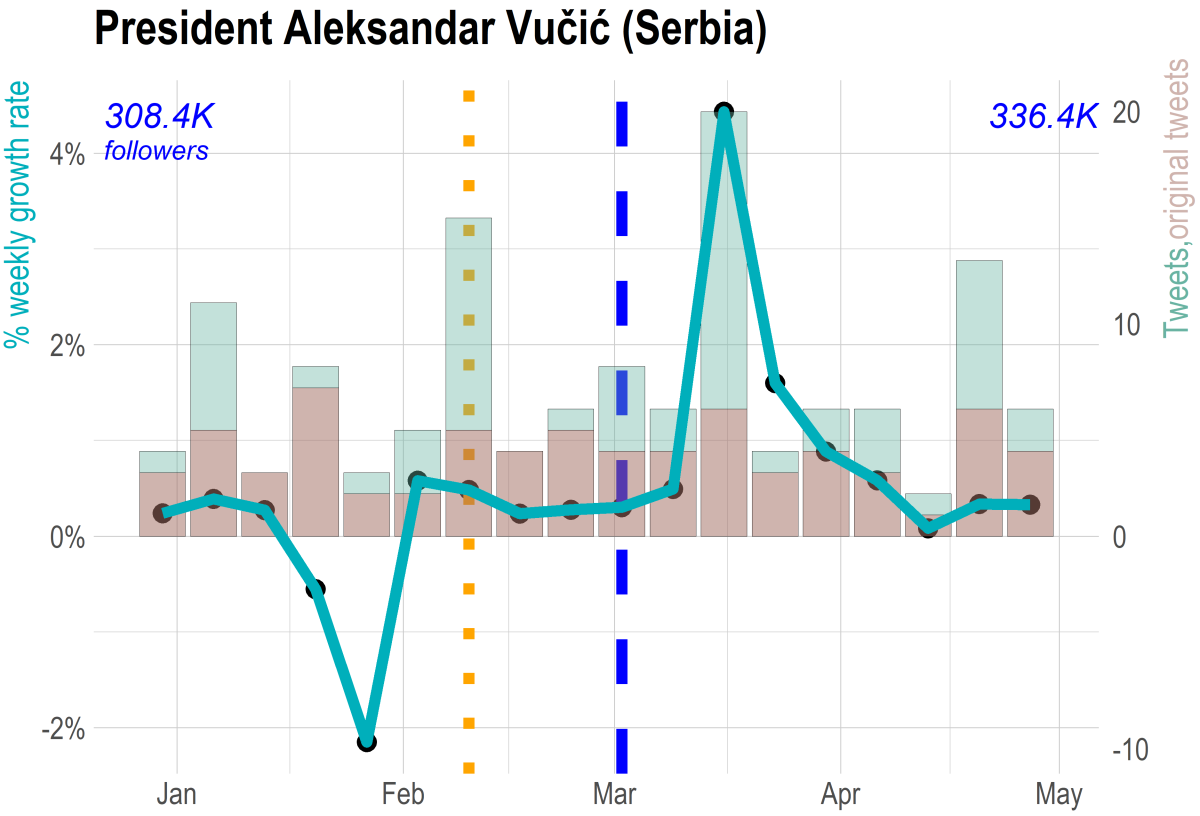

President Aleksandar Vučić (Serbia)
% weekly growth rate
Tweets, original tweets
308.4K
followers
336.4K
4%
2%
0%
-2%
20
10
0
-10
Jan
Feb
Mar
Apr
May

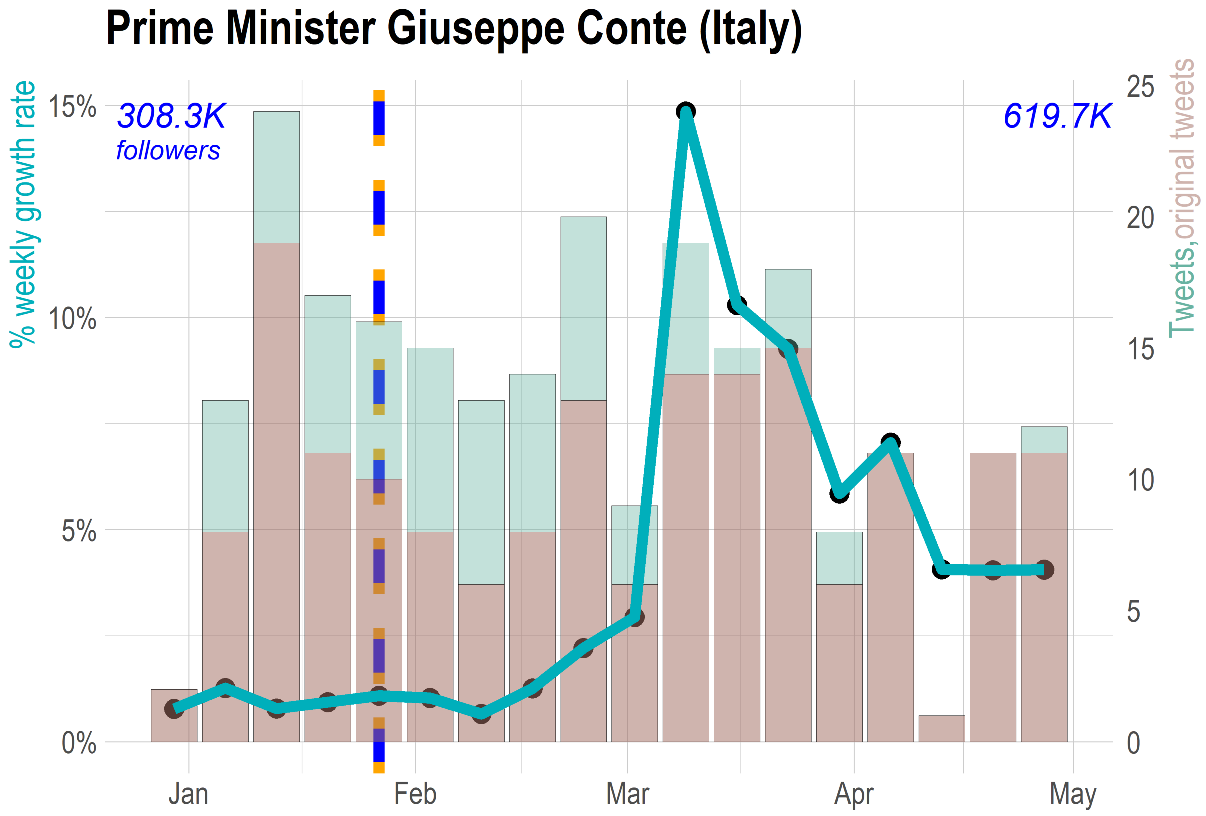
Prime Minister Giuseppe Conte (Italy)
% weekly growth rate
308.3K
followers
619.7K
Tweets, original tweets
15%
10%
5%
0%
25
20
15
10
5
0
Jan
Feb
Mar
Apr
May
