# Supplementary material for: The use of Twitter by state leaders and its impact on the public during the COVID-19 pandemic
Source: Heliyon. 2020 Nov 19;6(11):e05540. doi: 10.1016/j.heliyon.2020.e05540 (PMC7695954; doi:10.1016/j.heliyon.2020.e05540)

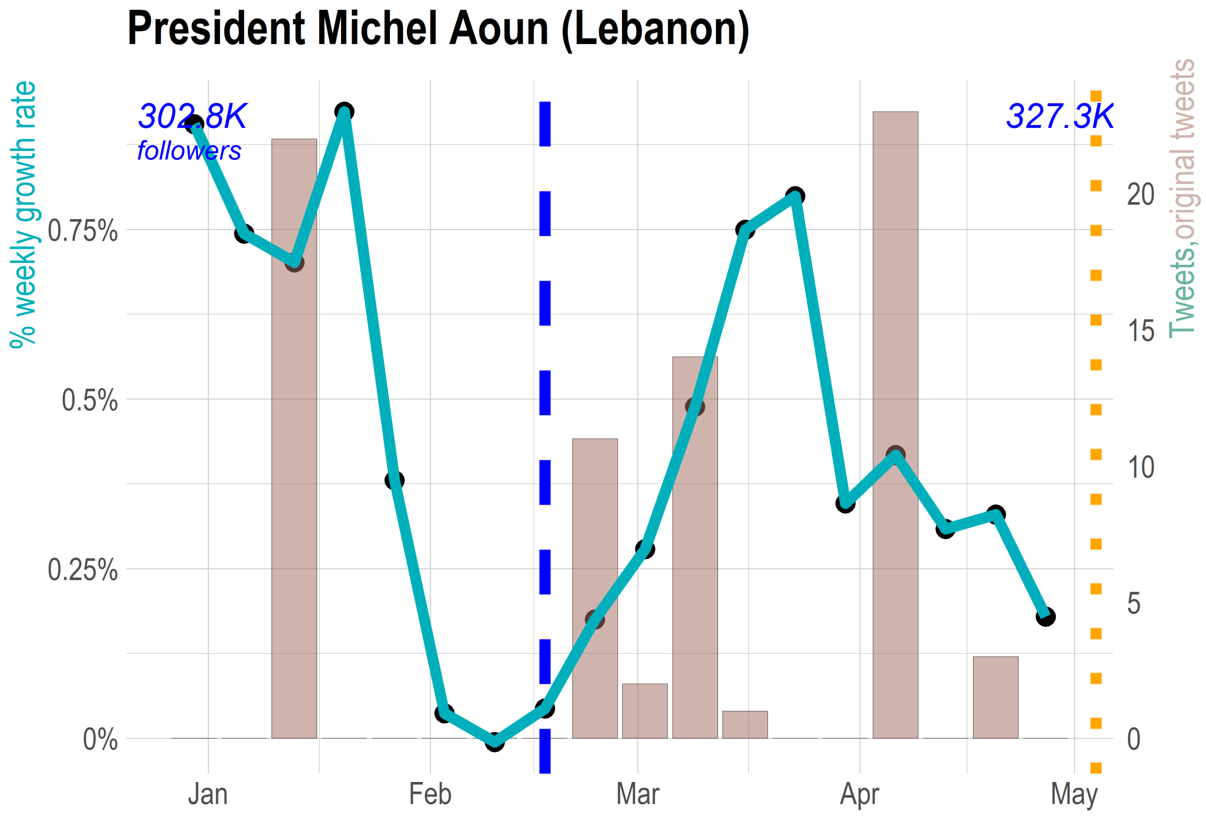

President Michel Aoun (Lebanon)
302.8K
followers
327.3K
% weekly growth rate
0.75%
0.5%
0.25%
0%
Tweets, original tweets
20
15
10
5
0
Jan
Feb
Mar
Apr
May

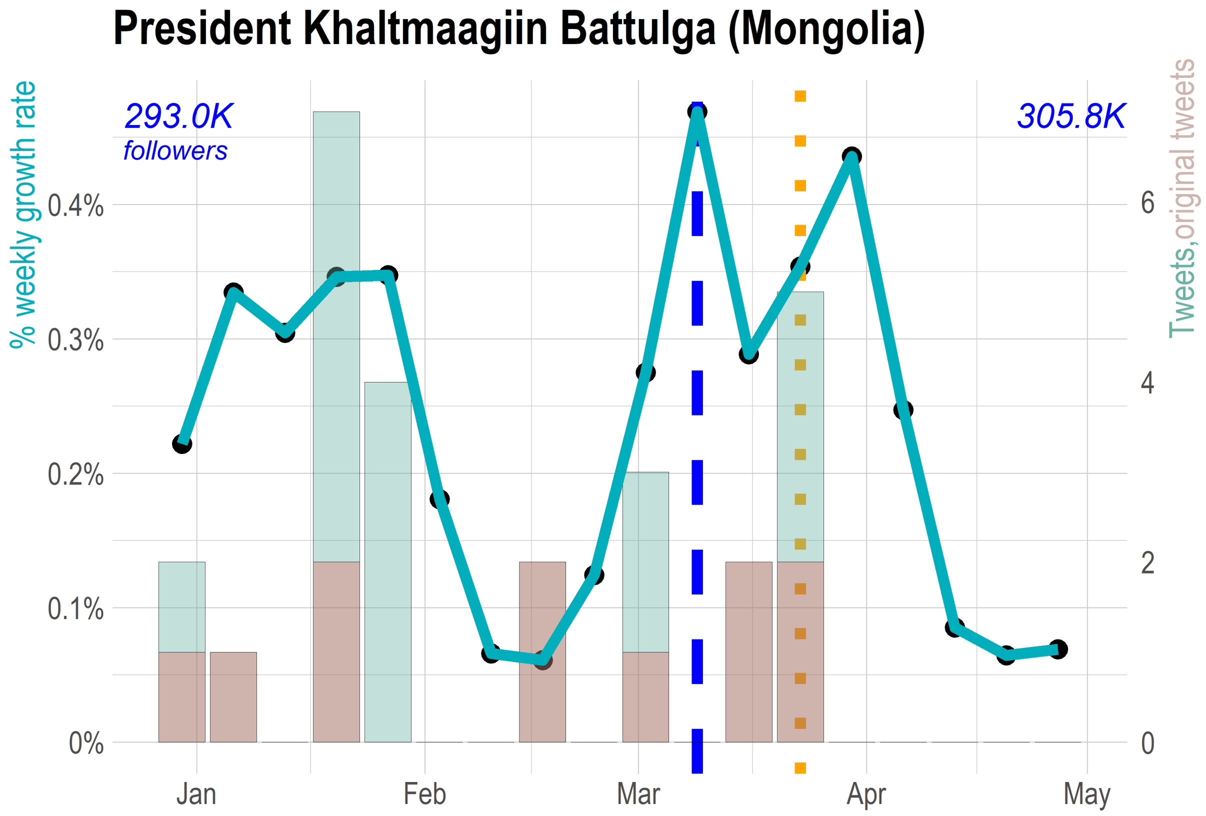
President Khaltmaagiin Battulga (Mongolia)
% weekly growth rate
Tweets, original tweets
293.0K
followers
305.8K
0.4%
0.3%
0.2%
0.1%
0%
6
4
2
0
Jan
Feb
Mar
Apr
May

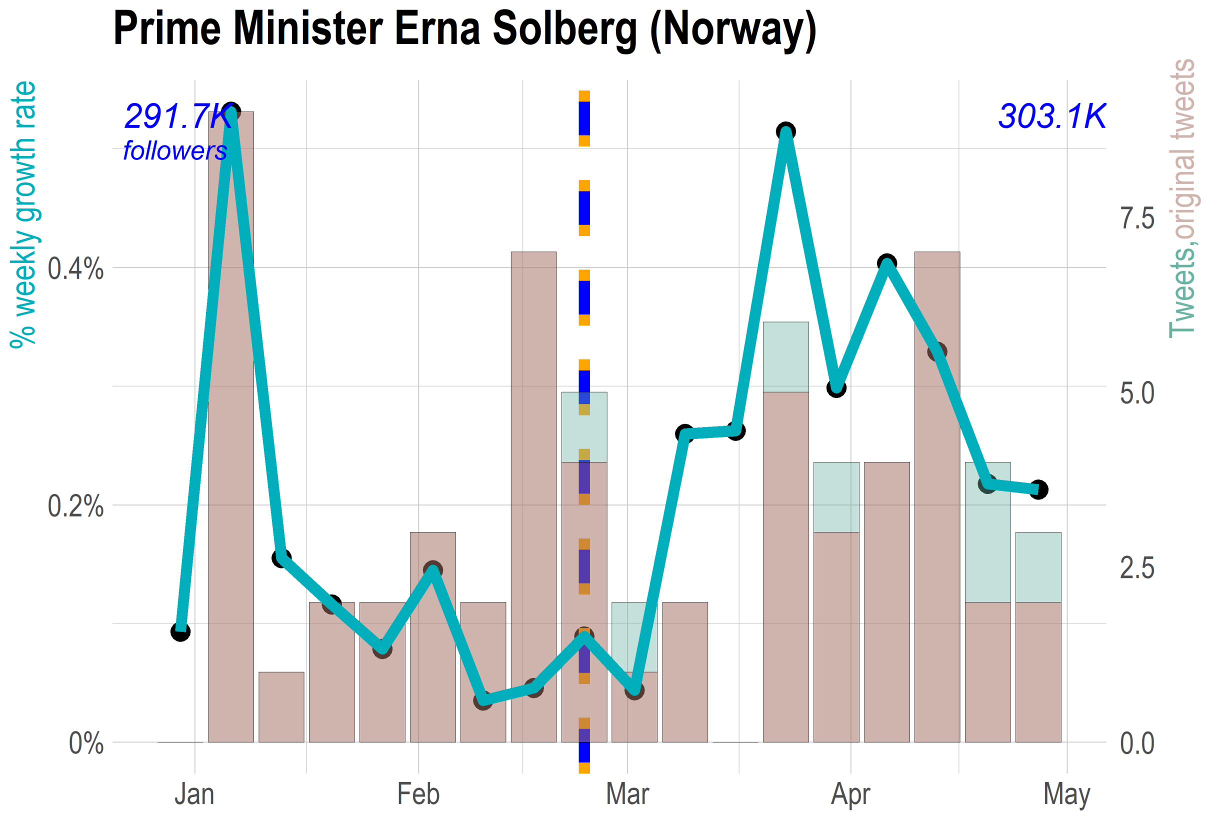

Prime Minister Erna Solberg (Norway)
291.7K
followers
303.1K
% weekly growth rate
Tweets, original tweets
0.4%
0.2%
0%
7.5
5.0
2.5
0.0
Jan
Feb
Mar
Apr
May

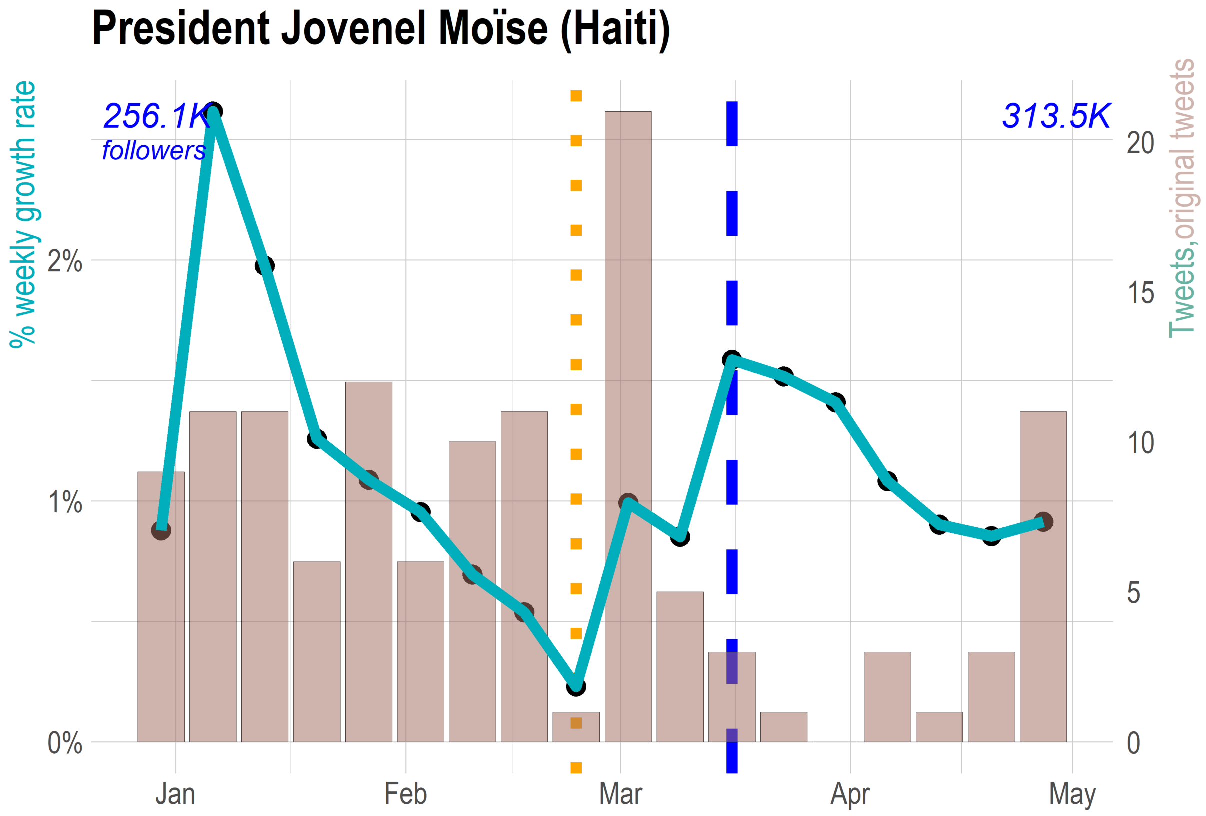

President Jovenel Moïse (Haiti)
256.1K
followers
313.5K
% weekly growth rate
Tweets, original tweets
2%
1%
0%
20
15
10
5
0
Jan
Feb
Mar
Apr
May

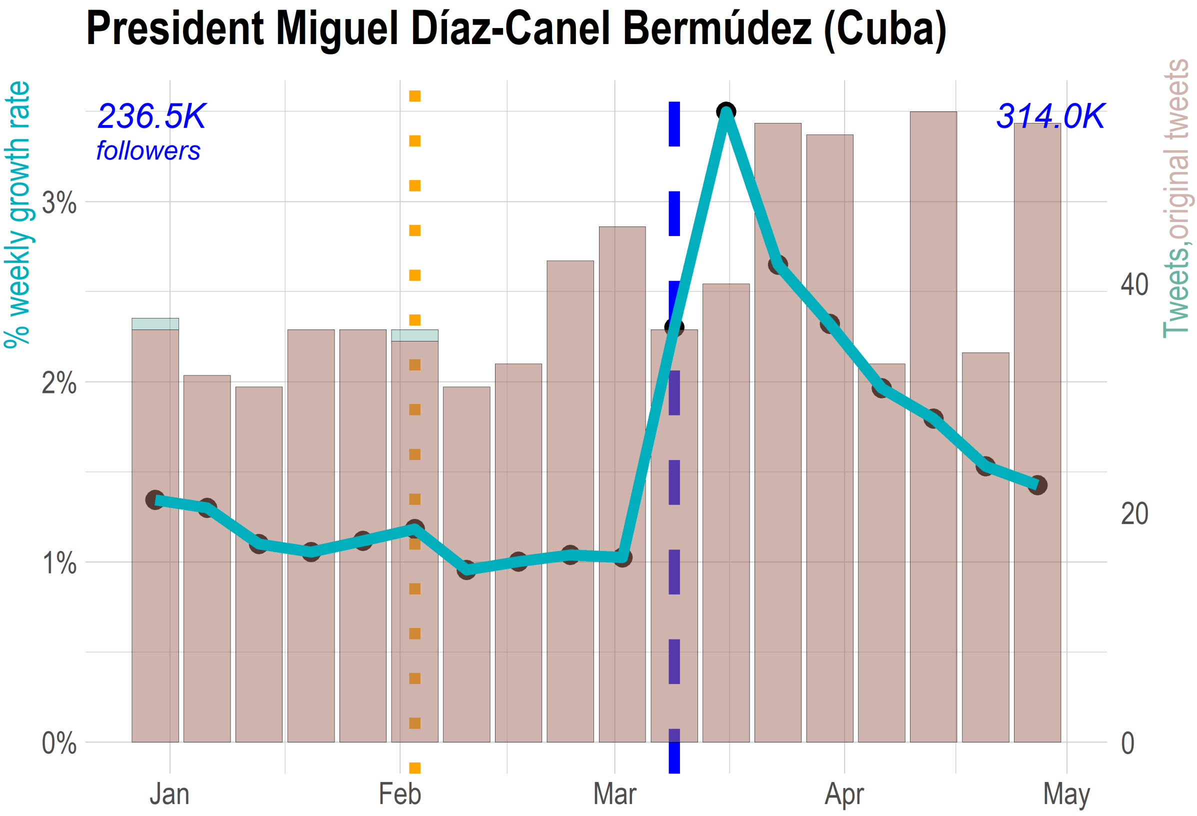
President Miguel Díaz-Canel Bermúdez (Cuba)
% weekly growth rate
Tweets, original tweets
236.5K
followers
314.0K
3%
2%
1%
0%
40
20
0
Jan
Feb
Mar
Apr
May

Prime Minister Leo Varadkar (Ireland)

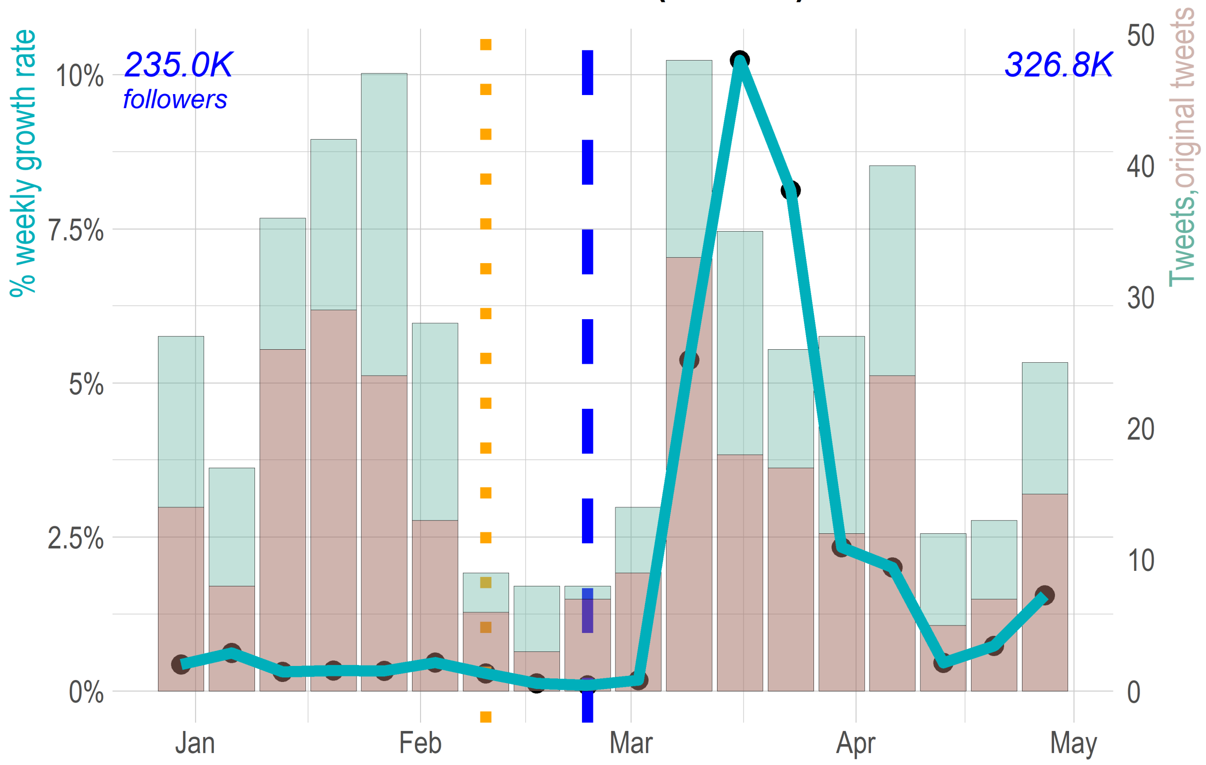

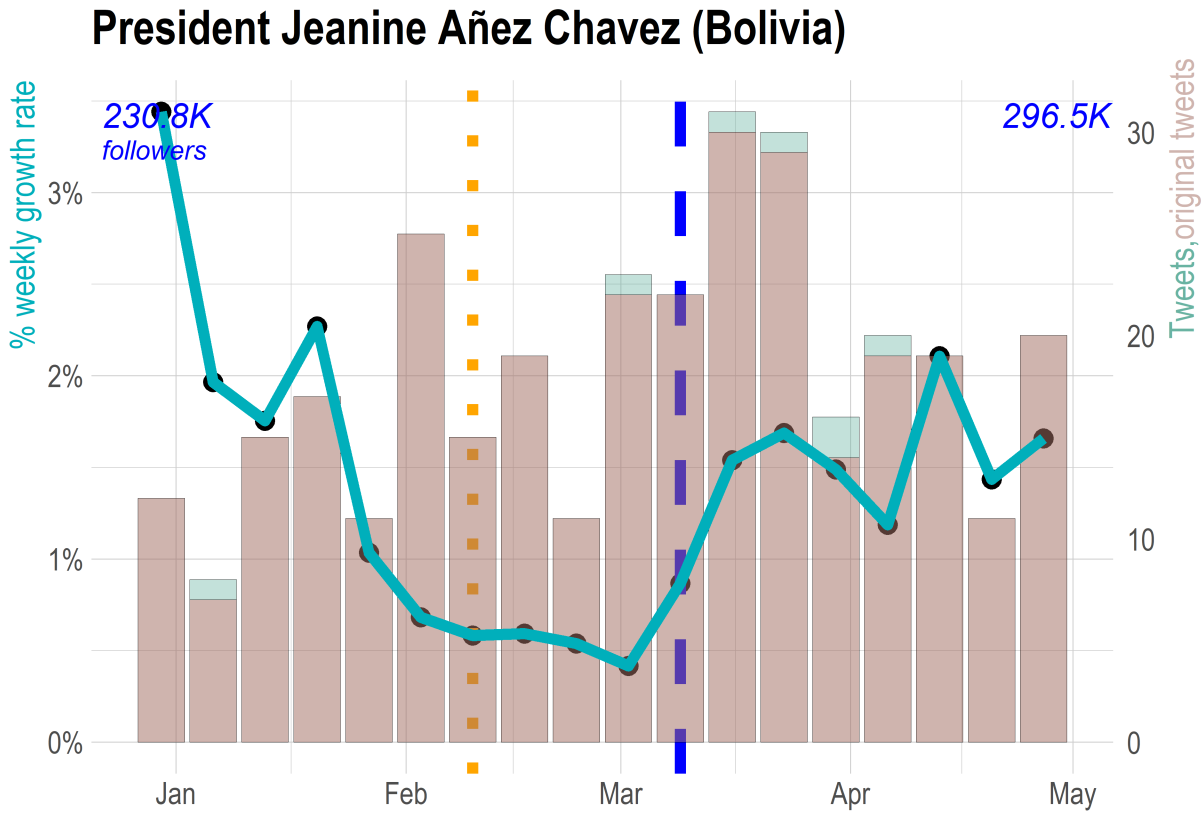
President Jeanine Añez Chavez (Bolivia)
% weekly growth rate
Tweets, original tweets
230.8K
followers
296.5K
0%
1%
2%
3%
0
10
20
30
Jan
Feb
Mar
Apr
May

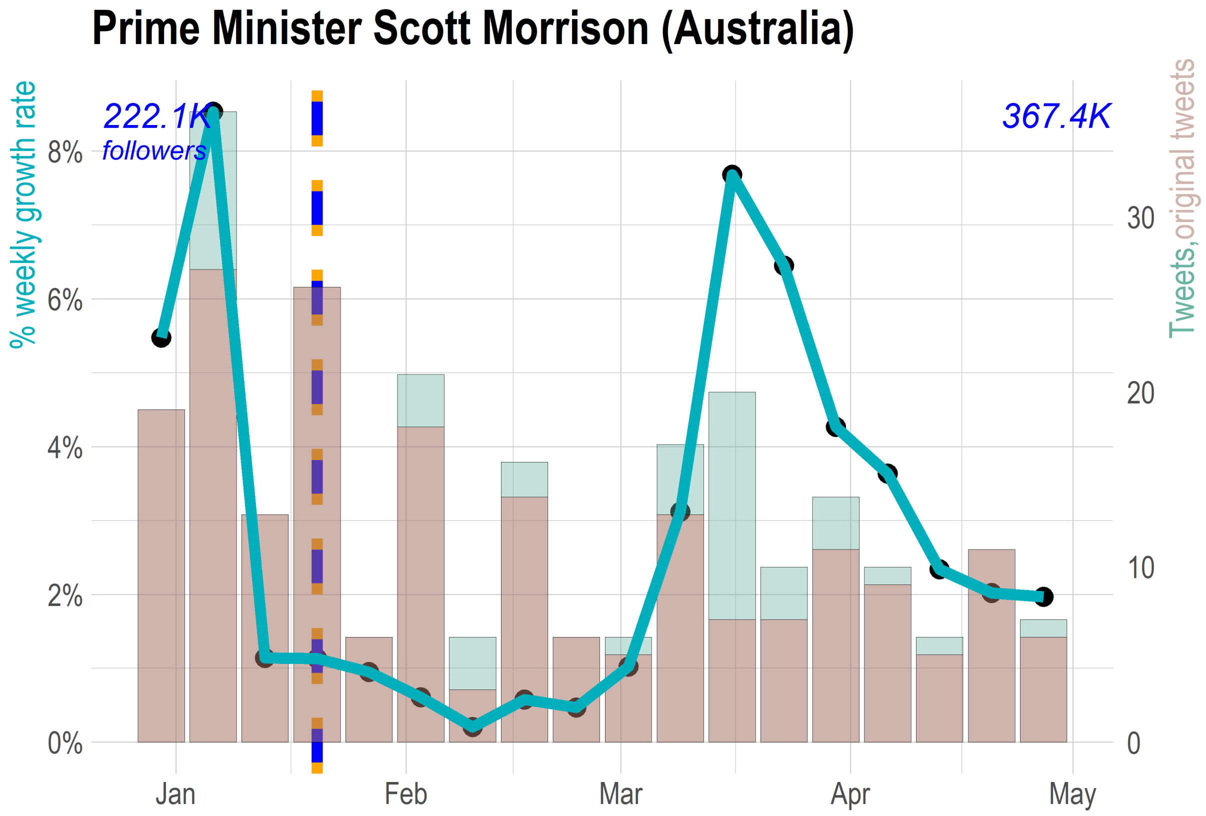
Prime Minister Scott Morrison (Australia)
% weekly growth rate
Tweets, original tweets
222.1K
followers
367.4K
8%
6%
4%
2%
0%
30
20
10
0
Jan
Feb
Mar
Apr
May

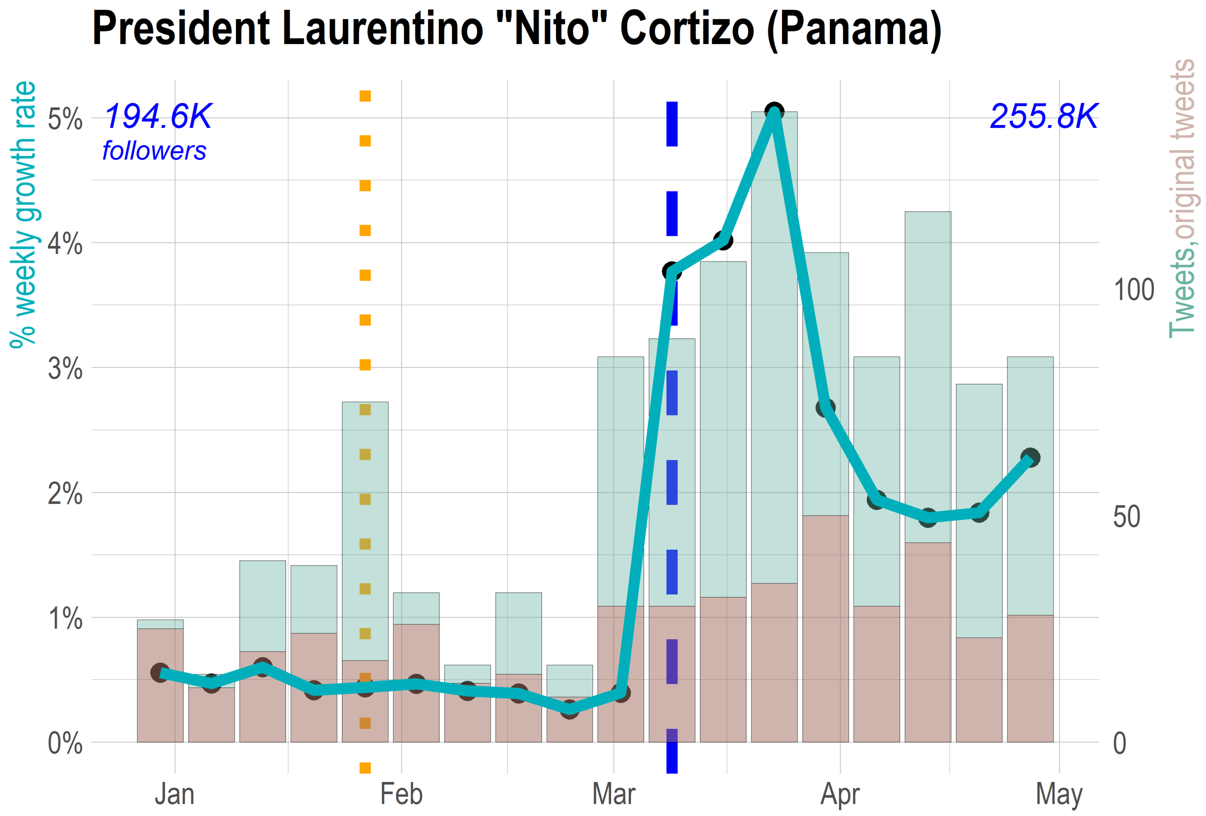

President Laurentino "Nito" Cortizo (Panama)
% weekly growth rate
194.6K
followers
255.8K
Tweets, original tweets
5%
4%
3%
2%
1%
0%
100
50
0
Jan
Feb
Mar
Apr
May

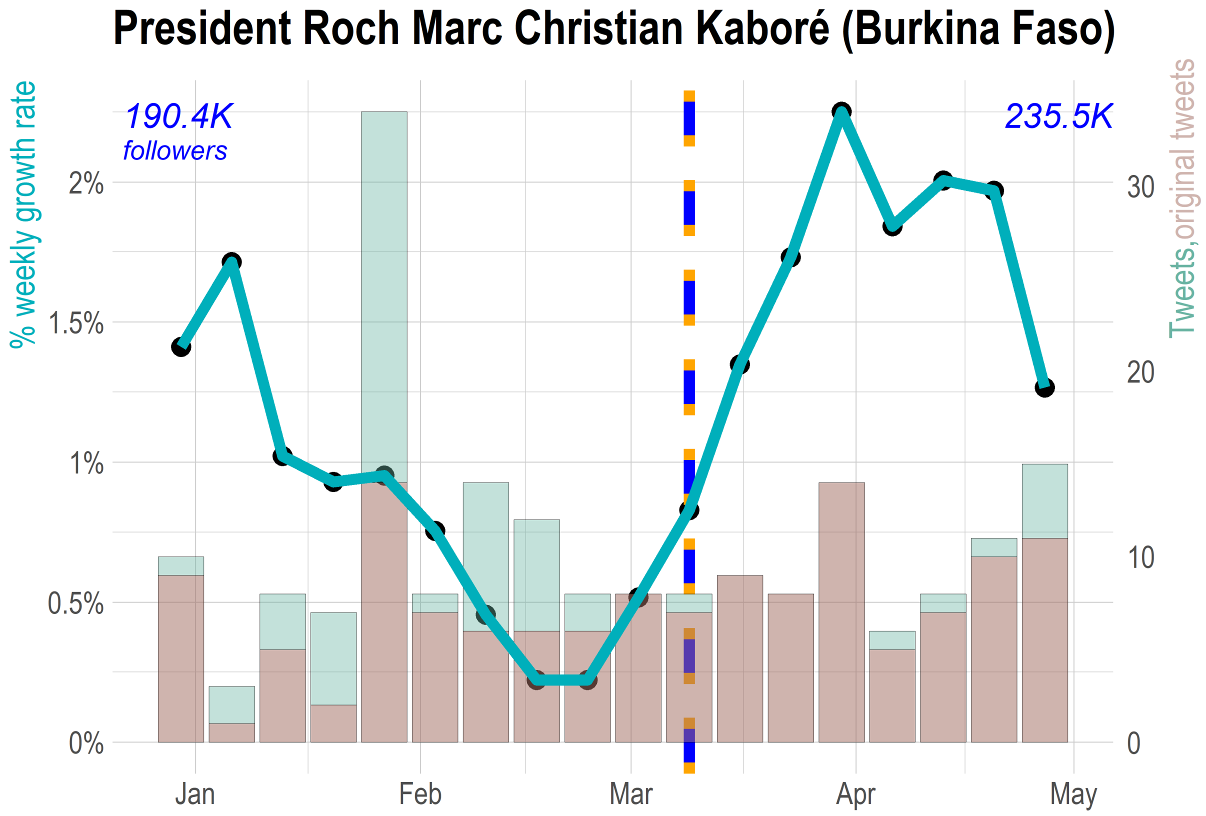

President Roch Marc Christian Kaboré (Burkina Faso)
% weekly growth rate
Tweets, original tweets
190.4K
followers
235.5K
2%
1.5%
1%
0.5%
0%
30
20
10
0
Jan
Feb
Mar
Apr
May

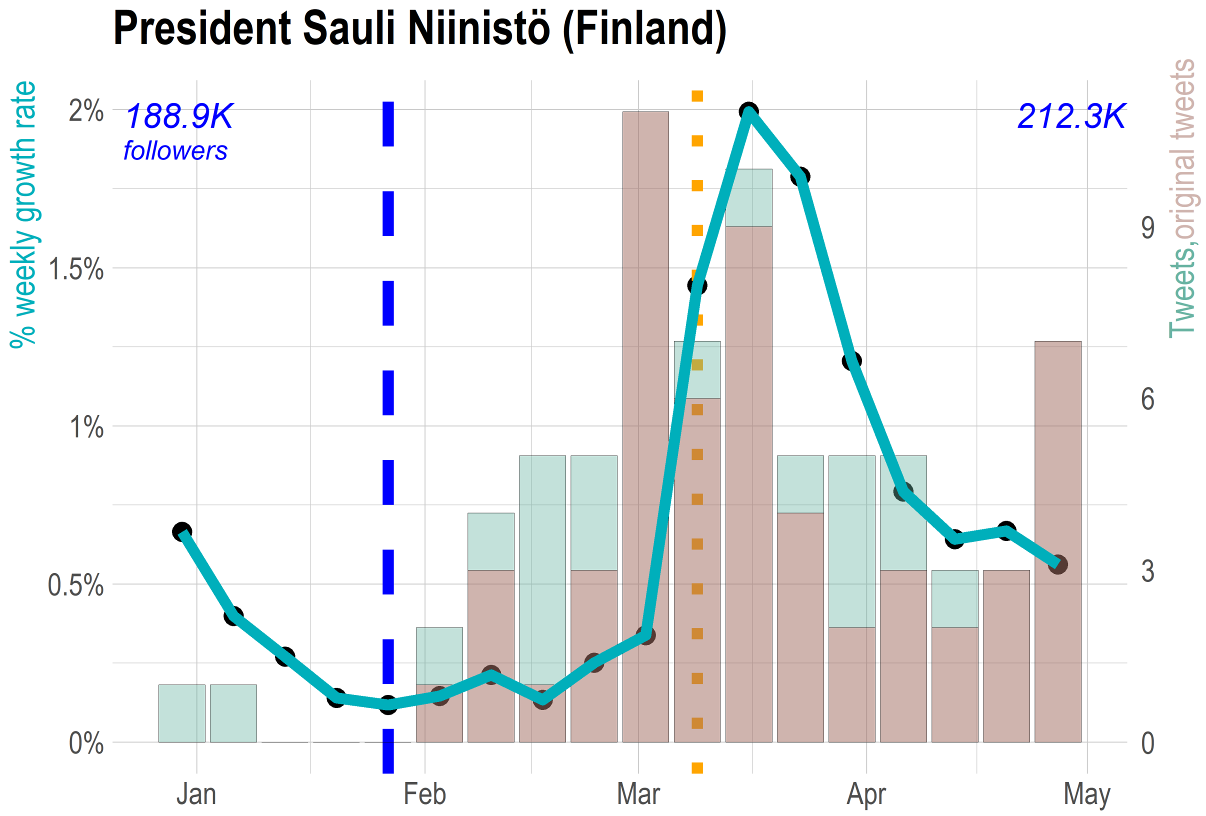

President Sauli Niinistö (Finland)
% weekly growth rate
Tweets, original tweets
188.9K
followers
212.3K
2%
1.5%
1%
0.5%
0%
9
6
3
0
Jan
Feb
Mar
Apr
May

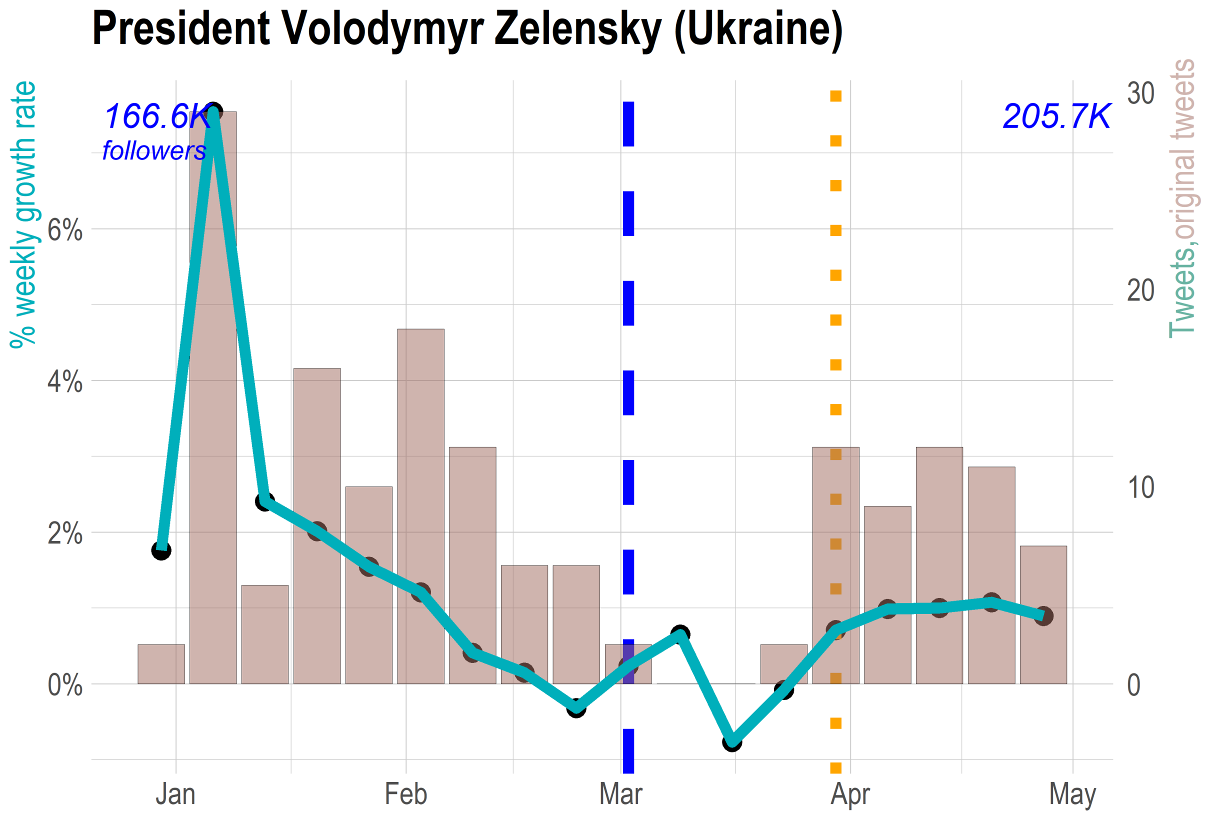

President Volodymyr Zelensky (Ukraine)
166.6K
followers
205.7K
% weekly growth rate
6%
4%
2%
0%
Tweets, original tweets
30
20
10
0
Jan
Feb
Mar
Apr
May

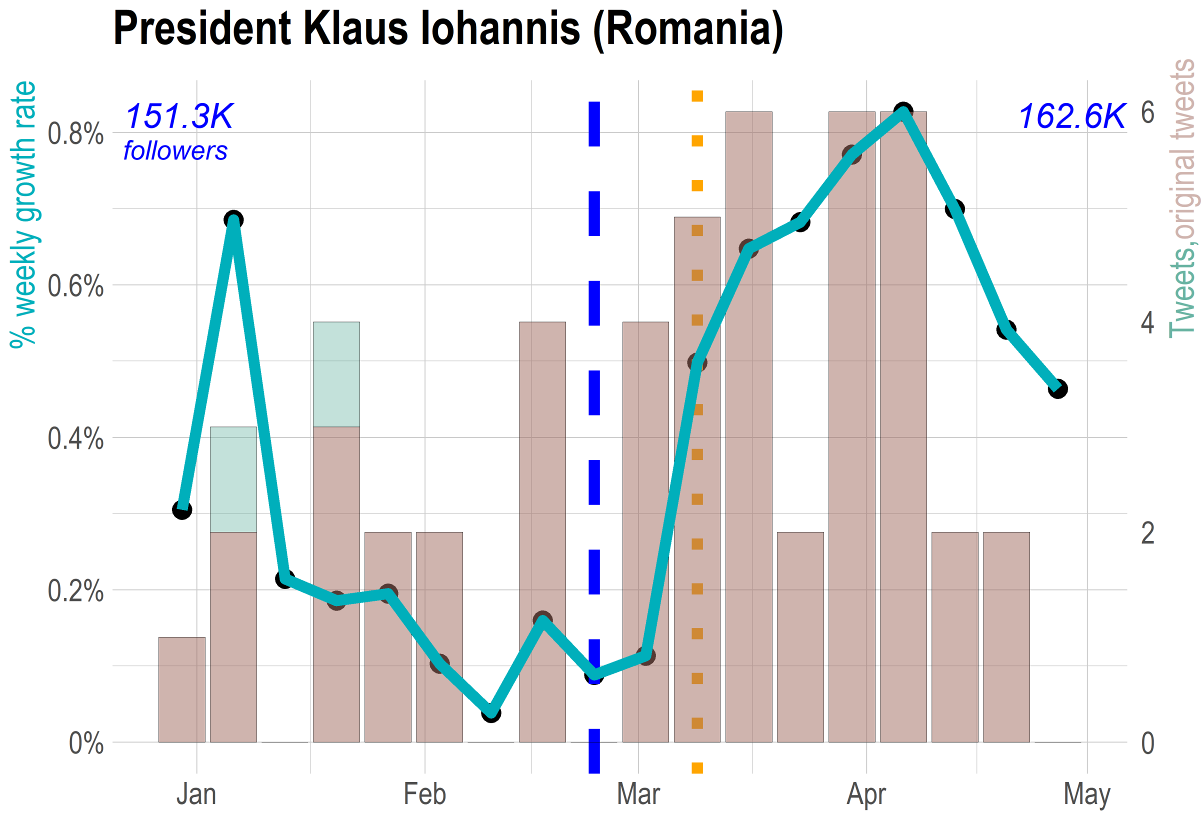
President Klaus Iohannis (Romania)
% weekly growth rate
Tweets, original tweets
151.3K
followers
162.6K
0.8%
0.6%
0.4%
0.2%
0%
6
4
2
0
Jan
Feb
Mar
Apr
May

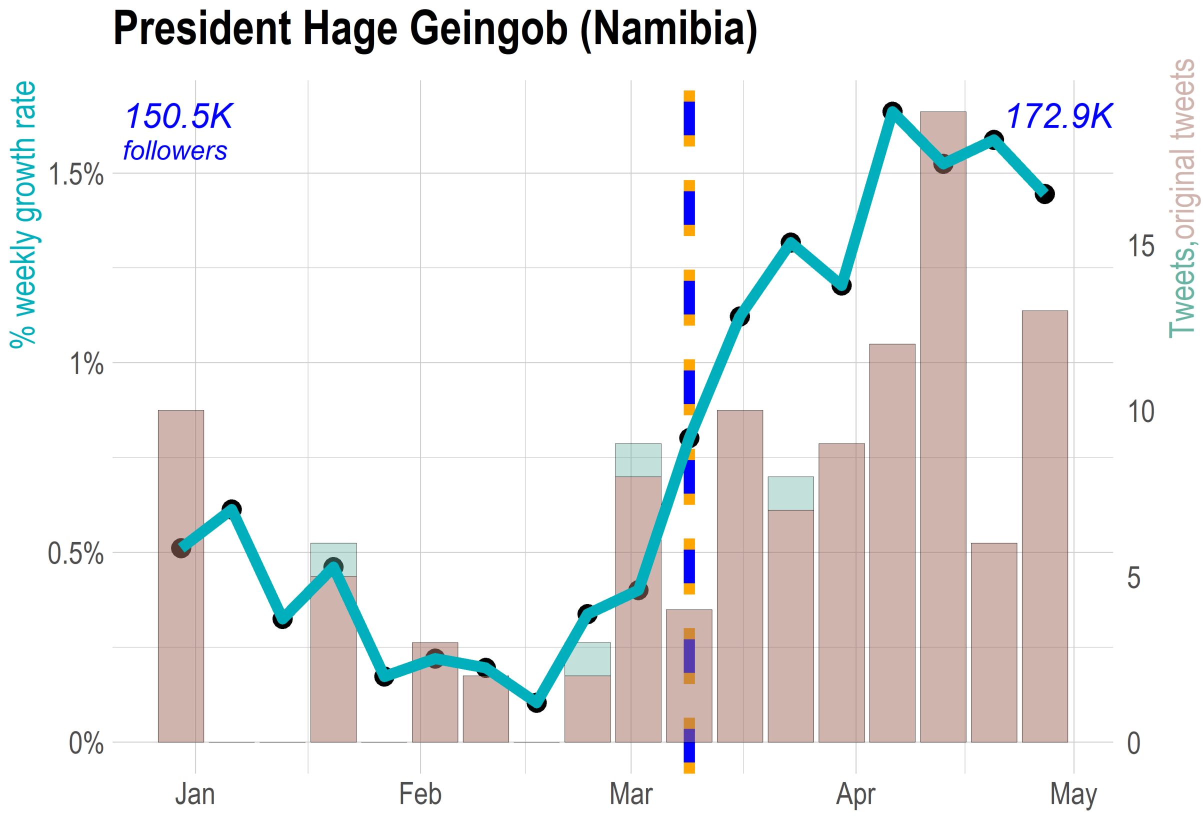
President Hage Geingob (Namibia)
% weekly growth rate
Tweets, original tweets
150.5K
followers
172.9K
1.5%
1%
0.5%
0%
15
10
5
0
Jan
Feb
Mar
Apr
May

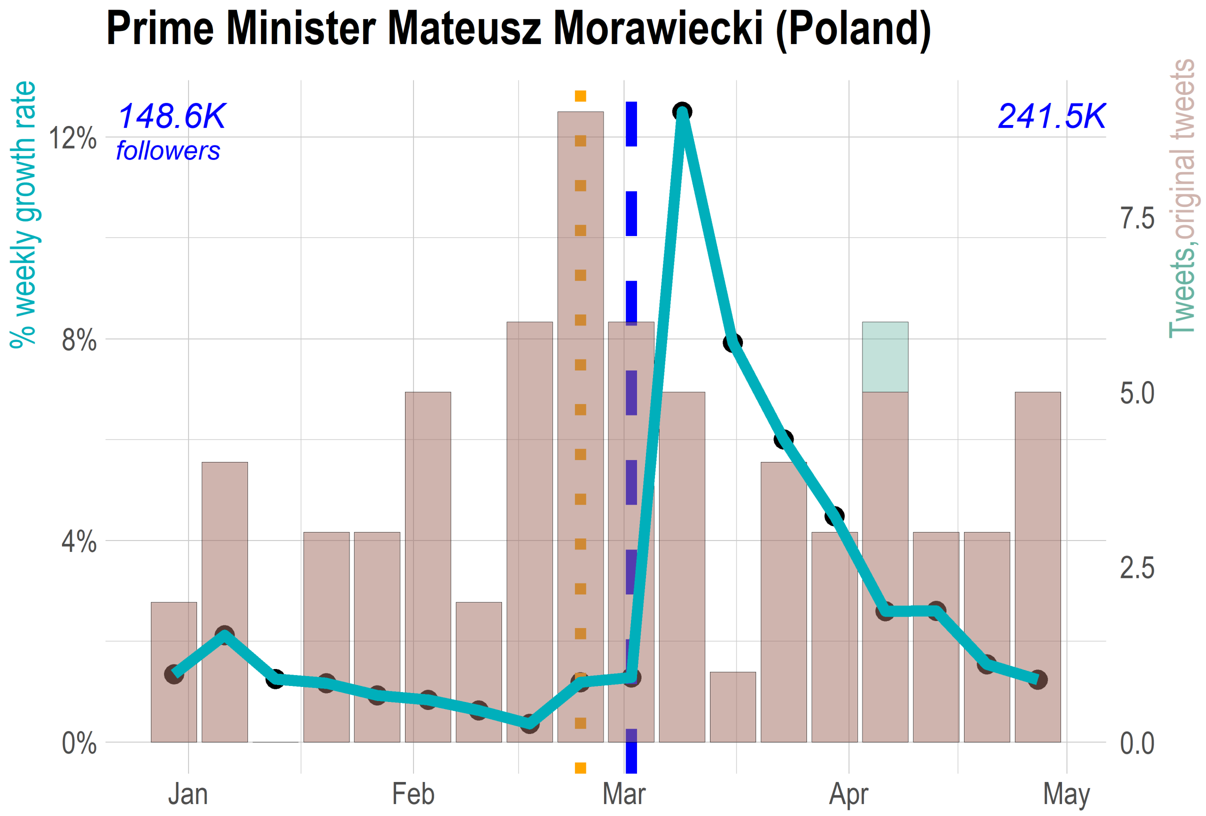

Prime Minister Mateusz Morawiecki (Poland)
% weekly growth rate
Tweets, original tweets
148.6K
followers
241.5K
12%
8%
4%
0%
7.5
5.0
2.5
0.0
Jan
Feb
Mar
Apr
May

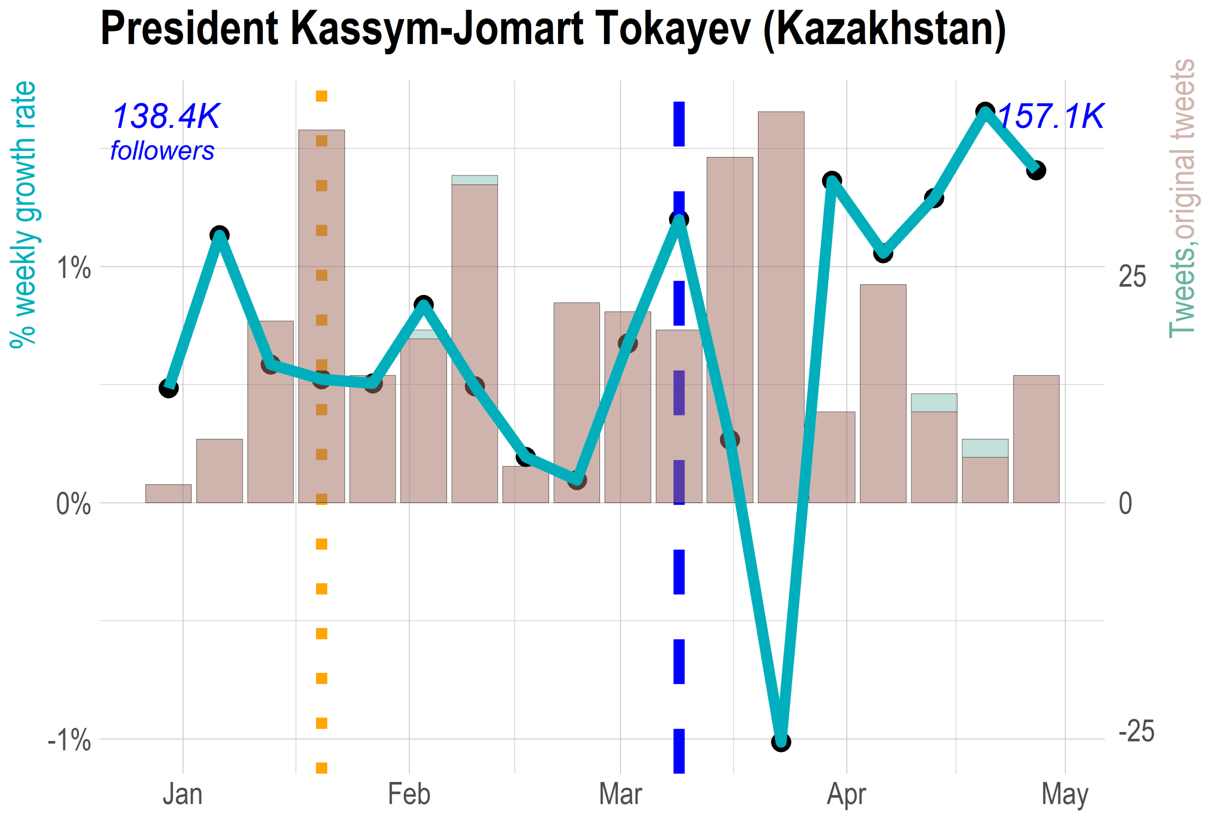

President Kassym-Jomart Tokayev (Kazakhstan)
% weekly growth rate
Tweets, original tweets
138.4K
followers
157.1K
1%
0%
-1%
25
0
-25
Jan
Feb
Mar
Apr
May

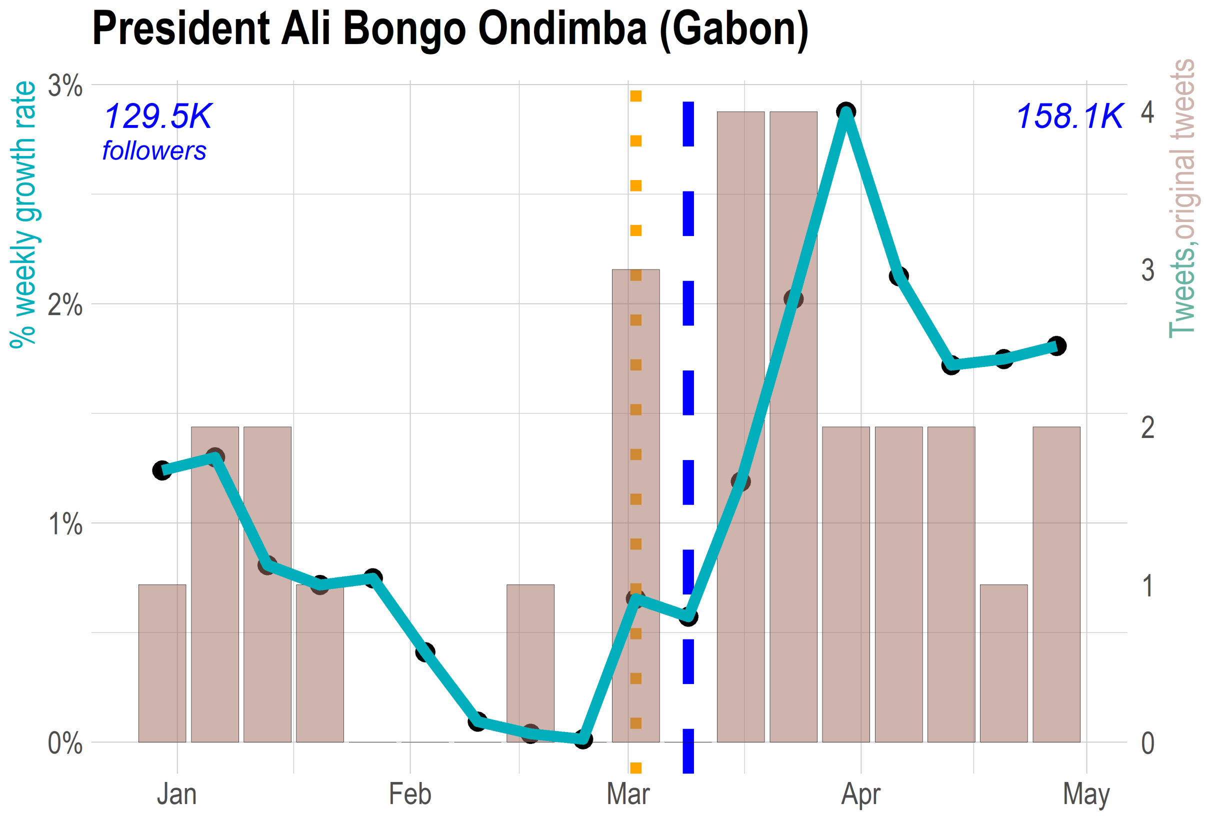

President Ali Bongo Ondimba (Gabon)
% weekly growth rate
Tweets, original tweets
129.5K
followers
158.1K
3%
2%
1%
0%
4
3
2
1
0
Jan
Feb
Mar
Apr
May

President Gotabaya Rajapaksa (Sri Lanka)

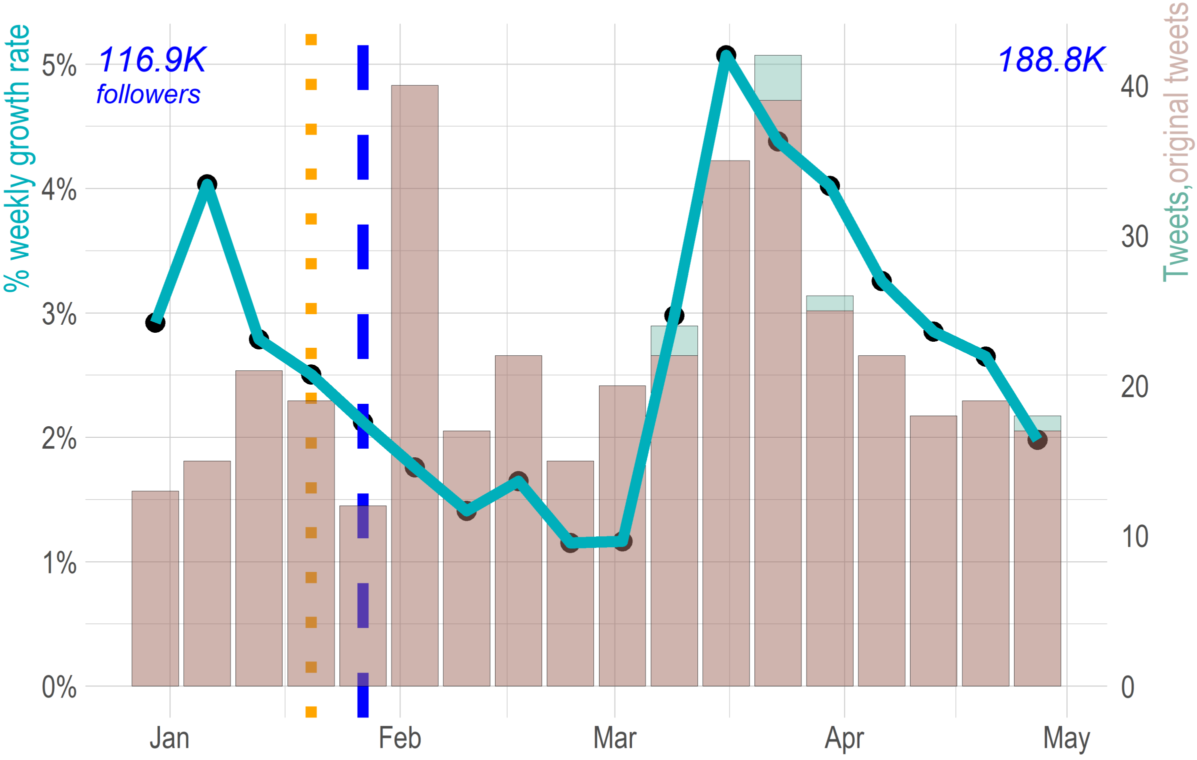

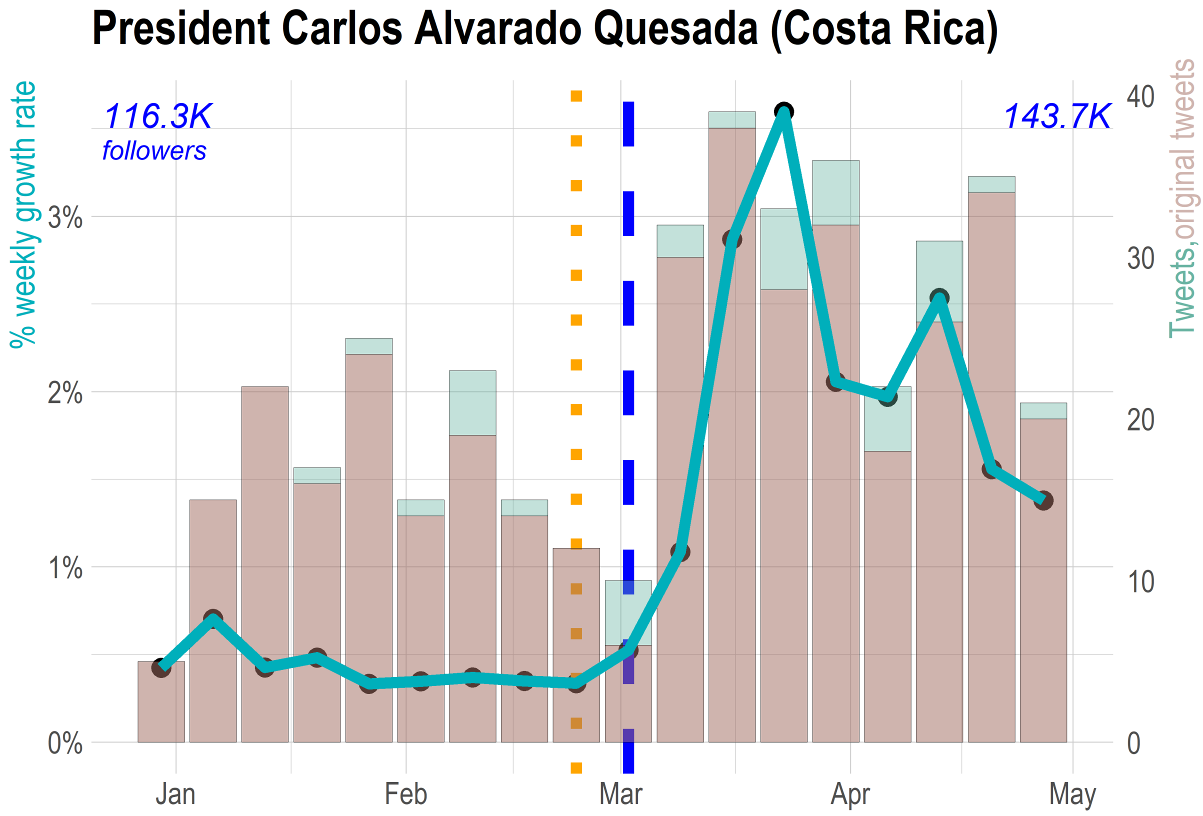
President Carlos Alvarado Quesada (Costa Rica)
116.3K
followers
143.7K
% weekly growth rate
Tweets, original tweets
0%
1%
2%
3%
0
10
20
30
40
Jan
Feb
Mar
Apr
May

Prime Minister Abiy Ahmed (Ethiopia)

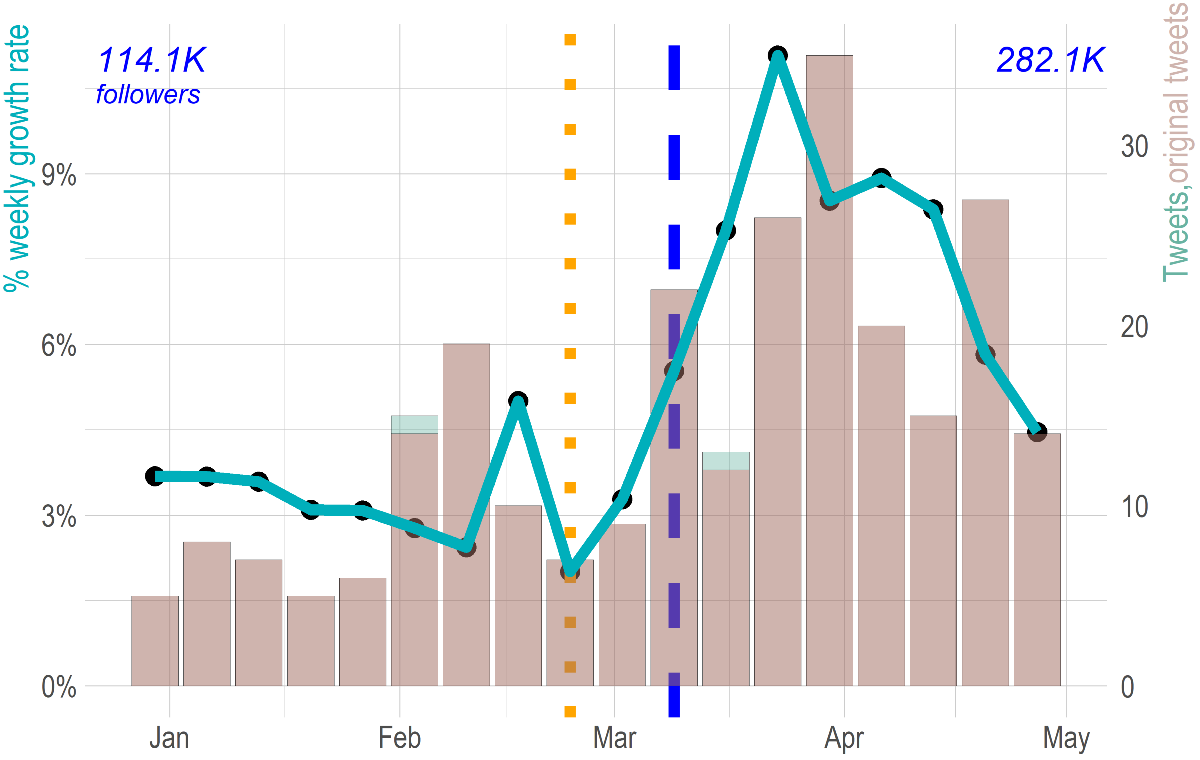

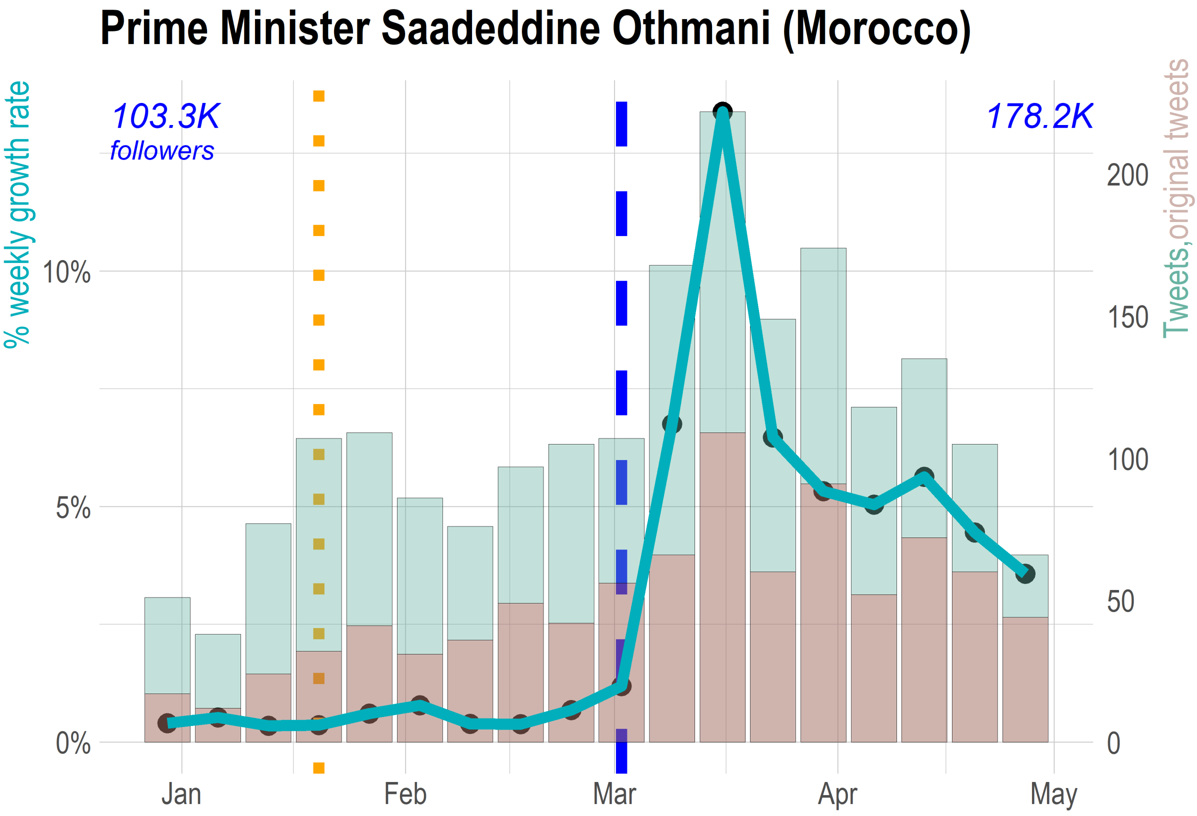

Prime Minister Saadeddine Othmani (Morocco)
103.3K
followers
178.2K
% weekly growth rate
Tweets,original tweets
10%
5%
0%
200
150
100
50
0
Jan
Feb
Mar
Apr
May

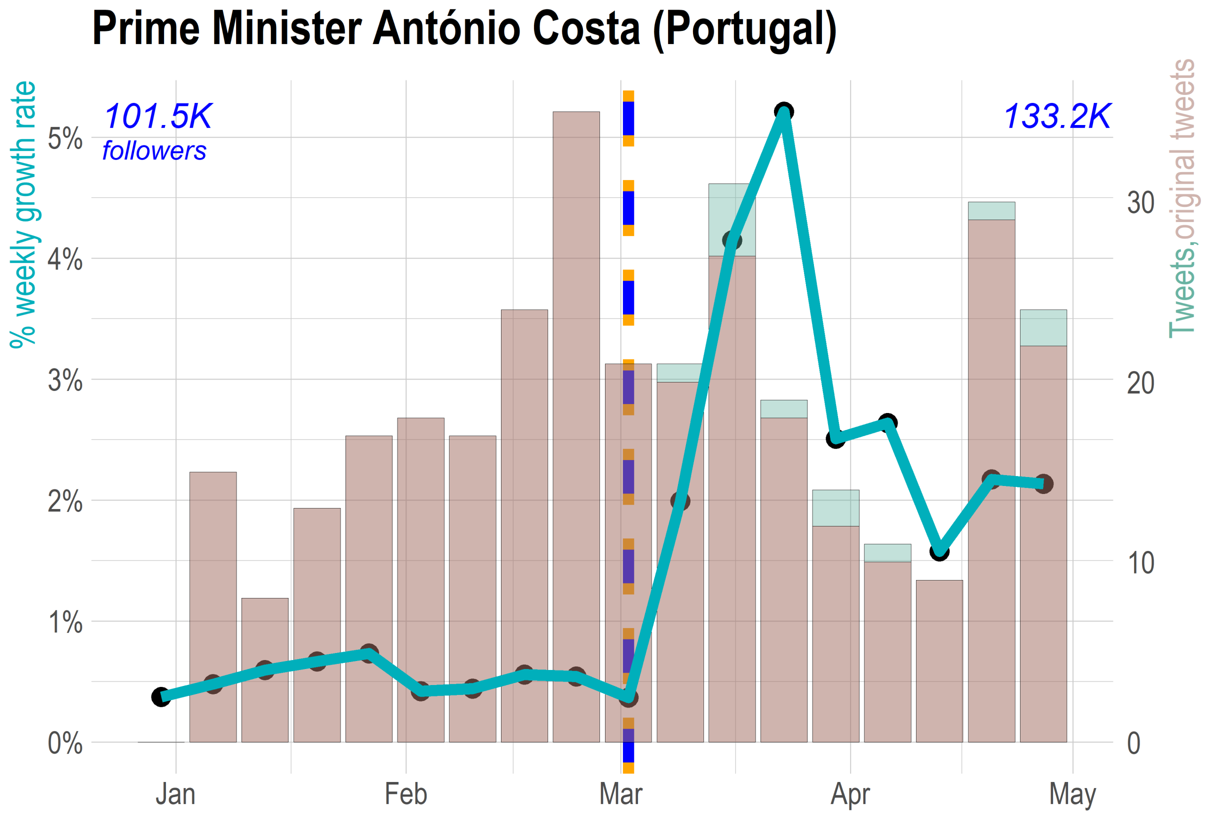

Prime Minister António Costa (Portugal)
% weekly growth rate
Tweets, original tweets
101.5K
followers
133.2K
5%
4%
3%
2%
1%
0%
30
20
10
0
Jan
Feb
Mar
Apr
May

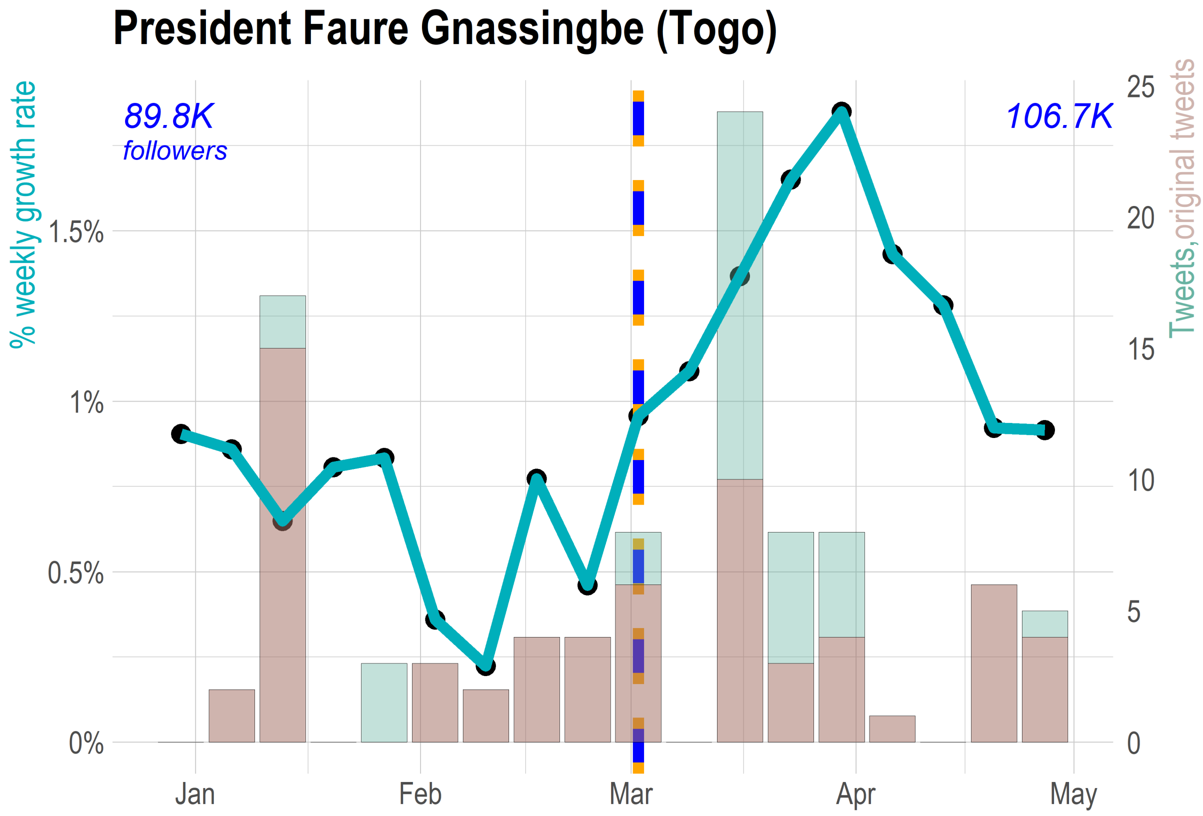

President Faure Gnassingbe (Togo)
89.8K
followers
106.7K
% weekly growth rate
1.5%
1%
0.5%
0%
Tweets, original tweets
25
20
15
10
5
0
Jan
Feb
Mar
Apr
May

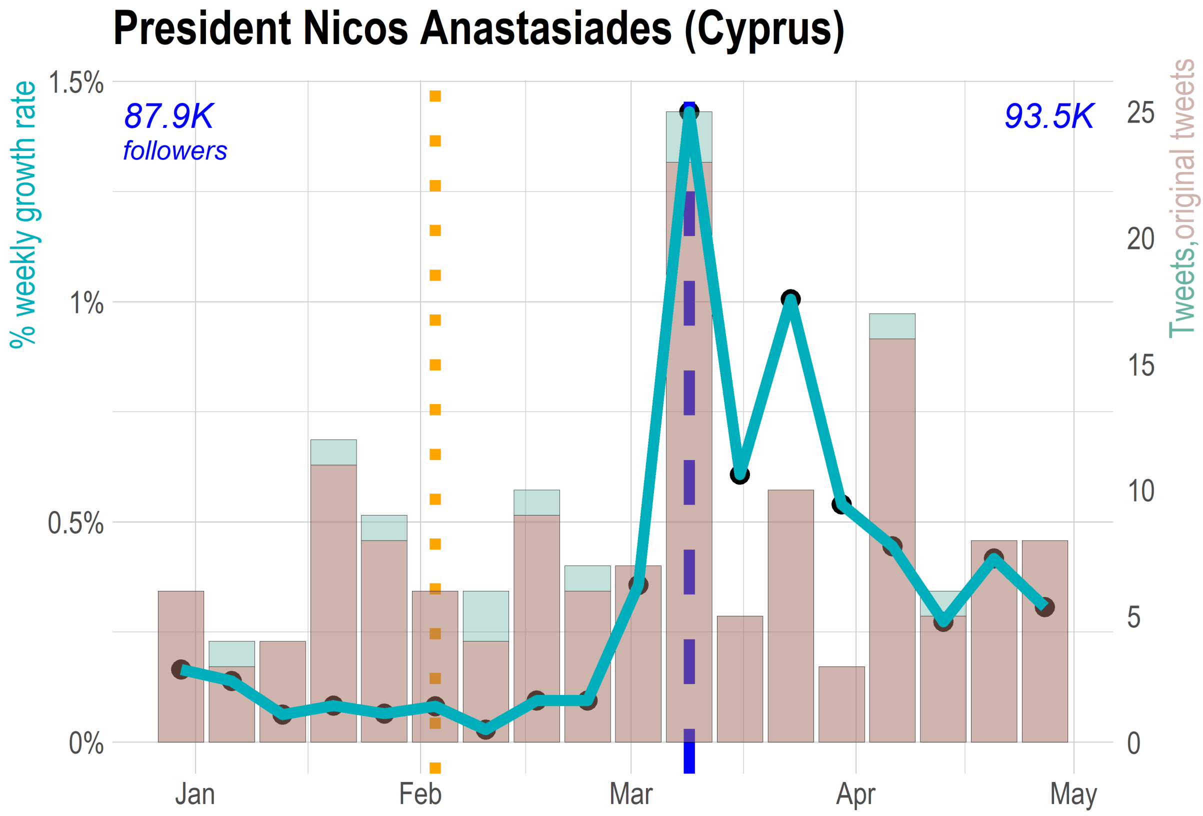

President Nicos Anastasiades (Cyprus)
% weekly growth rate
Tweets, original tweets
87.9K
followers
93.5K
1.5%
1%
0.5%
0%
25
20
15
10
5
0
Jan
Feb
Mar
Apr
May

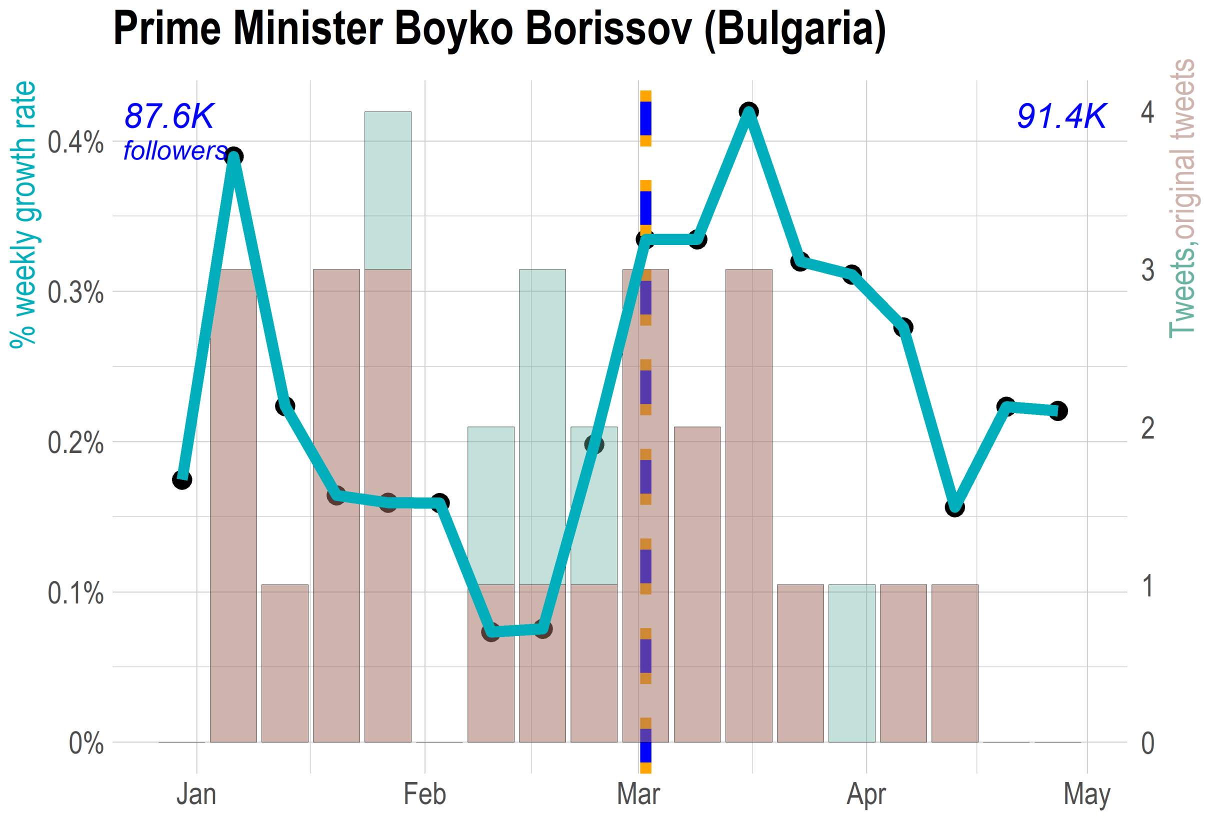

Prime Minister Boyko Borissov (Bulgaria)
% weekly growth rate
Tweets, original tweets
87.6K
followers
91.4K
0.4%
0.3%
0.2%
0.1%
0%
4
3
2
1
0
Jan
Feb
Mar
Apr
May

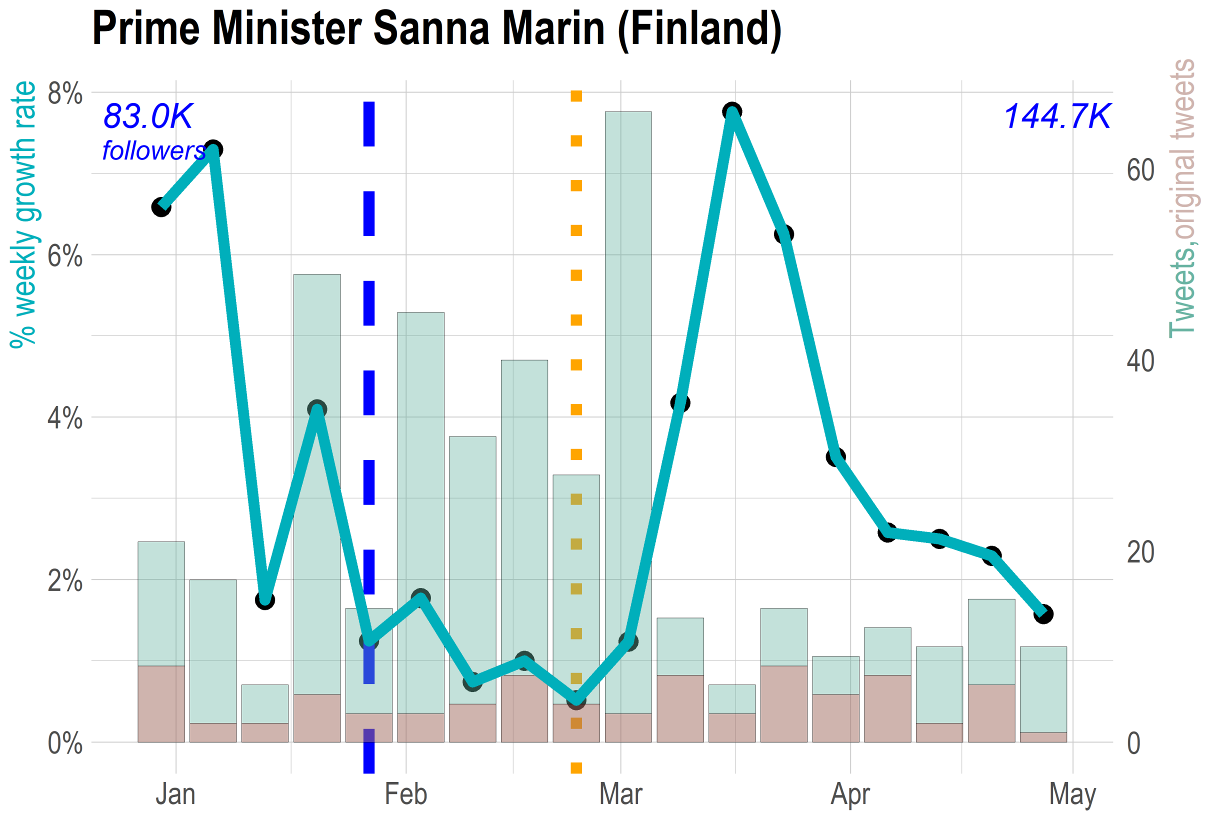

Prime Minister Sanna Marin (Finland)
% weekly growth rate
Tweets, original tweets
83.0K
followers
144.7K
8%
6%
4%
2%
0%
60
40
20
0
Jan
Feb
Mar
Apr
May

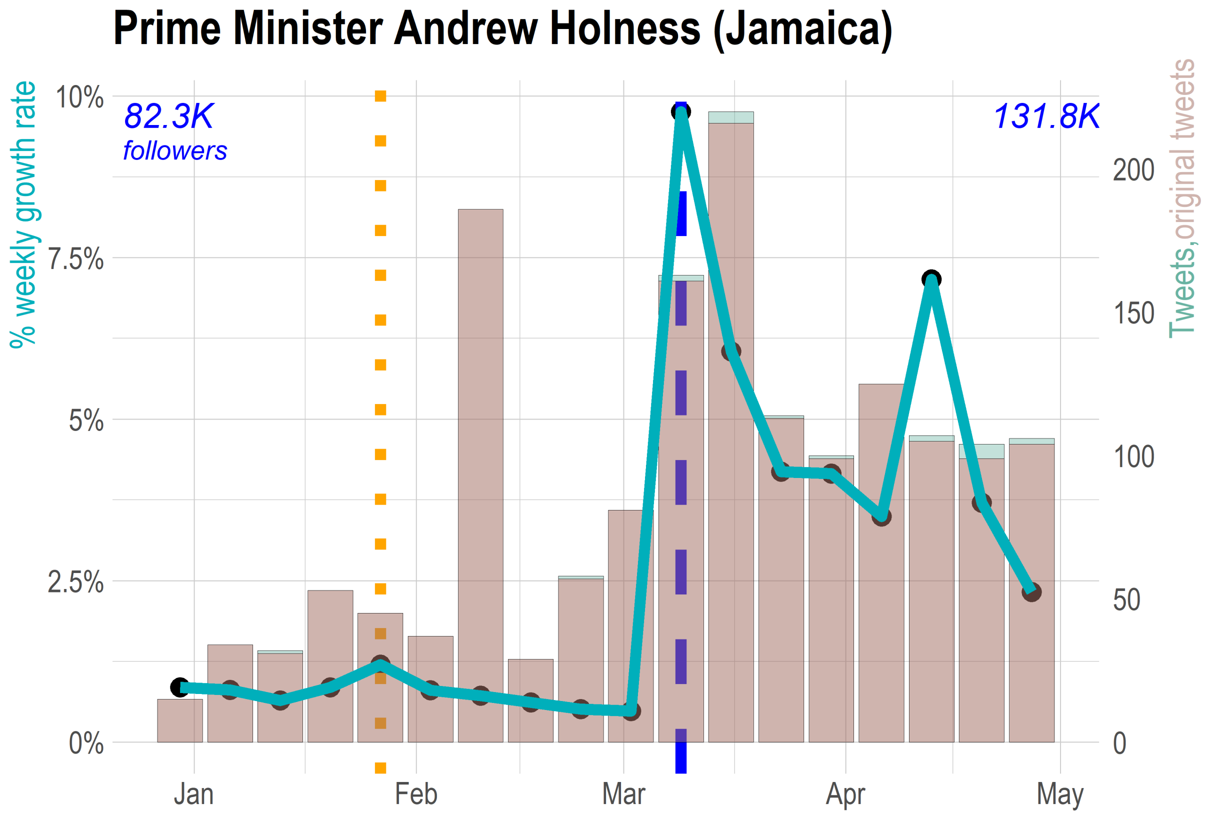

Prime Minister Andrew Holness (Jamaica)
% weekly growth rate
Tweets, original tweets
82.3K
followers
131.8K
10%
7.5%
5%
2.5%
0%
200
150
100
50
0
Jan
Feb
Mar
Apr
May

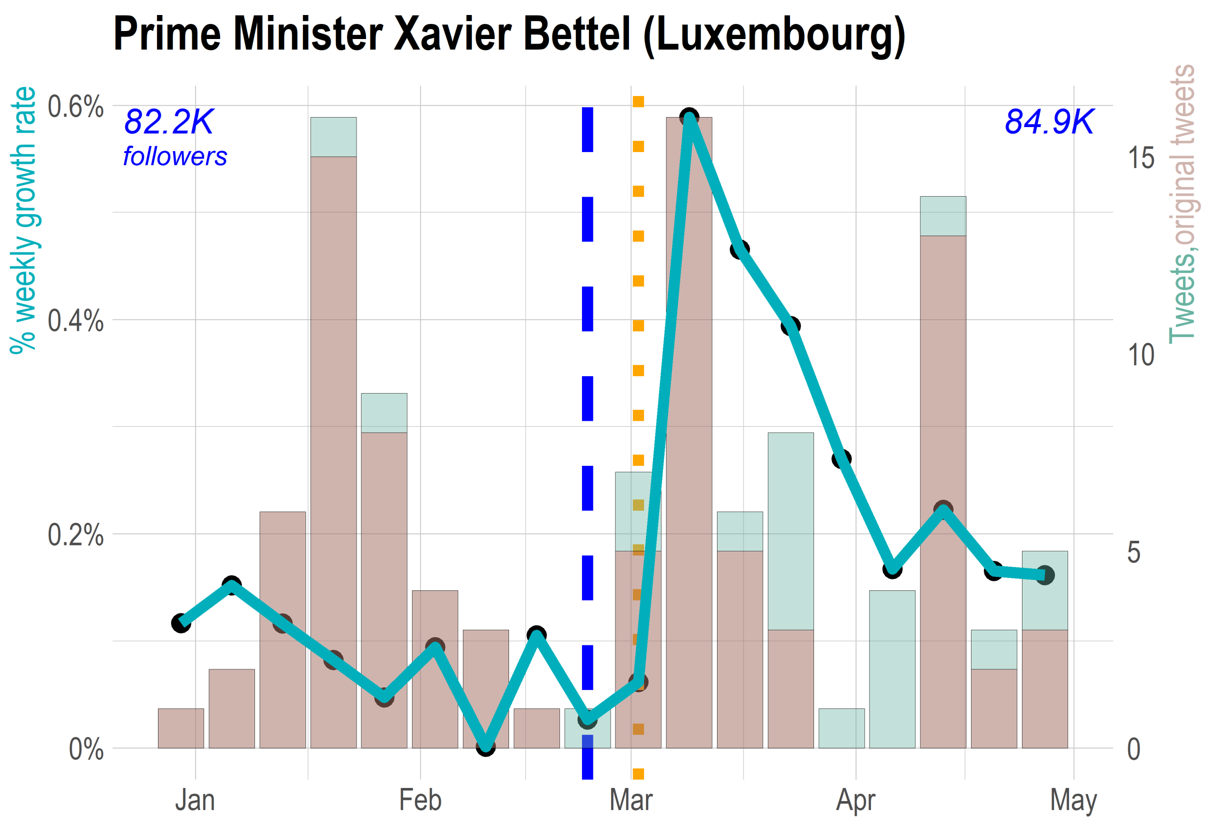

Prime Minister Xavier Bettel (Luxembourg)
% weekly growth rate
Tweets, original tweets
82.2K
followers
84.9K
0.6%
0.4%
0.2%
0%
15
10
5
0
Jan
Feb
Mar
Apr
May

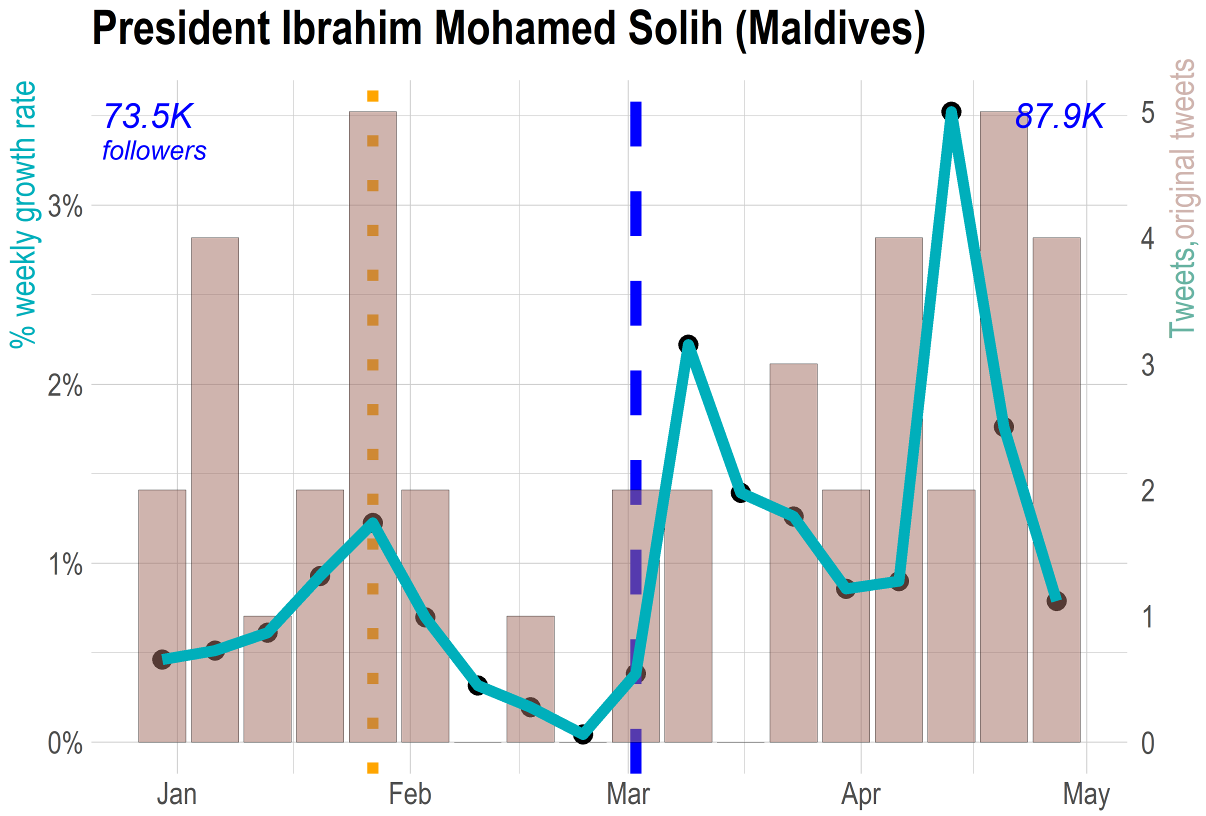

President Ibrahim Mohamed Solih (Maldives)
% weekly growth rate
Tweets, original tweets
73.5K
followers
87.9K
3%
2%
1%
0%
5
4
3
2
1
0
Jan
Feb
Mar
Apr
May

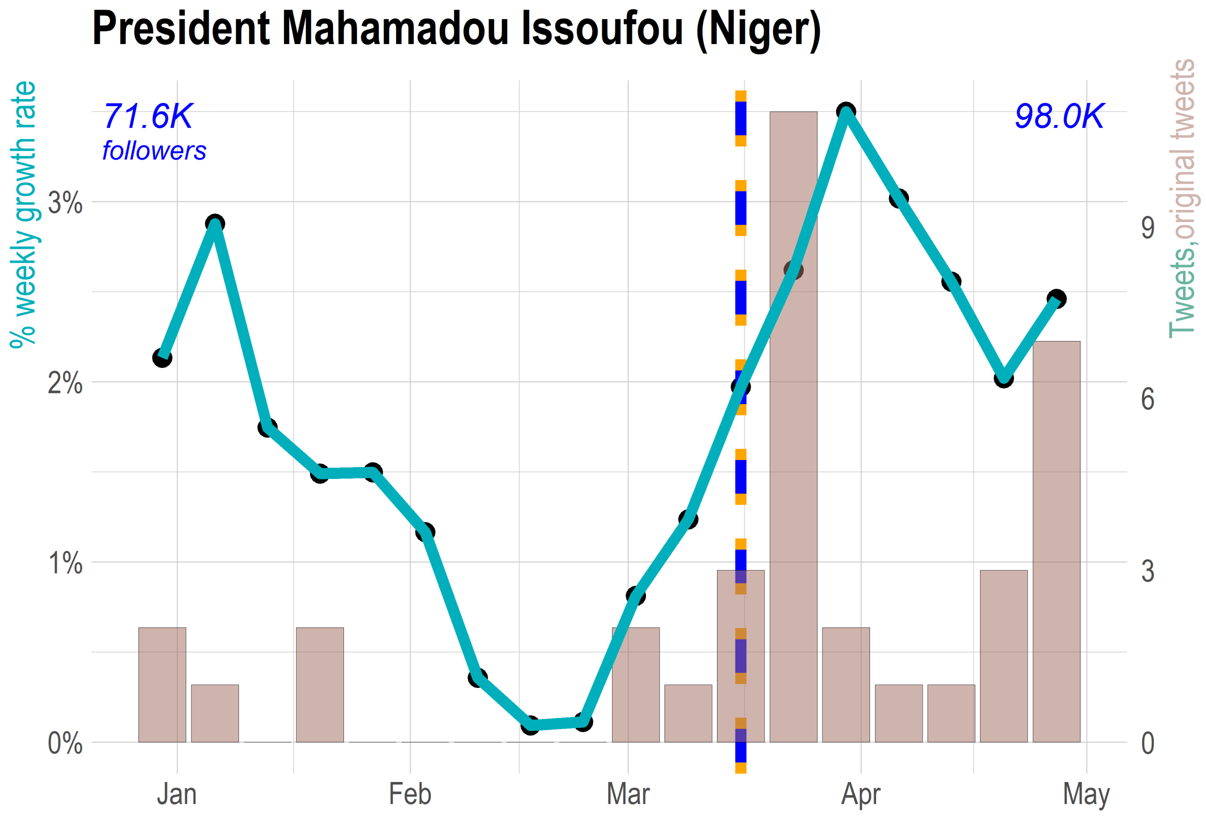
President Mahamadou Issoufou (Niger)
71.6K
followers
98.0K
% weekly growth rate
Tweets, original tweets
3%
2%
1%
0%
9
6
3
0
Jan
Feb
Mar
Apr
May

President Mokgweetsi Masisi (Botswana)

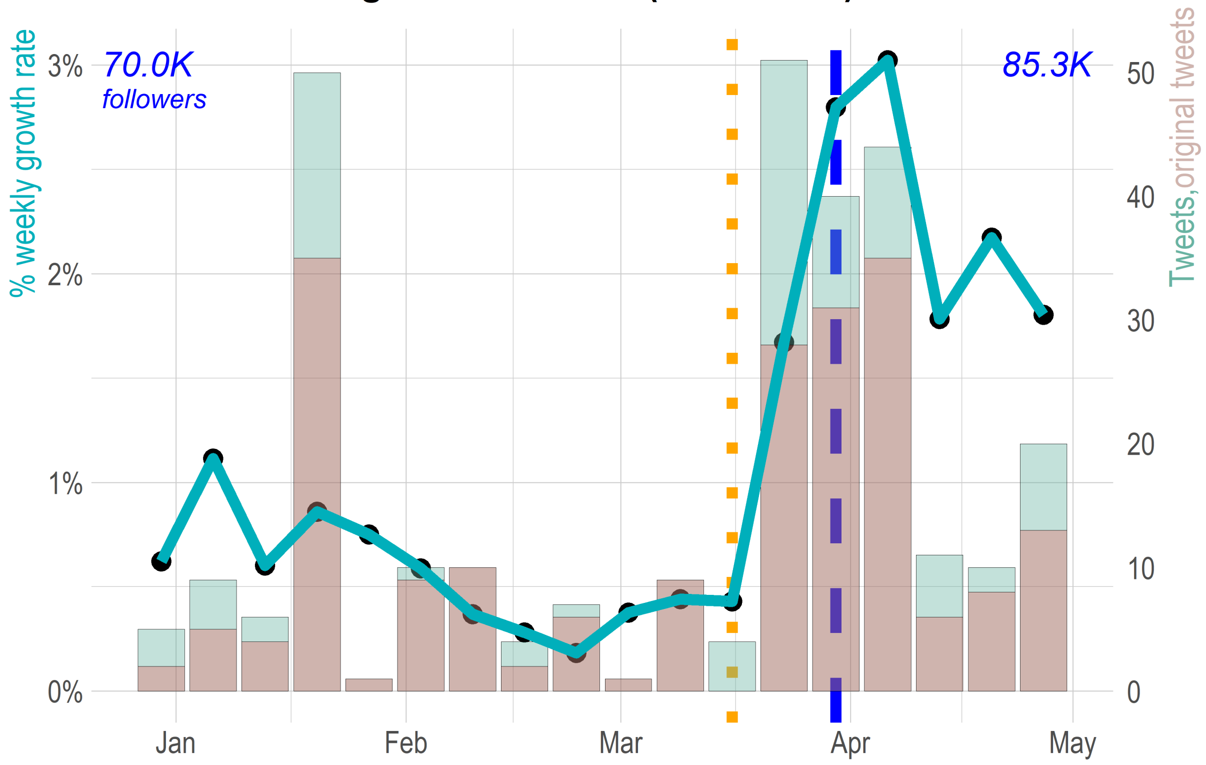

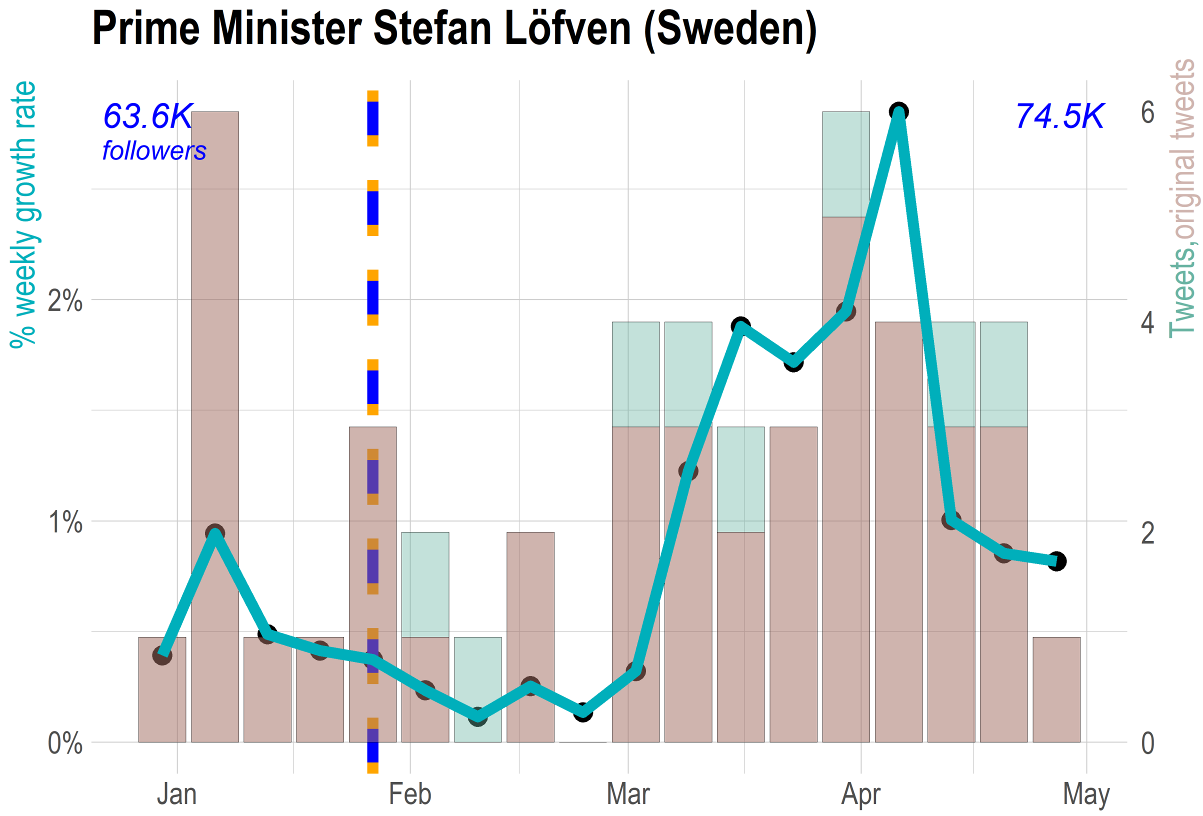
Prime Minister Stefan Löfven (Sweden)
% weekly growth rate
Tweets, original tweets
63.6K
followers
74.5K
2%
1%
0%
6
4
2
0
Jan
Feb
Mar
Apr
May

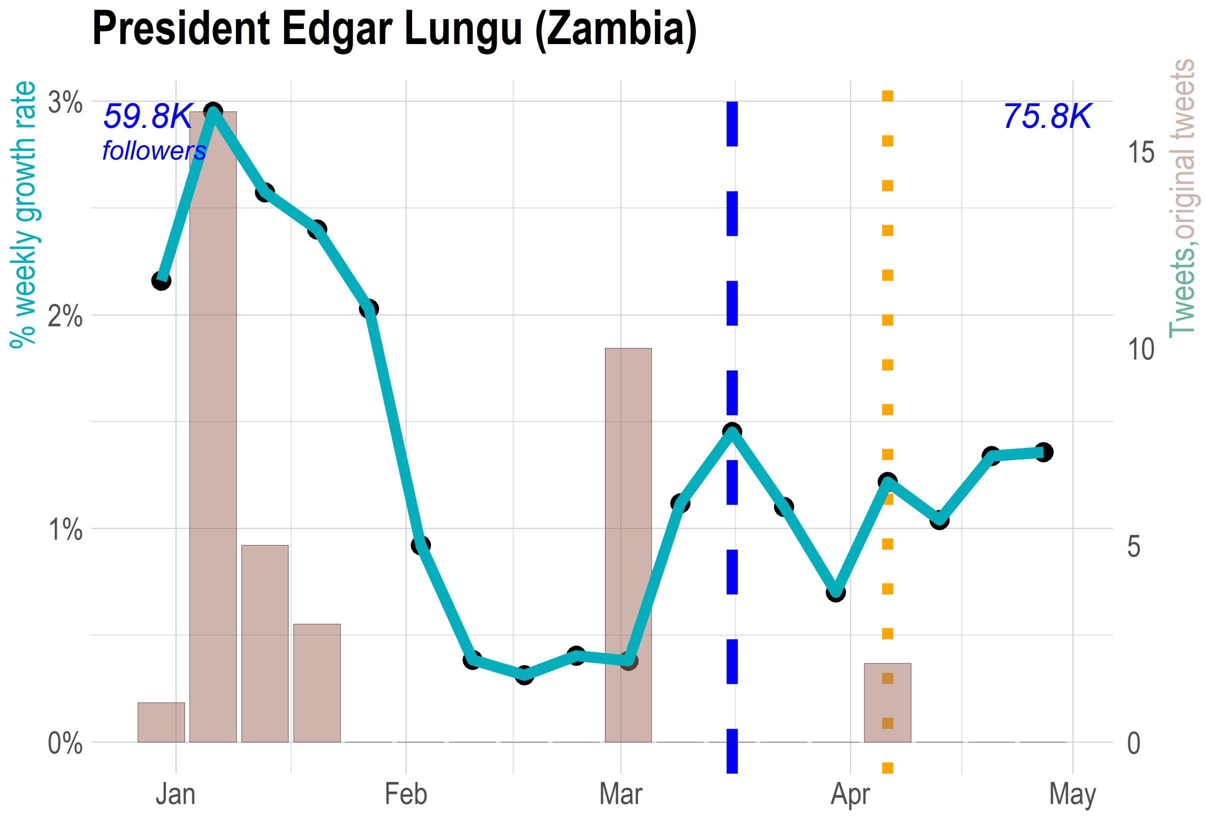

President Edgar Lungu (Zambia)
% weekly growth rate
Tweets, original tweets
59.8K
followers
75.8K
3%
2%
1%
0%
15
10
5
0
Jan
Feb
Mar
Apr
May

Prime Minister Prayut Chan-o-cha (Thailand)

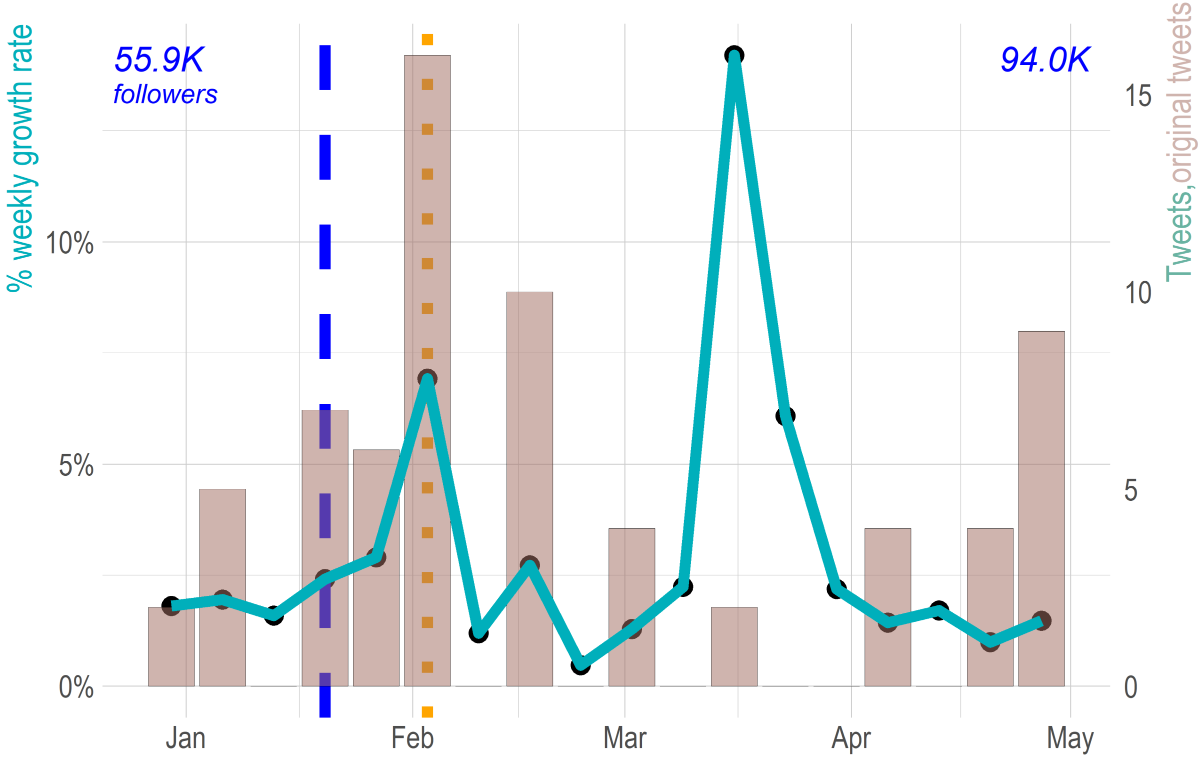

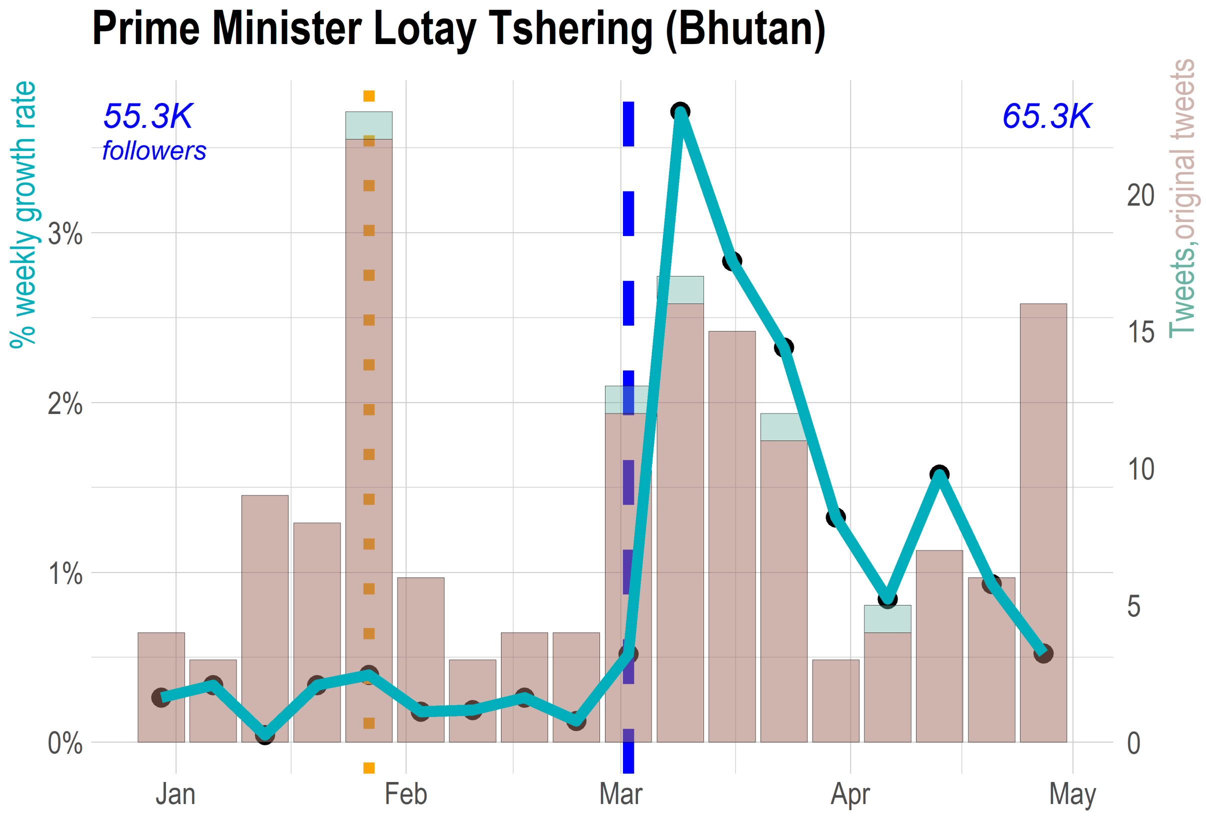
Prime Minister Lotay Tshering (Bhutan)
55.3K
followers
65.3K
% weekly growth rate
Tweets, original tweets
3%
2%
1%
0%
20
15
10
5
0
Jan
Feb
Mar
Apr
May

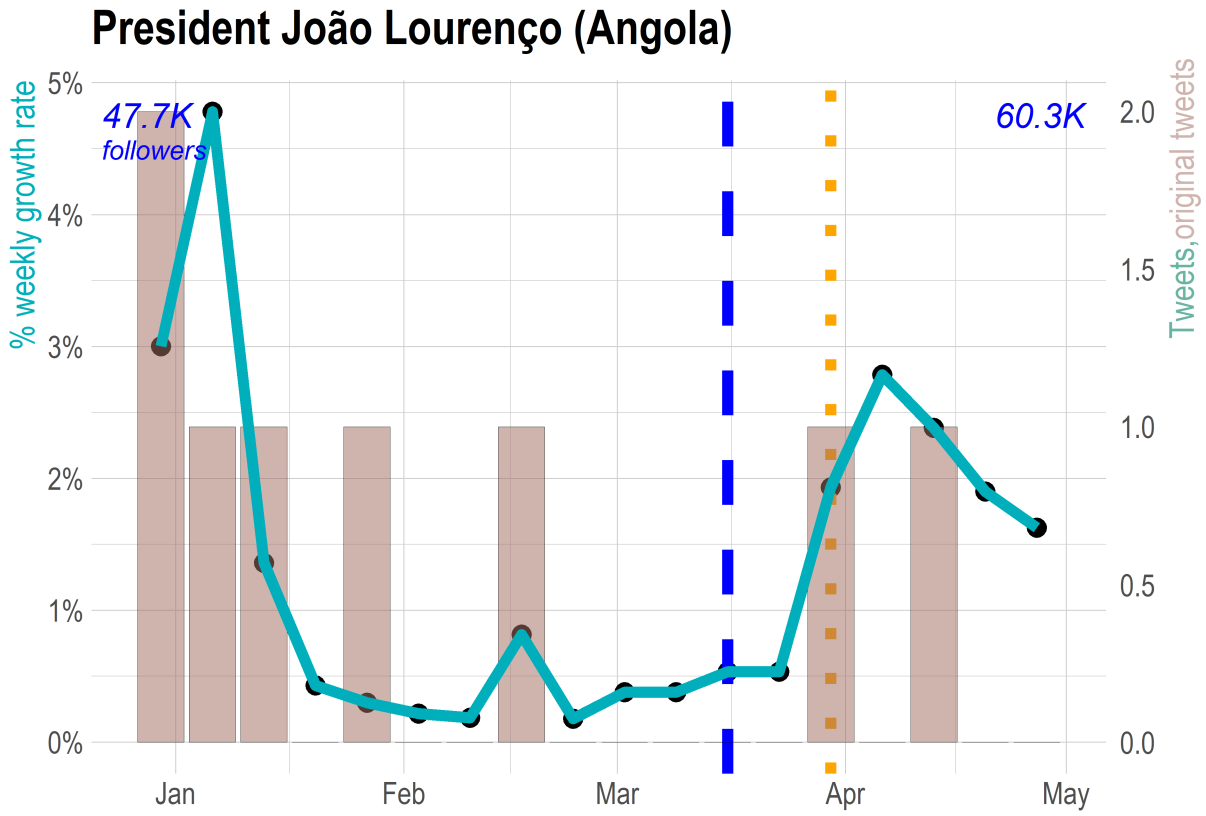
President João Lourenço (Angola)
% weekly growth rate
Tweets, original tweets
47.7K
followers
60.3K
5%
4%
3%
2%
1%
0%
2.0
1.5
1.0
0.5
0.0
Jan
Feb
Mar
Apr
May

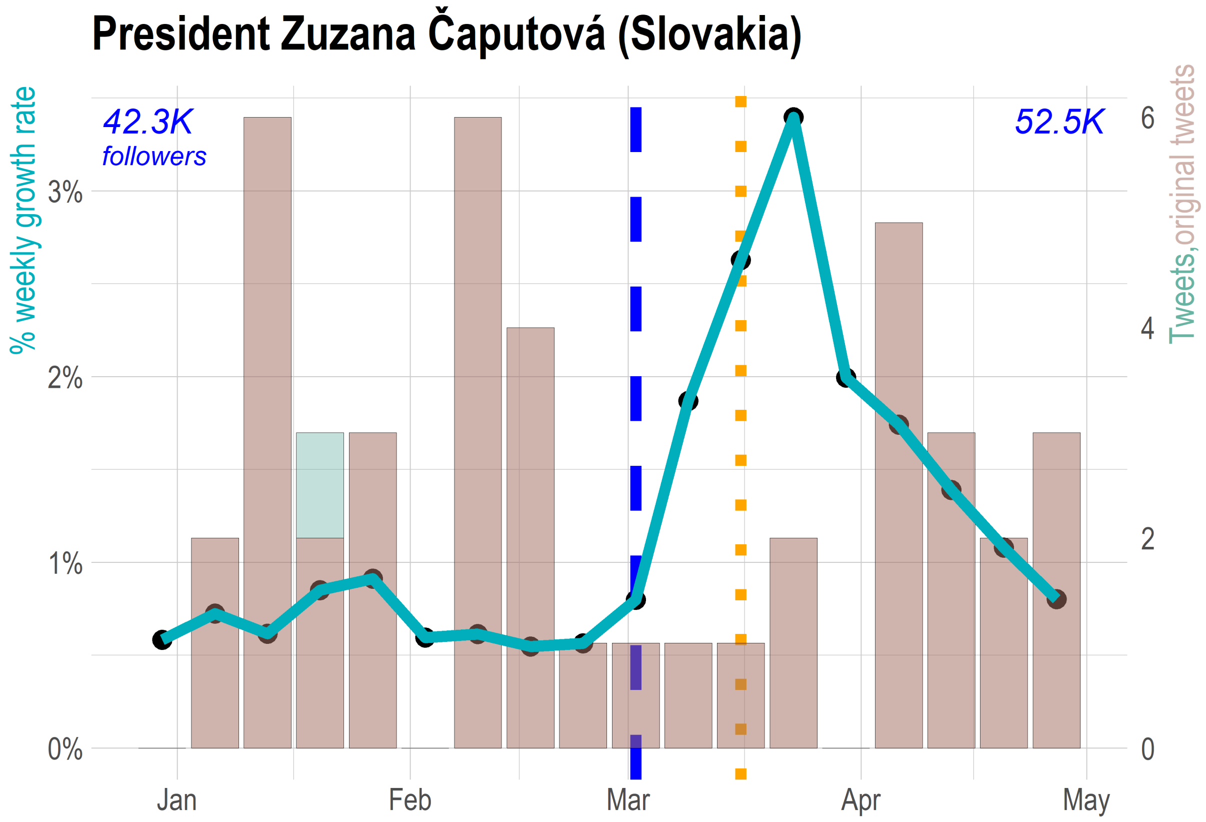

President Zuzana Čaputová (Slovakia)
% weekly growth rate
Tweets, original tweets
42.3K
followers
52.5K
0%
1%
2%
3%
0
2
4
6
Jan
Feb
Mar
Apr
May

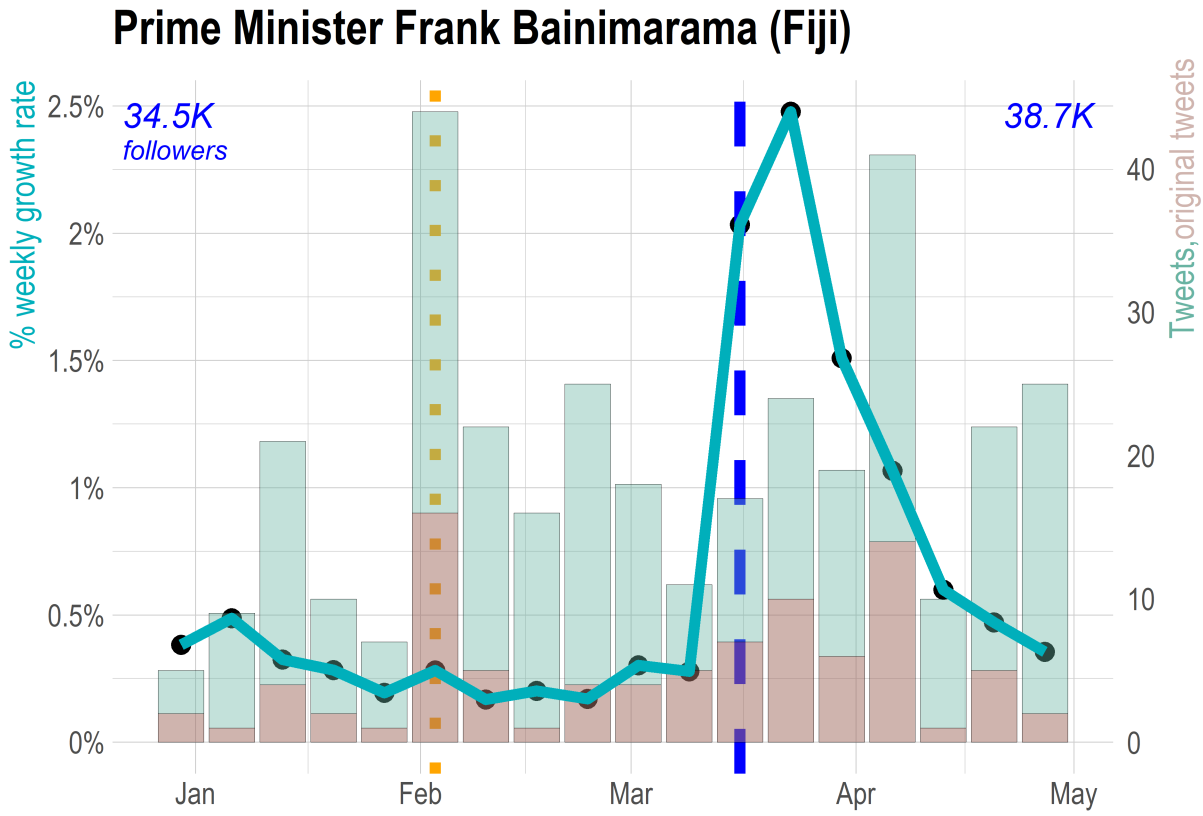

Prime Minister Frank Bainimarama (Fiji)
% weekly growth rate
Tweets, original tweets
34.5K
followers
38.7K
2.5%
2%
1.5%
1%
0.5%
0%
40
30
20
10
0
Jan
Feb
Mar
Apr
May

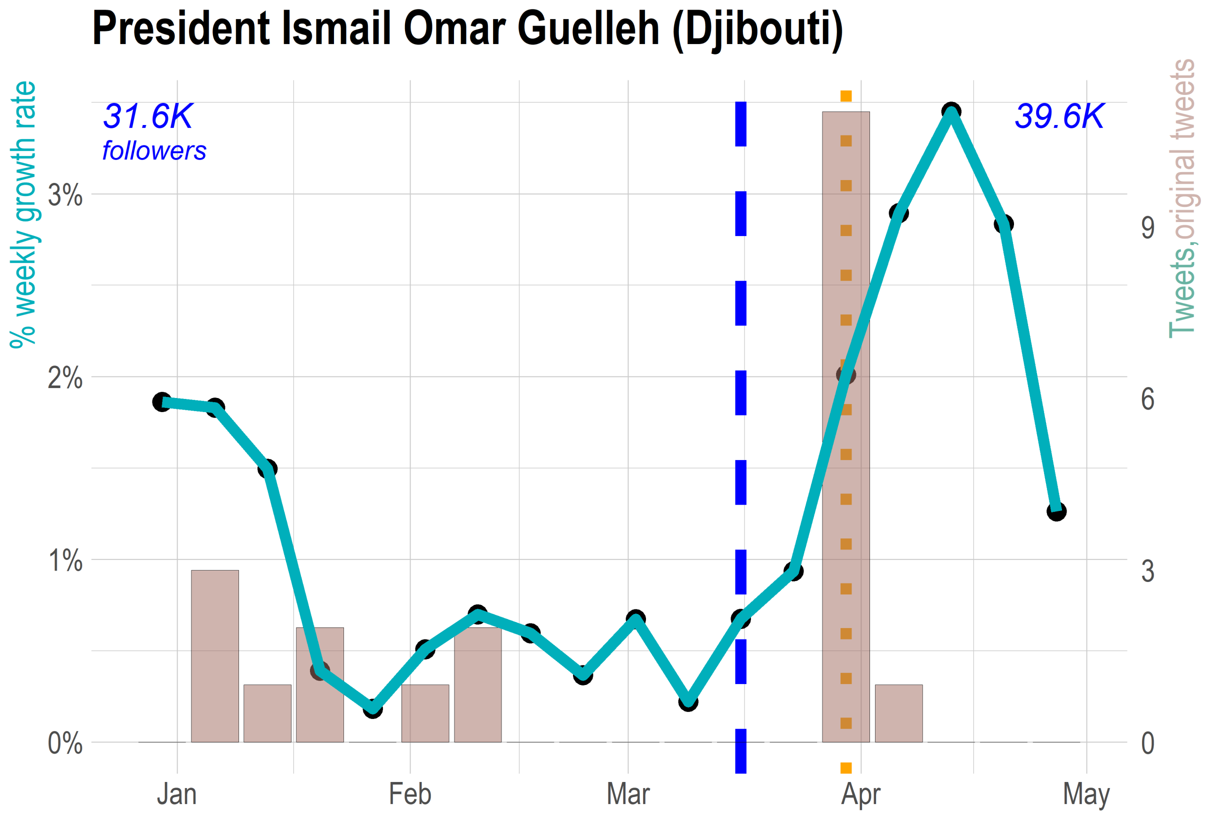

President Ismail Omar Guelleh (Djibouti)
% weekly growth rate
Tweets, original tweets
31.6K
followers
39.6K
3%
2%
1%
0%
9
6
3
0
Jan
Feb
Mar
Apr
May

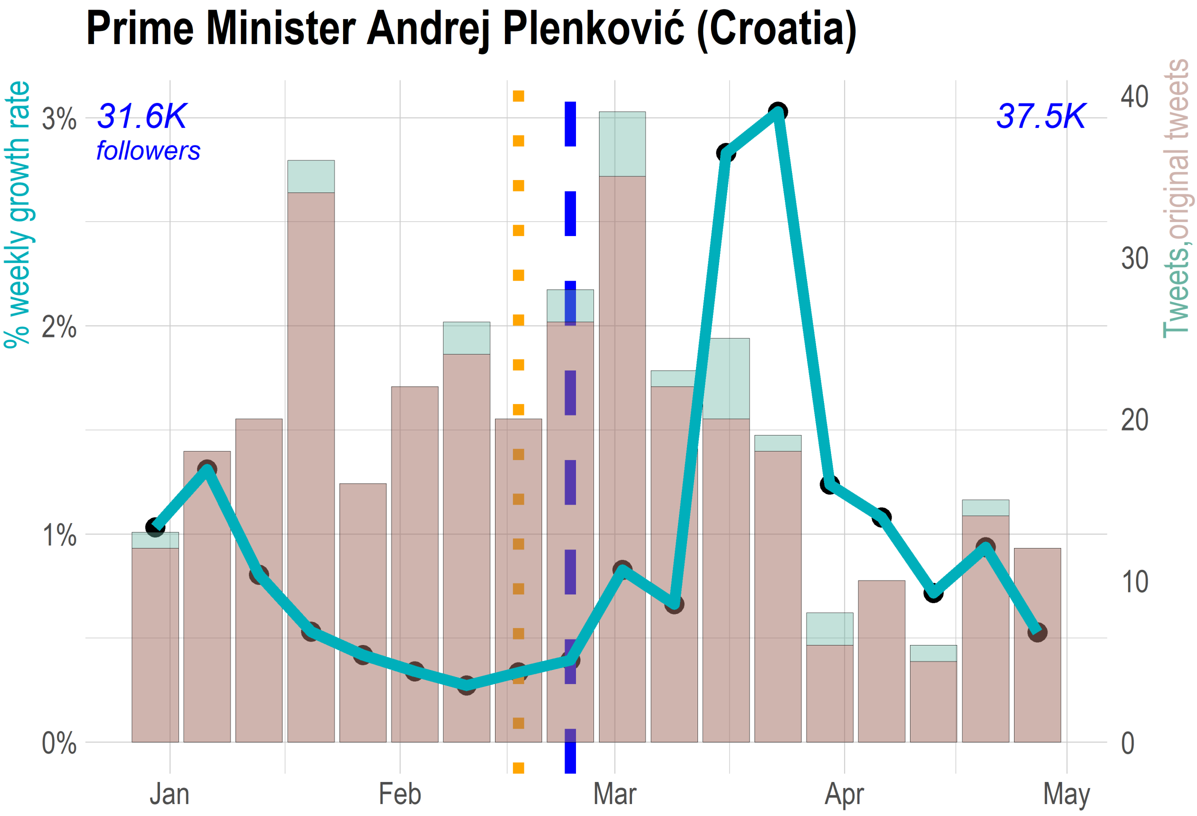
Prime Minister Andrej Plenković (Croatia)
% weekly growth rate
Tweets, original tweets
31.6K
followers
37.5K
3%
2%
1%
0%
40
30
20
10
0
Jan
Feb
Mar
Apr
May

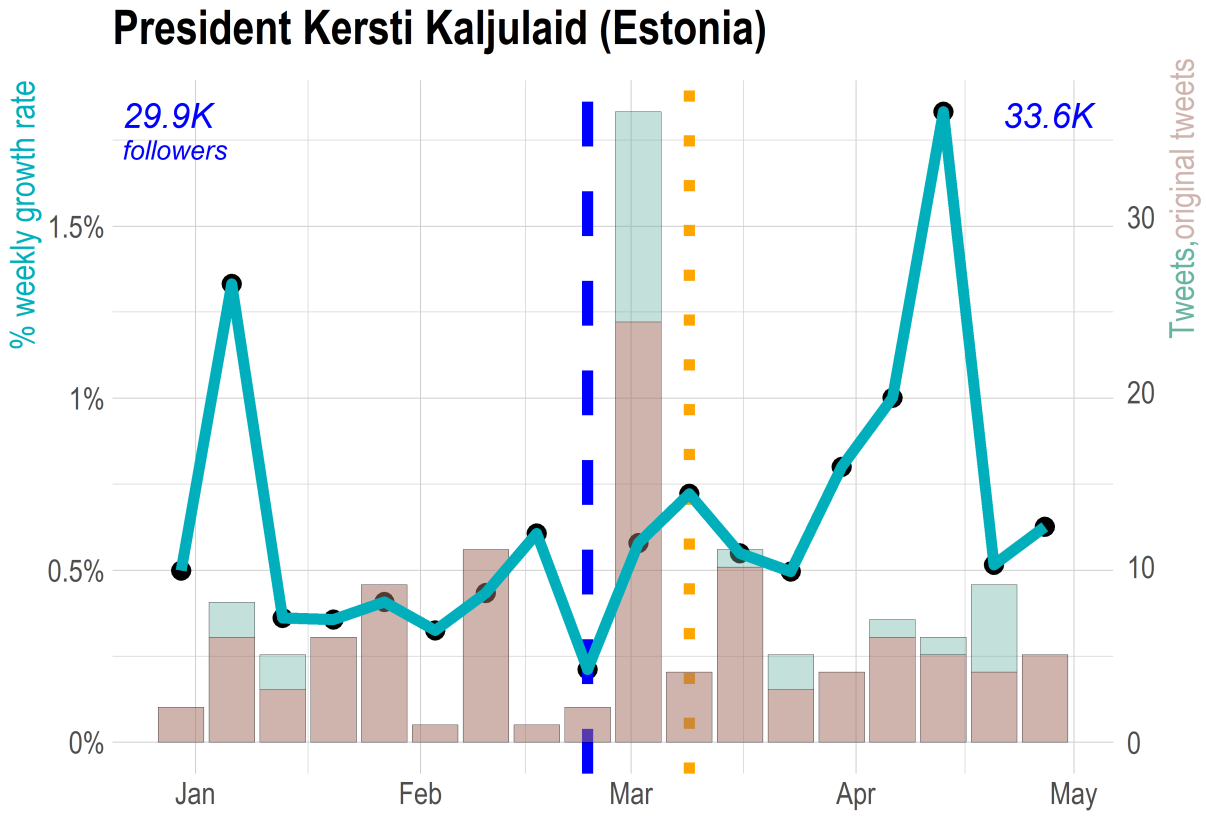
President Kersti Kaljulaid (Estonia)
% weekly growth rate
Tweets, original tweets
29.9K
followers
33.6K
0%
0.5%
1%
1.5%
0
10
20
30
Jan
Feb
Mar
Apr
May

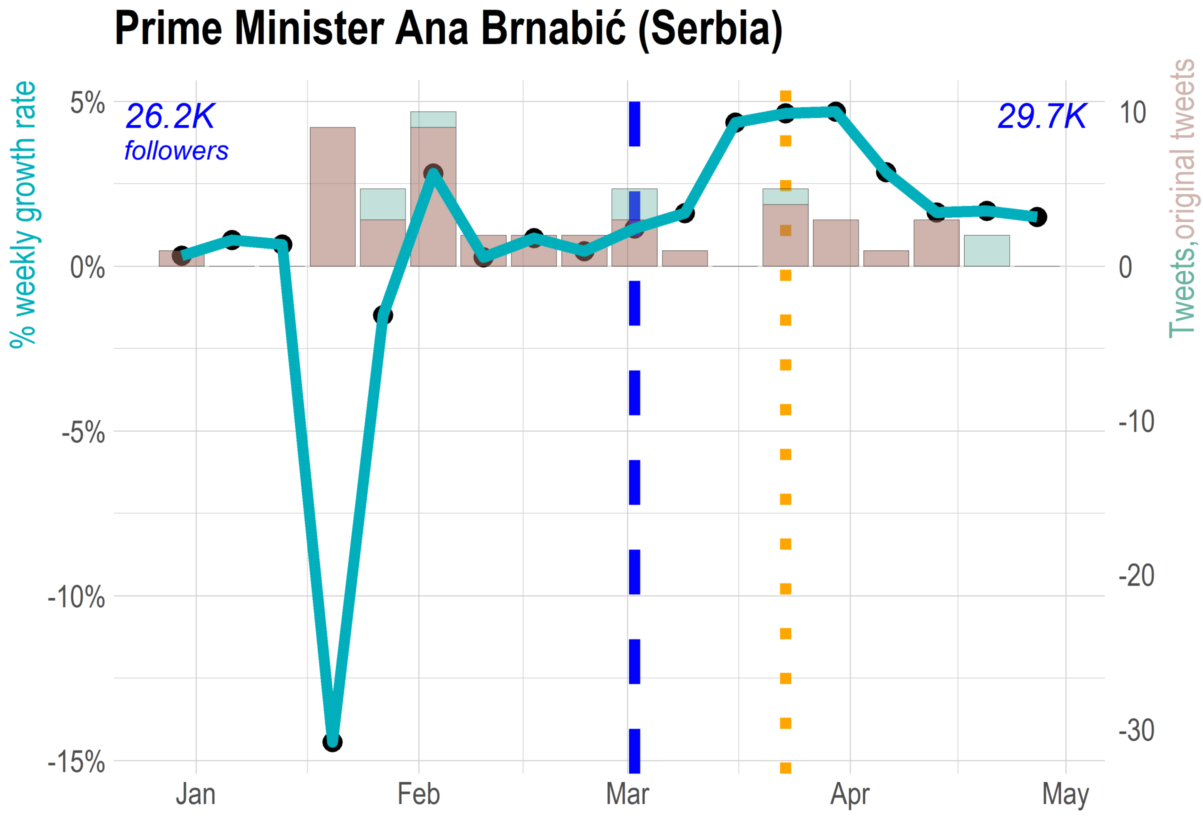
Prime Minister Ana Brnabić (Serbia)
% weekly growth rate
Tweets, original tweets
26.2K
followers
29.7K
5%
0%
-5%
-10%
-15%
10
0
-10
-20
-30
Jan
Feb
Mar
Apr
May

Prime Minister Nikol Pashinyan (Armenia)

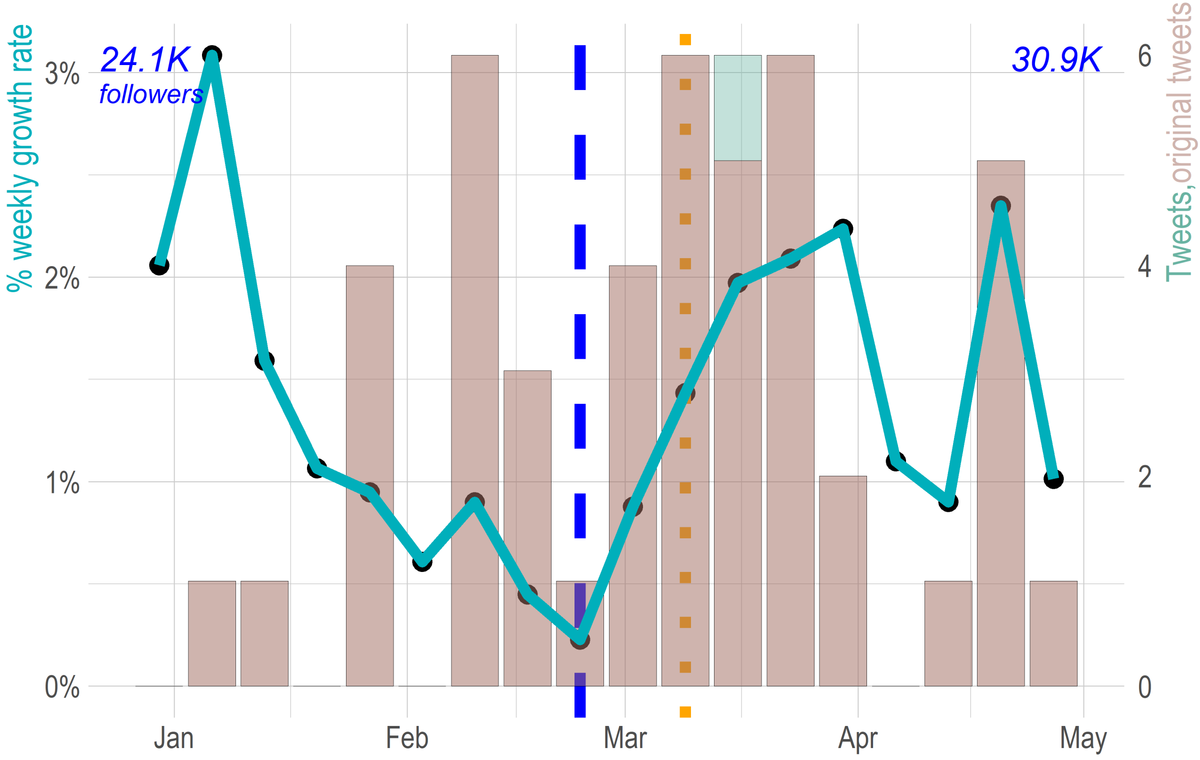

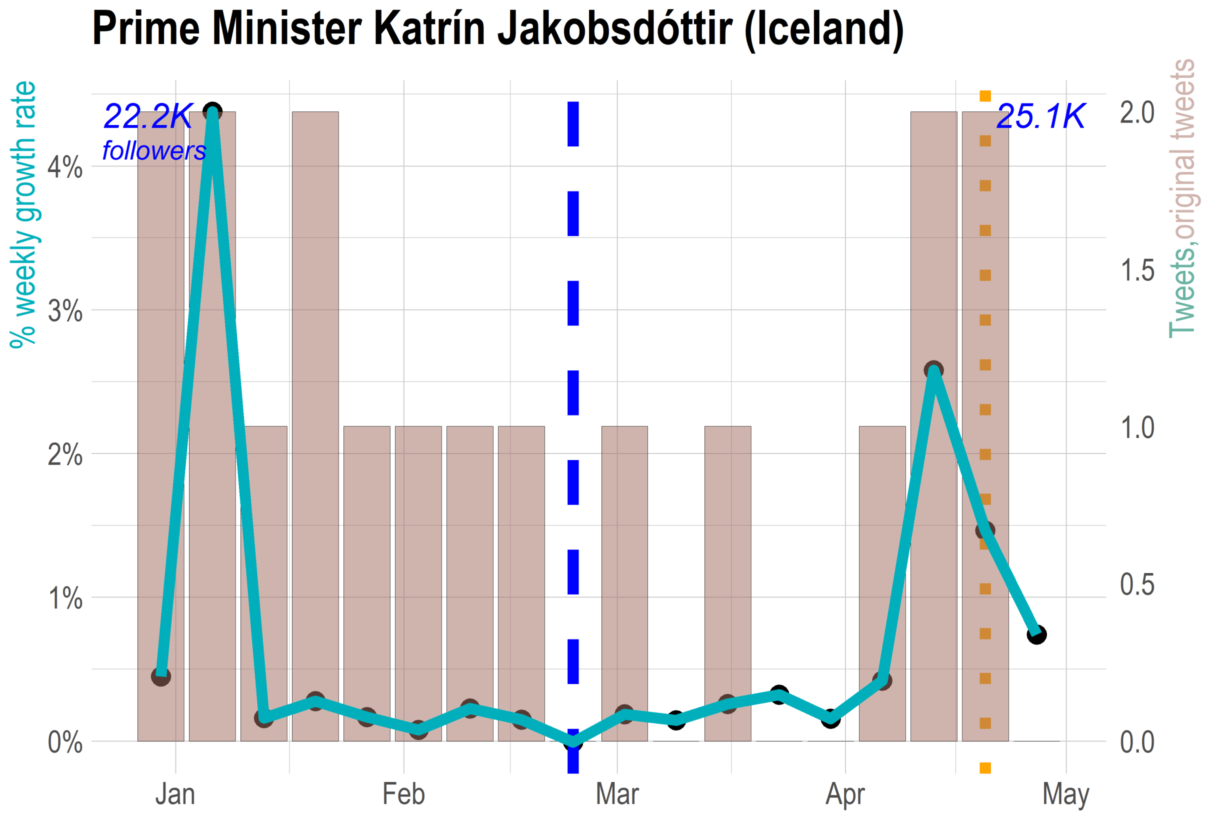

Prime Minister Katrín Jakobsdóttir (Iceland)
% weekly growth rate
Tweets, original tweets
22.2K
followers
25.1K
4%
3%
2%
1%
0%
2.0
1.5
1.0
0.5
0.0
Jan
Feb
Mar
Apr
May

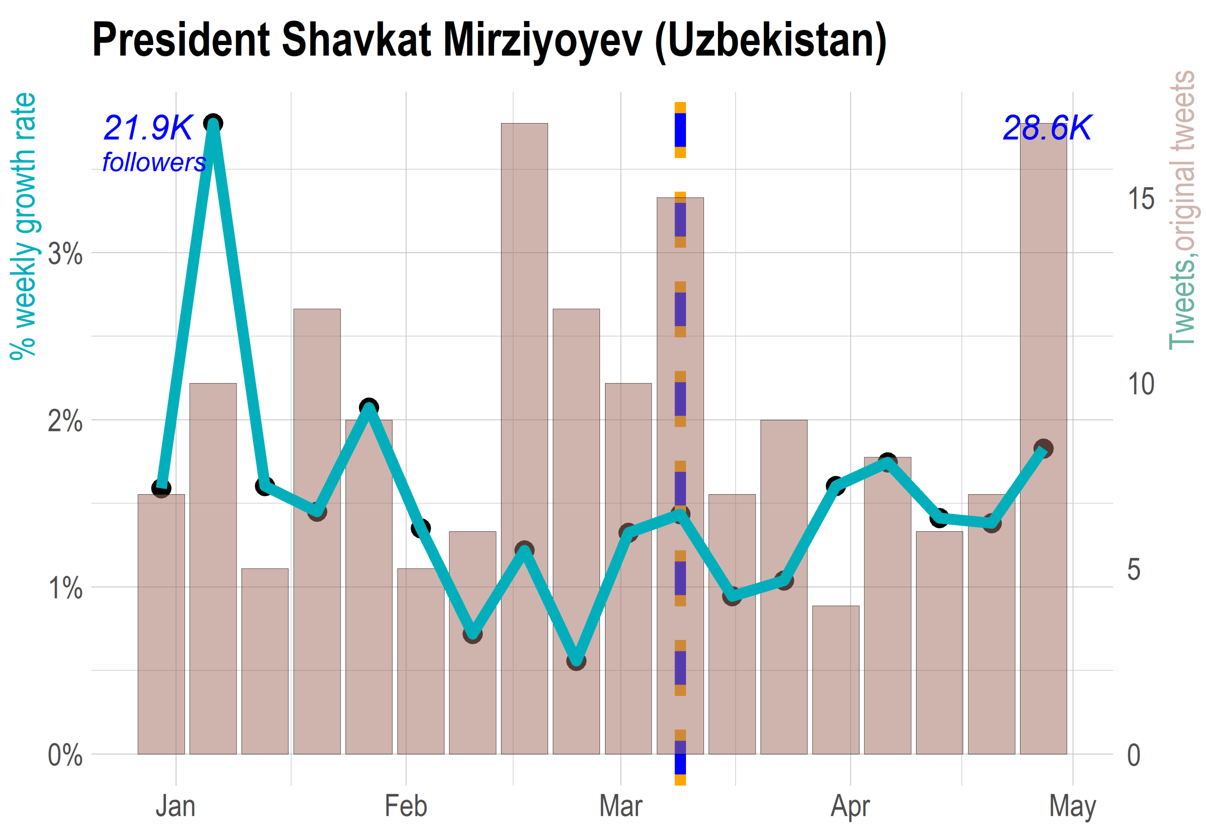

President Shavkat Mirziyoyev (Uzbekistan)
21.9K
followers
28.6K
% weekly growth rate
0%
1%
2%
3%
Tweets, original tweets
0
5
10
15
Jan
Feb
Mar
Apr
May

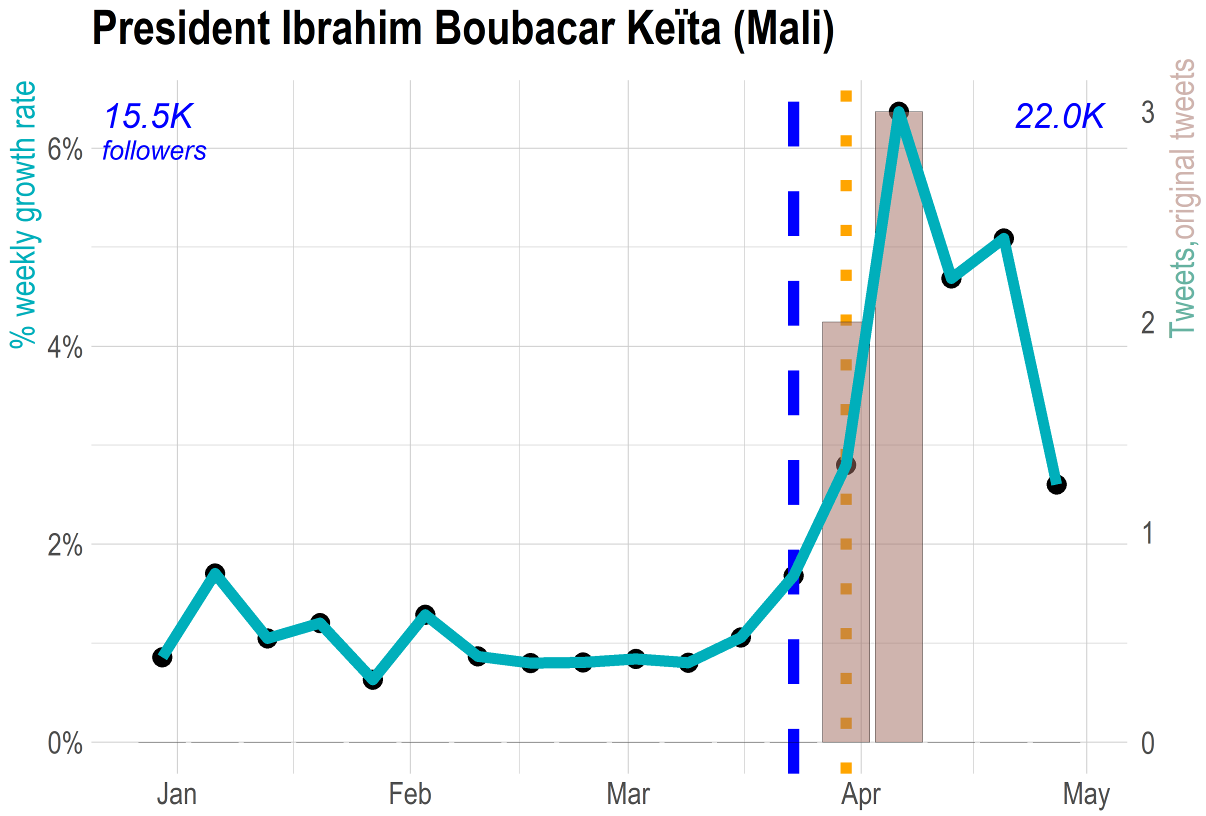
President Ibrahim Boubacar Keïta (Mali)
15.5K
followers
22.0K
% weekly growth rate
Tweets, original tweets
6%
4%
2%
0%
3
2
1
0
Jan
Feb
Mar
Apr
May

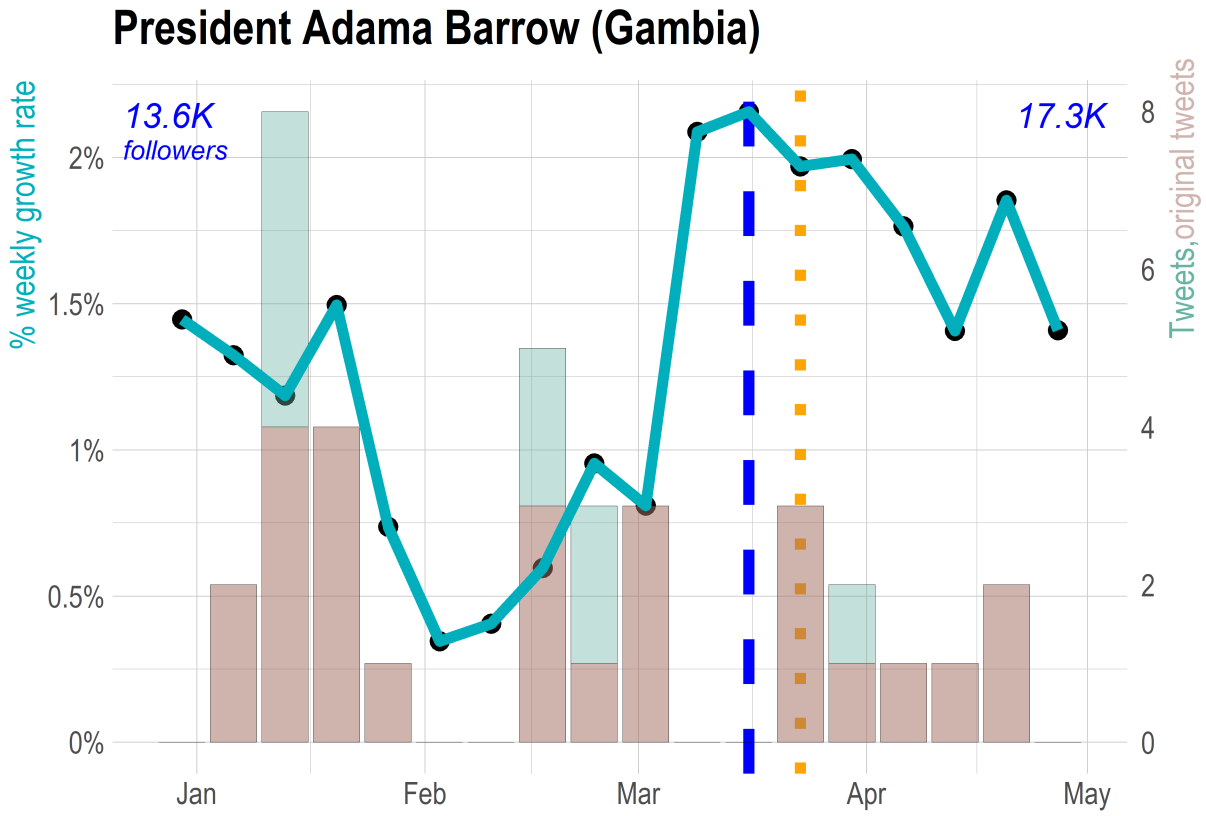

President Adama Barrow (Gambia)
% weekly growth rate
Tweets, original tweets
13.6K
followers
17.3K
2%
1.5%
1%
0.5%
0%
8
6
4
2
0
Jan
Feb
Mar
Apr
May

President Andry Rajoelina (Madagascar)

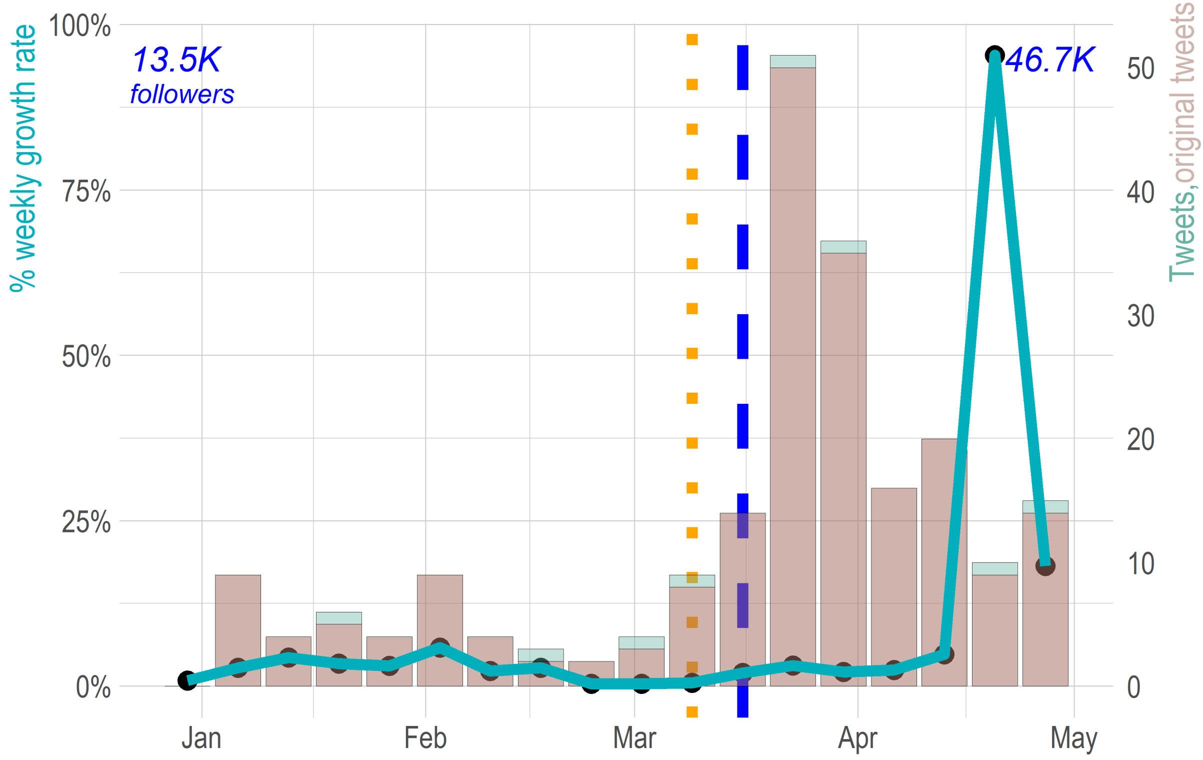

Prime Minister Krišjānis Kariņš (Latvia)

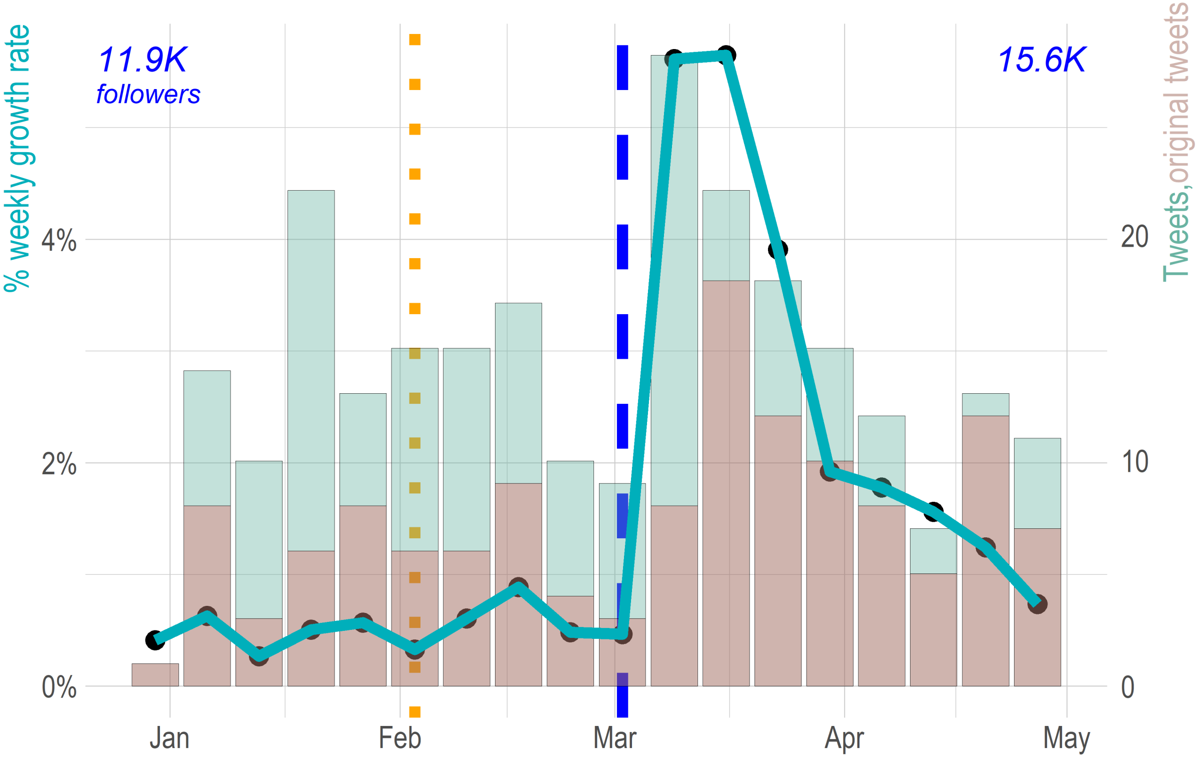

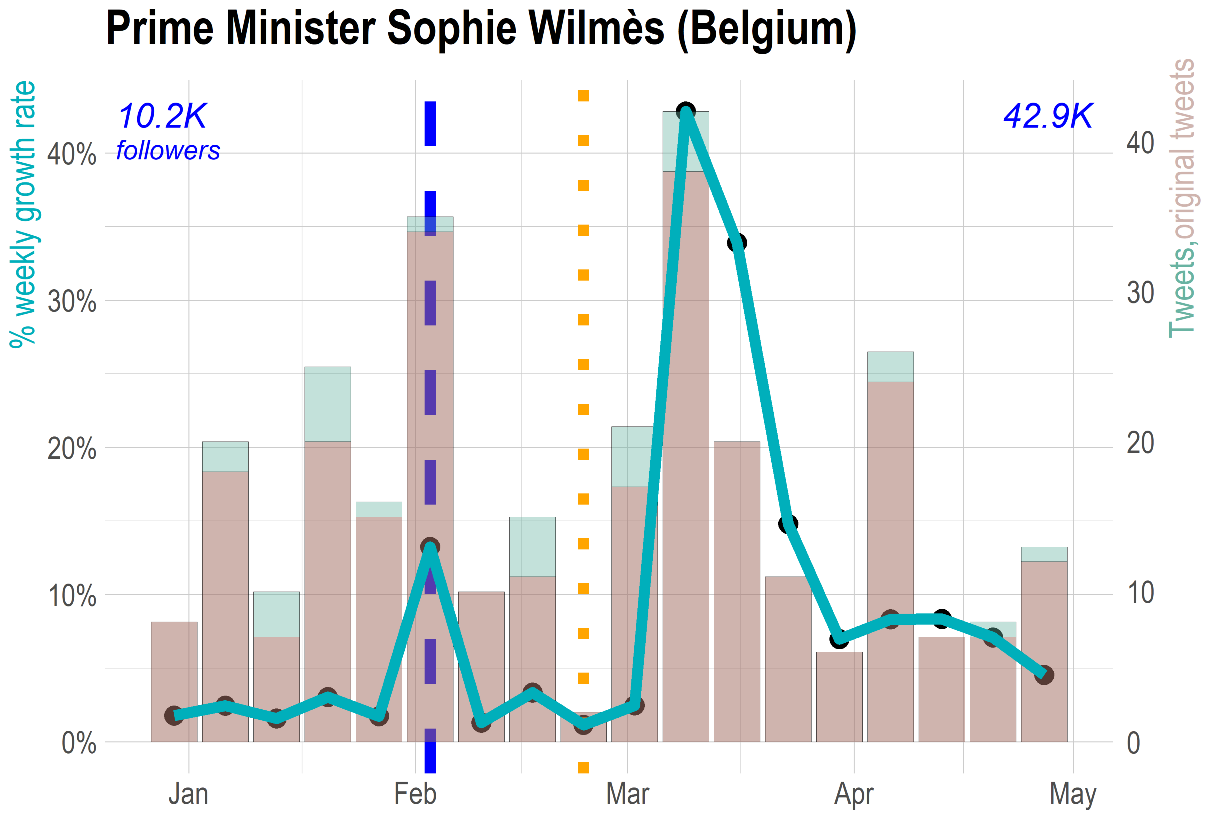
Prime Minister Sophie Wilmès (Belgium)
% weekly growth rate
Tweets, original tweets
10.2K
followers
42.9K
0%
10%
20%
30%
40%
0
10
20
30
40
Jan
Feb
Mar
Apr
May

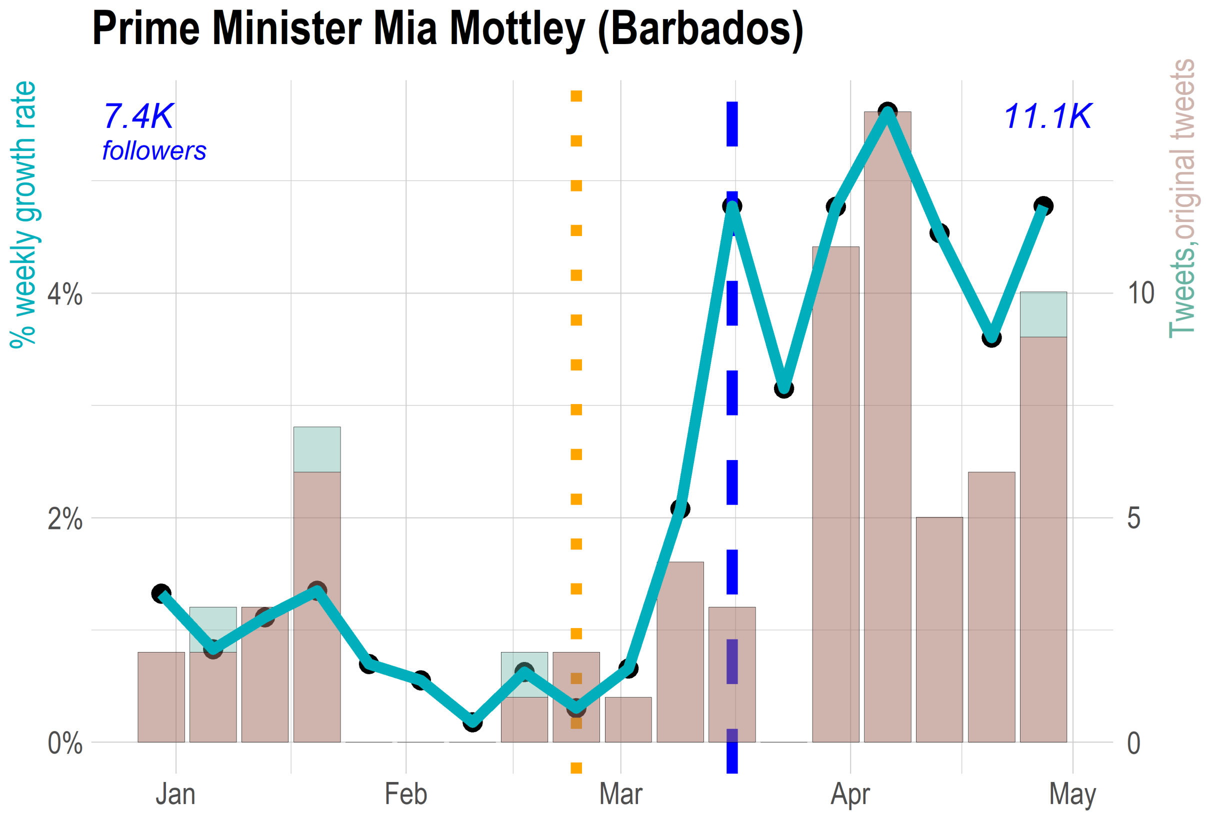
Prime Minister Mia Mottley (Barbados)
% weekly growth rate
Tweets, original tweets
7.4K
followers
11.1K
0%
2%
4%
0
5
10
Jan
Feb
Mar
Apr
May

President Gitanas Nauseda (Lithuania)

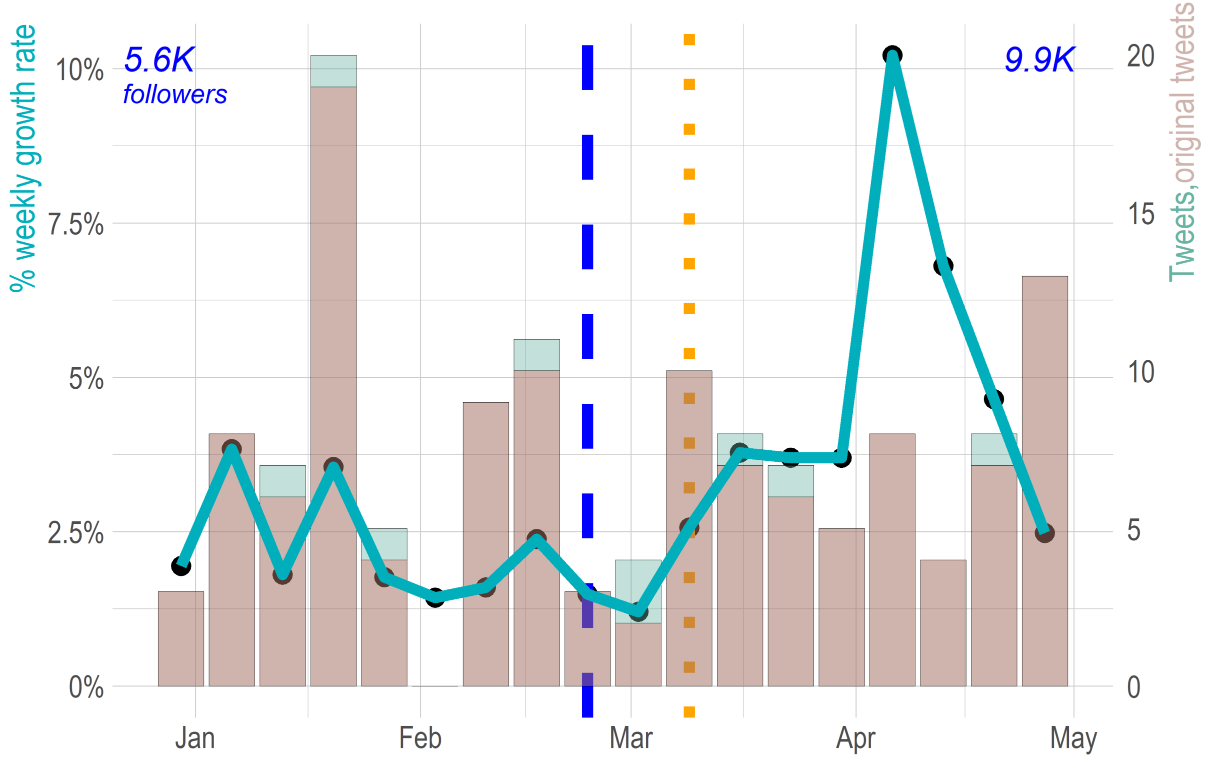

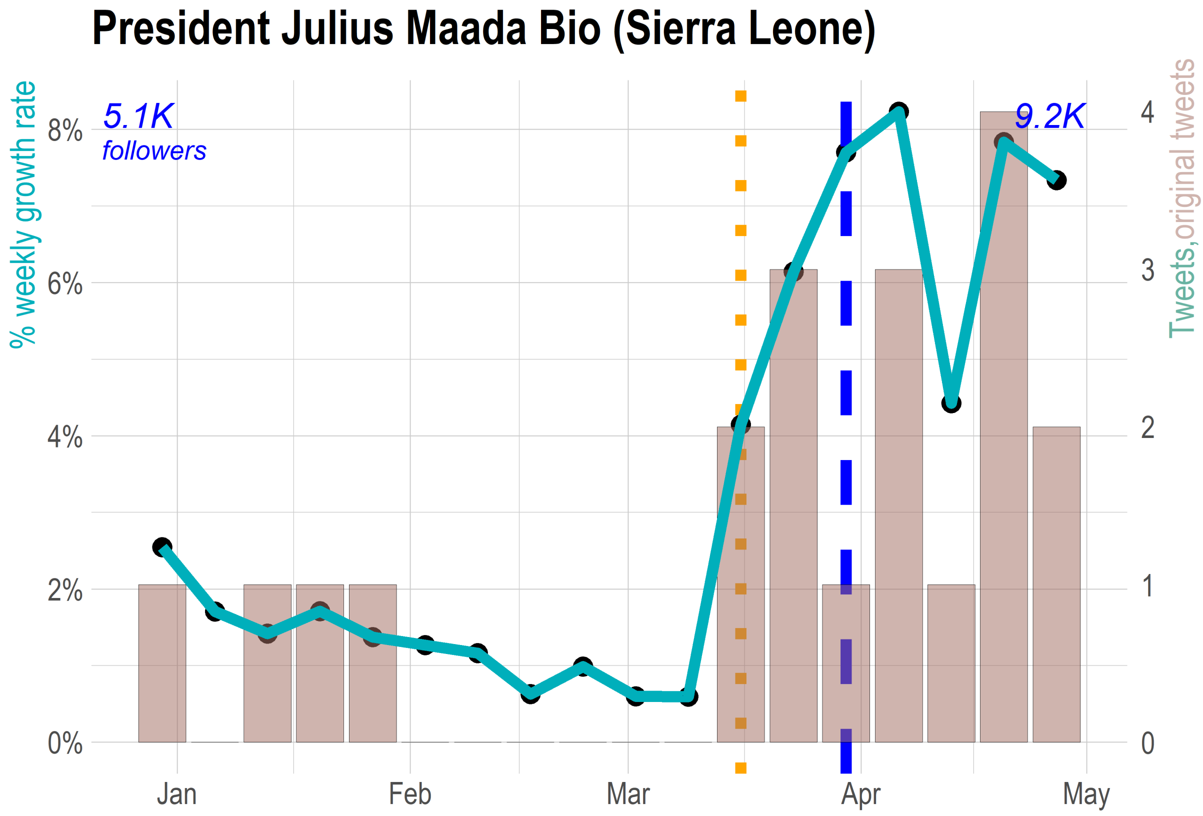

President Julius Maada Bio (Sierra Leone)
% weekly growth rate
Tweets,original tweets
5.1K
followers
9.2K
8%
6%
4%
2%
0%
4
3
2
1
0
Jan
Feb
Mar
Apr
May

Prime Minister Hubert Minnis (Bahamas)

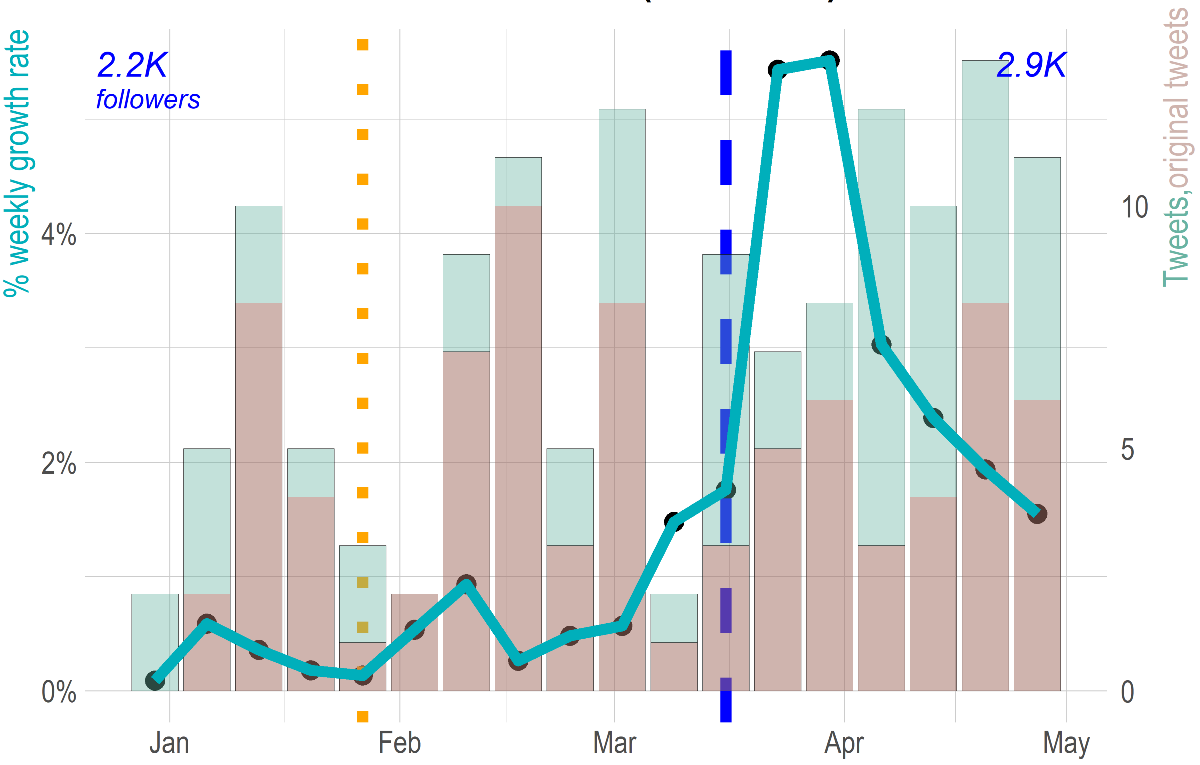

Supplement: appendix_figures02_heliyon [file mmc2.pdf]
